# A landscape of resistance gene analogs in sour cherry (*Prunus cerasus* L.)

**DOI:** 10.1186/s13104-024-06952-z

**Published:** 2024-10-06

**Authors:** Thomas Wolfgang Wöhner, Ofere Francis Emeriewen

**Affiliations:** Institute for Breeding Research on Fruit Crops, Julius Kühn Institute (JKI) – Federal Research Centre for Cultivated Plants, Dresden, 01326 Saxony Germany

**Keywords:** *P. cerasus*, Sour cherry, Resistance, NB-LRR, RLK, RLP

## Abstract

**Objective:**

This research aims to analyze the presence and distribution of resistance genes in the *avium* and *fruticosa* subgenomes of *Prunus cerasus* through computational methods and bioinformatics tools.

**Results:**

Analysis of genome and transcriptome sequencing data revealed a total of 19,570 transcripts with at least one resistance gene domain in *Prunus cerasus* subgenome *avium* and 19,142 in *Prunus cerasus* subgenome *fruticosa*. Key findings include the identification of 804 “complete” resistance gene transcripts in *Prunus cerasus* subgenome *avium* and 817 in *Prunus cerasus* subgenome *fruticosa*, with distinct distributions of resistance gene classes observed between the subgenomes. Phylogenetic analysis showed clustering of resistance genes, and unique resistance proteins were identified in each subgenome. Functional annotation comparisons with *Arabidopsis thaliana* highlighted shared and unique resistance genes, emphasizing the complexity of disease resistance in cherry species. Additionally, a higher diversity of RLKs and RLPs was observed, with 504 transcripts identified and 18 showing similarity to known reference genes.

**Supplementary Information:**

The online version contains supplementary material available at 10.1186/s13104-024-06952-z.

## Introduction

Resistance breeding against biotic stressors is increasingly important particularly because of the demand for resilient and high-quality varieties and less pesticide application. While breeding to develop such resilient varieties in annual crops can take between 10 and 20 years [1], it can last several decades in perennial crops such as fruit trees [2]. Initial successes in resistance breeding have been achieved in recent years in various fruit crops such as apple or peach [3, 4]. Breeding resistant sour cherries particularly against major pathogens like cherry leaf spot (*Blumeriella jaapii*), brown rot (*Monilinia laxa*), and bacterial canker (*Pseudomonas syringae* pv. *morsprunorum*) is one of the primary goals in sour cherry breeding [5, 6]. While many common cultivars, such as ‘Schattenmorelle,’ are susceptible to these pathogens, other sources of resistance have been identified, offering potential for breeding more tolerant or resistant varieties [6–8]. Several breeding programs have successfully released cultivars that exhibit high levels of resistance or tolerance to these diseases [5, 8, 9]. The success of a resistance-breeding program depends on the availability of not only a wide variety of genetic resources but also tools for inheritance studies of resistance genes and loci such as genomic information in the form of genome sequences and genetic maps with high-throughput genome-wide genetic markers. With the help of this information, breeders can specifically identify and characterize potential resistance (R) genes in candidate regions and develop diagnostic markers for targeted selection. Genome sequences for sour cherry varieties have recently been published [10–12]. The sour cherry (*Prunus cerasus* L.) is a tetraploid species (2n = 4x = 32) that originated from hybridization between an unreduced pollen grain of diploid sweet cherry (*P. avium*, 2n = 2x = 16) and a tetraploid ground cherry (*P. fruticosa*, 2n = 4x = 32). Therefore the genome of sour cherry is characterized by the presence of two subgenomes: *P. cerasus* subgenome *avium* and *P. cerasus* subgenome *fruticosa*. These subgenomes contribute to the species estimated 599 Mbp genome. The two main sour cherry cultivars (‘Schattenmorelle’ in Europe and ‘Montmorency’ in USA) have been sequenced and several differences were found in terms of subgenome composition and genomic complexity [10, 12]. ‘Montmorency’ is trigenomic, containing two distinct subgenomes from a *Prunus fruticosa*-like ancestor (A and A’) and two identical subgenomes from a *Prunus avium*-like ancestor (BB). In contrast, for ‘Schattenmorelle’ only two consensus subgenomes from the *P. avium*-like ancestor (B) and one from *P. fruticosa*-like ancestor (A) were assembled. However, there is no information about the nature of the resistance genes in the sour cherry genome.

Plants possess an innate immune system that essentially consists of two branches [13] to defend against pathogens, one of which is based on the recognition of pathogens by resistance proteins encoded by R-genes in individual cells. The major classes of plant resistance genes are grouped based on their functional domains e.g. NBS, LRR, CC, TIR, kinase etc. [14]. The main class of resistance proteins found in plants has an NB - nucleotide-binding site and a leucine-rich repeat (LRR) domain. NB-LRR genes can generally be divided into 3 types, which differ in the presence of a Toll/interleukin-1 receptor domain at the N-terminal end. NB-LRR genes lacking a TIR domain usually have a coiled-coil domain CC instead. A third type is RPW8-NBS-LRR, which act in downstream defense signal transduction [15, 16]. The TM-LRR is divided into 2 types, receptor like kinases (RLK) and receptor like proteins (RLP) [15, 16]. While the occurrence of resistance genes in the genomes of various *Prunus* species has been investigated [17–19], this has not yet been done for sour cherries. The aim of this research note is to examine the data published by Wöhner and colleagues [11] regarding the classes of resistance genes present in the ‘Schattenmorelle’ sour cherry genome (subgenome *avium* and subgenome *fruticosa*) and to provide their positions.

## Methods

Genome and transcriptome sequencing, along with structural and functional annotation, as well as InterProScan analysis, were previously performed as described in Wöhner et al. [10]. InterProScan results for *P. cerasus* from previously published data [11] were downloaded from the OpenAgrar repository (www.openagrar.de) and filtered in Microsoft Excel. Pfam and Superfamily tabsheets were filtered for domain-specific entries according to [20]. Entries labelled with Pfam identifiers PF18052 indicated a CC domain, PF00931 indicated the NB domain, and PF01582 indicated the TIR domain. Leucine-rich repeats were filtered out of the Superfamily tab sheet using the identifiers SSF52058, SSF52047, and “leucine-rich repeat”. Kinase domains were filtered using the GO:0004672 term in the Pfam tabsheet, and TRANSMEMBRANE or SIGNAL_PEPTIDE was used for filtering specific domains in Phobius obtained data. The LysM protein motif was filtered using IPR018392 in data obtained from ProSiteProfiles tabsheets. The data was sorted and assigned to transcript IDs obtained from the annotation of *P. cerasus* ‘Schattenmorelle’ for *P. cerasus* subgenome *avium* and *P. cerasus* subgenome *fruticosa*. Only full-length genes were summarized, and the number of genes per resistance gene class was determined. For annotation of NB-LRRs, a BLAST database was created using proteins obtained from UniProt, filtered by Gene Ontology terms (0051707 - response to other organism; 0006952 - defense response; taxonomy Embryophyta). The final transcripts of full-length resistance gene candidates were blasted against this database using BLAST p [21–23] on the Galaxy server (www.usegalaxy.org). For annotation of RLKs and RLPs, functional characterized reference genes from [15, 16] and Uniprot proteins filtered to GO:0051707 (response to other organism), GO:0006952 (defense response), GO:0004672 (kinase domain) and taxonomy Embryophyta were used to create a BLAST database. Final RLP/RLK transcripts were blasted against this database. All final full-length sequences (581 NB-LRR and 2157 RLP/RLK transcripts in total) were aligned separately using MAFFT (gap extend penalty 0.0, gap open 1.53, matrix BLOSUM62) on Galaxy [24]. The final alignment was used for phylogenetic tree construction using the Neighbor-Joining method [25–27]. The optimal tree is shown, with the percentage of replicate trees in which the associated taxa clustered together in the bootstrap test (1000 replicates) shown next to the branches. This analysis was performed with MEGA11 [28]. The final tree was color-labelled using the Interactive Tree of Life tool [29]. The full-length resistance transcripts were displayed using MapChart software [30].

## Results and discussion

**Fig. 1:**
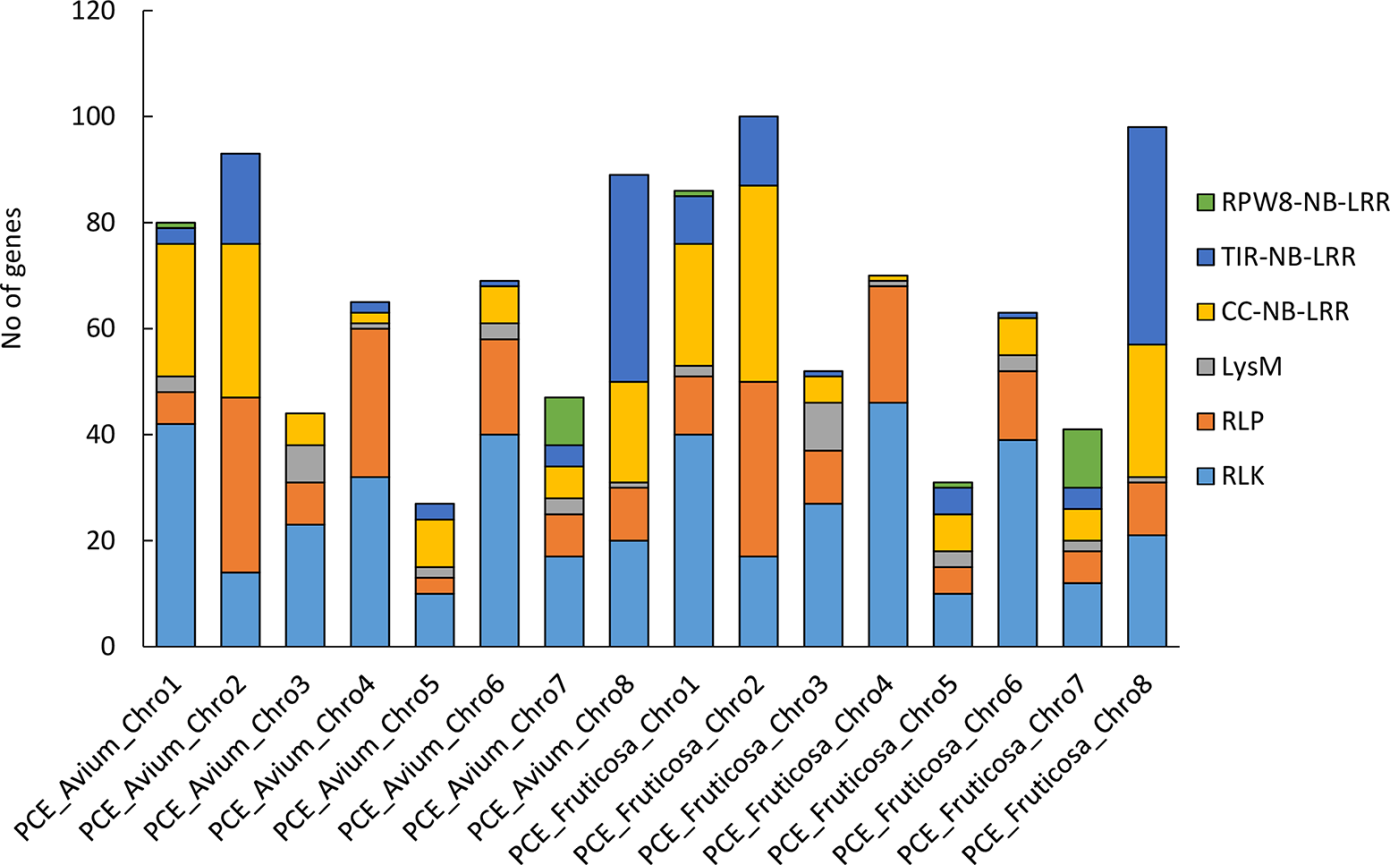
Number of identified full length resistance genes in the two subgenomes (*Prunus cerasus* subgenome *avium* and *Prunus cerasus* subgenome *fruticosa*) of *P. cerasus* ‘Schattenmorelle’. The arrow indicates the presence of RPW8-NB-LRR genes of chromosome 7 in both subgenomes

**Fig. 2:**
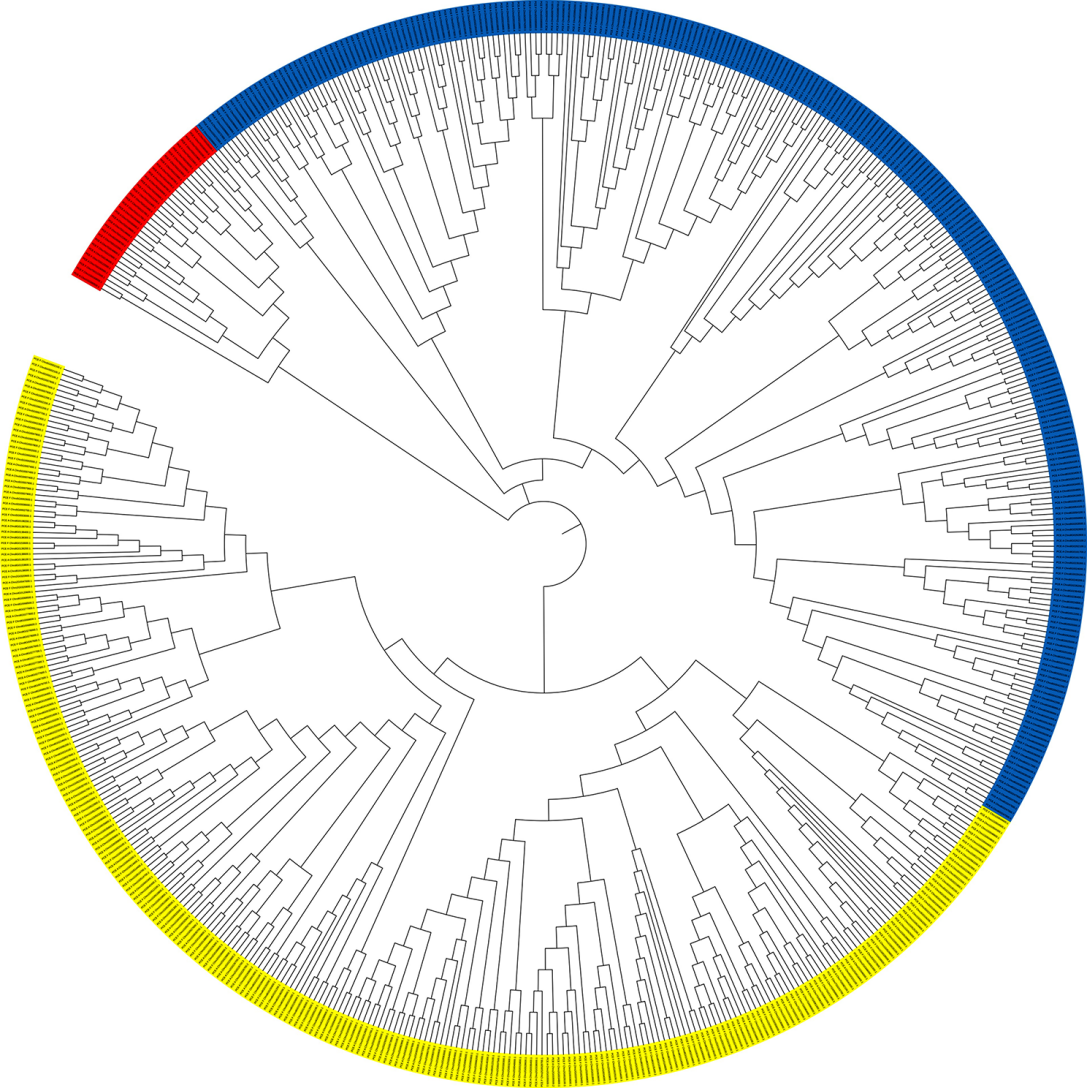
A phylogenetic tree of the 581 identified full length resistance transcripts (RPW8-NB-LRR – red; TIR-NB-LRR – blue; CC-NB-LRR – yellow)

**Fig. 3:**
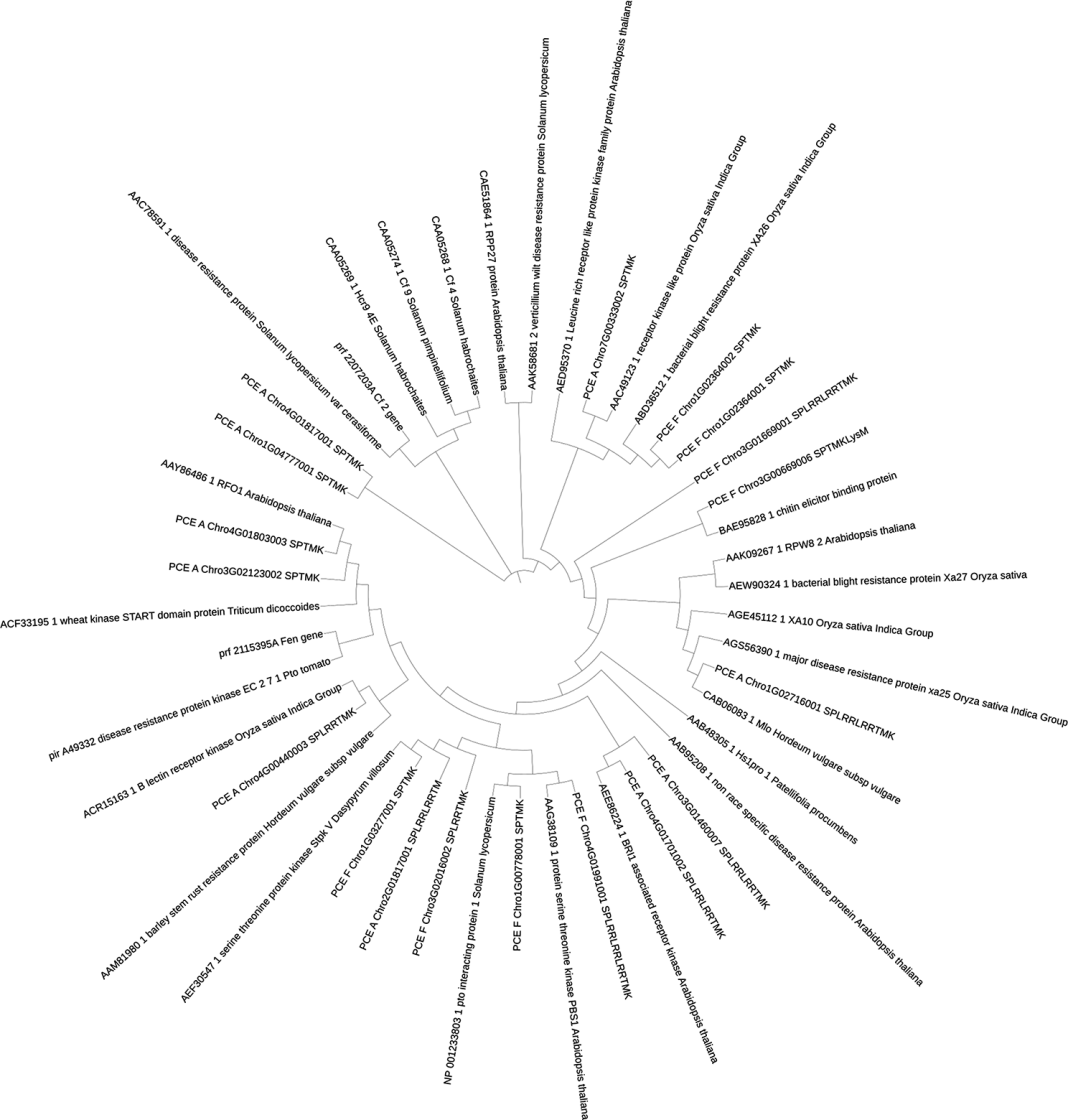
A phylogenetic tree of the 22 reference RLKs and RLPs Sehkwal et al. [15] and 18 transcripts from *P. cerasus* ‘Schattenmorelle’

A comprehensive analysis of resistance genes in *P. cerasus* subgenome *avium* and *P. cerasus* subgenome *fruticosa* revealed intriguing insights into the putative genetic basis of disease resistance in cherry species. A total of 19,570 transcripts with at least one single domain (CC, TIR, RWP8, NB, LRR, SP, TM, LysM, K) were identified in the *P. cerasus* subgenome *avium*, while 19,142 transcripts were found in the *P. cerasus* subgenome *fruticosa*. Among these, 804 (4.1%) were detected as “complete” (TIR-NB-LRR, CC-NB-LRR, RPW8-NB-LRR, RLK, RLP) in *P. cerasus* subgenome *avium*, and 817 (4.3%) in *P. cerasus* subgenome *fruticosa*. Classification of the resistance genes showed distinct distributions among the subgenomes. Since multiple splice variants may occur within one gene, we determined the number of genes and not transcripts per resistance gene type. In *P. cerasus* subgenome *avium*, 103 CC-NB-LRR, 10 RPW8-NB-LRR, 69 TIR-NB-LRR, 20 LysM, 198 RLK and 114 RLP genes were identified, whereas *P. cerasus* subgenome *fruticosa* comprised 111 CC-NB-LRR, 13 RPW8-NB-LRR, 74 TIR-NB-LRR, 21 LysM, 212 RLK and 112 RPL genes (Fig. 1). Clustering for phylogenetic analysis was performed with the transcripts of all genes determined. Phylogenetic analysis revealed clustering of resistance genes, with 34 RPW8-NB-LRR (red), 266 TIR-NB-LRR (blue), and 281 CC-NB-LRR (yellow) genes observed in a rooted tree (Fig. 2, Table S1). The plotting of the identified full-length resistance genes revealed 16 clusters (≥ 2 genes) in *P. cerasus* subgenome *avium* and 18 in *P. cerasus* subgenome *fruticosa* (Figure S1). Notably, a RPW8-NB-LRR cluster was identified on chromosome 7 in both subgenomes. Comparing the annotations obtained from *Arabidopsis thaliana*, several resistance proteins were found in both *avium* and *fruticosa* subgenomes, while some were unique to each of the subgenomes. In both subgenomes, annotations for Disease Resistance-like Proteins (*DSC1* and *DSC2*) [31] and the Suppressor of *npr1-1*, Constitutive 1 (*SNC1*) gene [32], as well as Disease Resistance Protein *TAO1* [33] were identified. Unique resistance proteins were also found in each subgenome. For instance, only the *avium* subgenome contained a homolog to Disease Resistance Protein RML1B, conferring resistance to *Leptosphaeria maculans* in *Arabidopsis thaliana* [34]. Similarly, only the *fruticosa* subgenome featured Disease Resistance Protein RPP13-like Protein 1, involved in recognizing specific pathogens such as *Pseudomonas syringae* [35]. The presence of shared and unique resistance genes underscores the complexity of disease resistance in cherry species. A higher diversity is given for RLKs and RLPs, since these play not only crucial roles in immunity but also in plant development and growth [36]. A total of 2,191 RLPs and RLKs were identified and BLAST analyses against the uniprot for the obtained proteins led to the identification of 504 RLP/RLK transcripts in *P. cerasus*. BLAST against the 22 references summarized by [9] revealed 18 transcripts as best hits (Table 1; Fig. 3). The highest percentage identity was obtained for *PCE_F_Chro1G0077800.1* with 83.8% to PTO interacting protein 1 (*Pti1*) from *Solanum lycopersicum* [31] and for *PCE_F_Chro4G0199100.1* with 81.7% to *PBS1* from *Arabidopsis thaliana* [38]. The tomato gene *Pti1* encodes a serine/threonine kinase, phosphorylated by PTO, and plays a pivotal role in the hypersensitive response (HR)-mediated signaling cascade [37]. The Arabidopsis *PBS1* resistance gene encodes a protein kinase essential for *RPS5*-mediated plant defense, with *AvrPphB* cleavage of *PBS1* and kinase activity both necessary for triggering *RPS5*-mediated resistance, while also contributing to PAMP-triggered immunity (PTI) signaling downstream of *FLS2* [36]. The analysis of resistance genes in *P. cerasus* subgenomes reveals crucial insights for breeding programs [39–41]. The identification of 19,570 and 19,142 transcripts in the subgenome *avium* and subgenome *fruticosa*, respectively, with notable clusters of resistance genes like RPW8-NB-LRR, TIR-NB-LRR, and CC-NB-LRR, provides a strong basis for targeted breeding. Unique genes, such as RML1B in the *avium* subgenome and RPP13-like in the *fruticosa* subgenome, offer potential for developing cultivars with enhanced resistance to specific pathogens. The diversity of RLKs and RLPs further supports breeding efforts aimed at improving disease resistance and overall plant health [42, 43]. These findings can guide the development of more resilient sour cherry varieties, reduce chemical treatments, and advance crop sustainability. Building on these findings, functional validation of the identified resistance genes is crucial to confirm their roles in disease resistance and to understand their potential applications in breeding programs. Identification of resistance loci through QTL mapping or GWAS is essential for pinpointing genomic regions associated with resistance. Finally, fine mapping will narrow down candidate genes, qRT-PCR will validate their expression, and functional characterization via transient expression or stable transgenesis will determine their resistance efficacy [44–47].

**Table 1:** RLKs and RLP homeologs from other studies found in *P. Cerasus* ‘Schattenmorelle’

Reference RLK/RLP from various studies summarized in Sekhwal et al. [9]	Transcript from ‘Schattenmorelle’	Type	% identity
AEF30547_1_serine_threonine_protein_kinase_Stpk_V_Dasypyrum_villosum	PCE_F_Chro1G0327700.1	RLK	49.582
ACF33195_1_wheat_kinase_START_domain_protein_Triticum_dicoccoides	PCE_A_Chro3G0212300.2	RLK	39.145
AAM81980_1_barley_stem_rust_resistance_protein_Hordeum_vulgare_subsp_vulgare	PCE_F_Chro3G0201600.2	RLK	37.143
CAB06083_1_Mlo_Hordeum_vulgare_subsp_vulgare	PCE_A_Chro1G0271600.1	RLK	37.77
prf_2207203A_Cf_2_gene	PCE_F_Chro3G0166900.1	RLK	48.116
CAA05268_1_Cf_4_Solanum_habrochaites	PCE_A_Chro4G0181700.1	RLK	32.984
AAC78591_1_disease_resistance_protein_Solanum_lycopersicum_var_cerasiforme	PCE_F_Chro3G0166900.1	RLK	43.86
CAA05274_1_Cf_9_Solanum_pimpinellifolium	PCE_A_Chro1G0477700.1	RLK	34.135
AAK58681_2_verticillium_wilt_disease_resistance_protein_Solanum_lycopersicum	PCE_A_Chro4G0181700.1	RLK	32.176
CAA05269_1_Hcr9_4E_Solanum_habrochaites	PCE_A_Chro1G0477700.1	RLK	35.545
prf_2115395A_Fen_gene	PCE_A_Chro3G0146000.7	RLK	43.11
pir_A49332_disease_resistance_protein_kinase_EC_2_7_1_Pto_tomato	PCE_A_Chro2G0181700.1	RLP	41.237
NP_001233803_1_pto_interacting_protein_1_Solanum_lycopersicum	PCE_F_Chro1G0077800.1	RLK	83.728
AAC49123_1_receptor_kinase_like_protein_Oryza_sativa_Indica_Group	PCE_A_Chro7G0033300.2	RLK	43.837
ABD36512_1_bacterial_blight_resistance_protein_XA26_Oryza_sativa_Indica_Group	PCE_F_Chro1G0236400.1	RLK	40.472
BAE95828_1_chitin_elicitor_binding_protein, _partial_Oryza_sativa_Japonica_Group	PCE_F_Chro3G0066900.6	RLP + LysM	27.852
ACR15163_1_B_lectin_receptor_kinase_Oryza_sativa_Indica_Group	PCE_A_Chro4G0044000.3	RLK	35.769
CAE51864_1_RPP27_protein_Arabidopsis_thaliana	PCE_A_Chro4G0181700.1	RLK	30.989
AAY86486_1_RFO1_Arabidopsis_thaliana	PCE_A_Chro4G0180300.3	RLK	44.754
AAG38109_1_protein_serine_threonine_kinase_PBS1_Arabidopsis_thaliana	PCE_F_Chro4G0199100.1	RLK	81.699
AED95370_1_Leucine_rich_receptor_like_protein_kinase_family_protein_Arabidopsis_thaliana	PCE_F_Chro1G0236400.2	RLK	32.598
AEE86224_1_BRI1_associated_receptor_kinase_Arabidopsis_thaliana	PCE_A_Chro4G0170100.2	RLK	49.533

### Limitations

It is important to acknowledge that while our study has provided valuable insights into the presence and distribution of resistance genes in the *P. cerasus* subgenomes *avium* and *fruticosa*; there are inherent limitations that should be considered. One key limitation is that our analysis was primarily based on computational methods, utilizing genome and transcriptome sequencing data along with bioinformatics tools for annotation and classification of resistance genes. While this approach allowed us to identify a significant number of candidate resistance genes, it is essential to recognize that the presence of these genes in the dataset does not inherently imply functional proof of their effect on known resistances. Functional validation through experimental approaches is crucial to confirm the role of these genes in mediating specific disease resistances in cherry species. Additionally, while we compared our findings with annotations from *Arabidopsis thaliana* to infer potential functions of the identified resistance genes, it is important to acknowledge that functional roles may vary amongst different plant species and environments. Subgenome bias and dosage effects may complicate the interpretation of functional validation results, particularly if gene dosage influences the observed phenotypes. The susceptibility of the Schattenmorelle cultivar suggests that the identified resistance genes might not be specific to the mentioned pathogens (e.g. *Monilinia laxa*,* Blumeriella jaapii* etc.), requiring validation in other genotypes (transgenesis). High synteny across *Prunus* species underscores the need for comparative studies to identify structural variations in candidate genes that may affect their function and resistance potential.

## Electronic supplementary material

Below is the link to the electronic supplementary material.

Supplementary Material 1

Supplementary Material 2

## Data Availability

The data collection from [11] contains the Results IPS_PCE_A.xlsx and Results IPS_PCE_F.xlsx files analysed in this note and can be downloaded from open agrar (https://www.openagrar.de/content/index.xml).

## Abbreviations

CC: Coiled coiled
TIR: Toll/interleukin-1 receptor domain
RPW8: Resistance to powdery mildew 8
NB: Nucleotide binding
LRR: Leucine rich repeat
RLK: Receptor like kinase
RLP: Receptor like protein
TM: Transmembrane domain
SP: Signal peptide
LysM: Lysin motif domain
K: Kinase
