# Supplementary figures and images for: A landscape of resistance gene analogs in sour cherry (Prunus cerasus L.)
Source: BMC Res Notes. 2024 Oct 6;17:292. doi: 10.1186/s13104-024-06952-z (PMC11457318; doi:10.1186/s13104-024-06952-z)

*P. cerasus\_ avium* - 16 cluster NB-LRR

a)

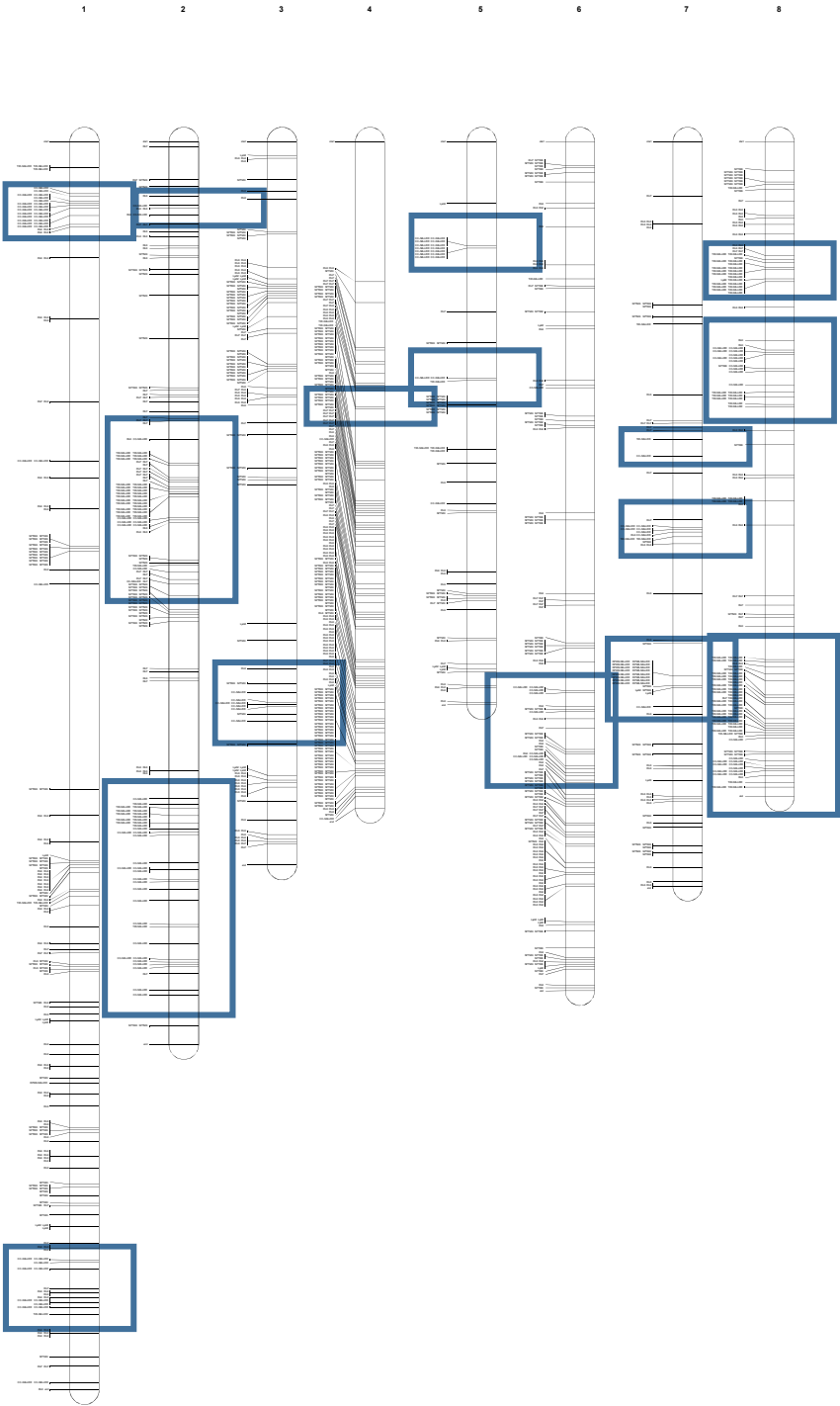

# *P. cerasus\_fruticosa* - 18 cluster NB-LRR

b)

1 2 3 4 5 6 7 8

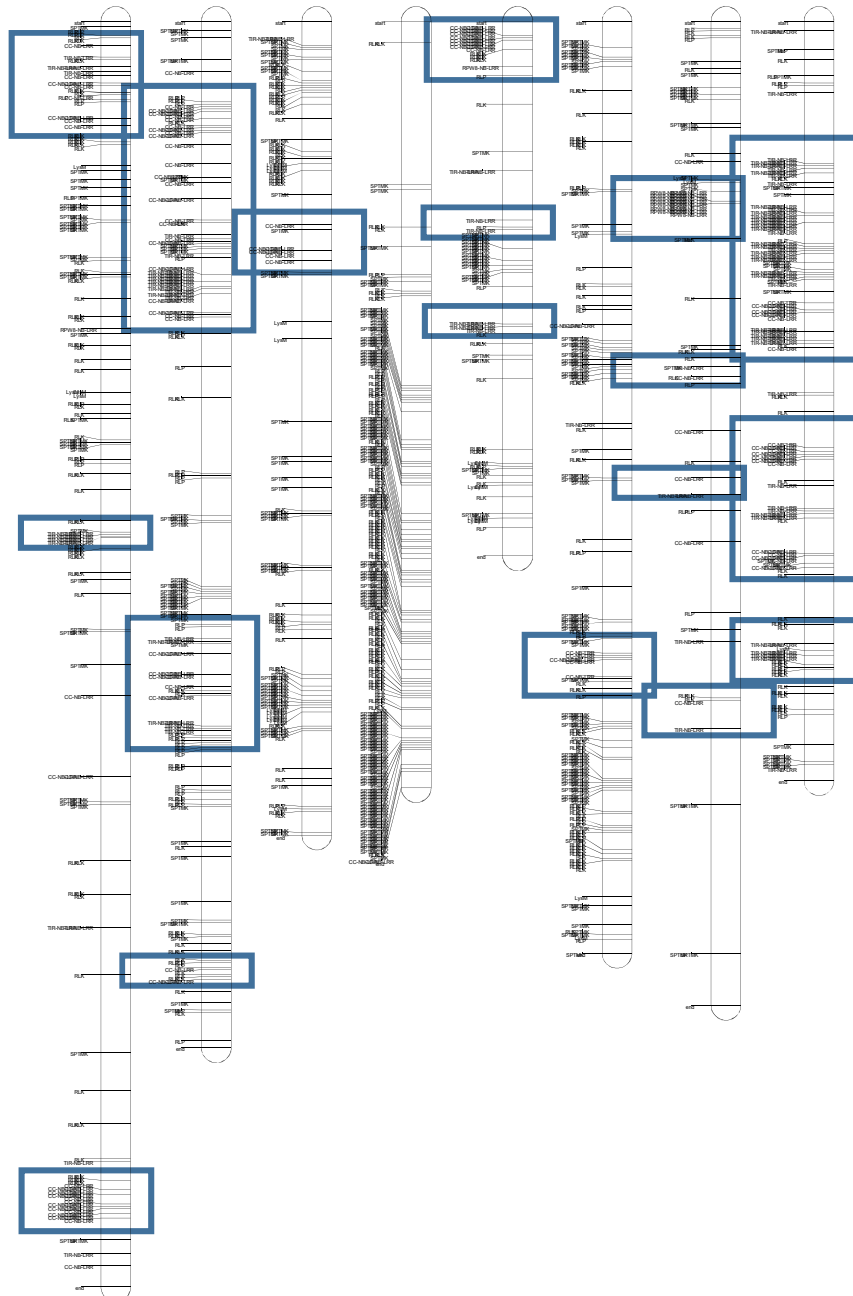

Supplement: Supplementary file 1 — Supplementary Material 1 [file 13104_2024_6952_MOESM1_ESM.pdf]
