# Supplementary material for: A landscape of resistance gene analogs in sour cherry (Prunus cerasus L.)
Source: BMC Res Notes. 2024 Oct 6;17:292. doi: 10.1186/s13104-024-06952-z (PMC11457318; doi:10.1186/s13104-024-06952-z)
Supplement: Supplementary file 2 — Supplementary Material 2 [file 13104_2024_6952_MOESM2_ESM.docx]

**Table S1** Transcript ID, subgenome (*Pce_avium* or *Pce_*fruticosa) and chromosome, position (base pairs – bp) and resistance gene type of identified full length resistance genes in *P. cerasus* 'Schattenmorelle'*.*

| Transkript ID | Genom_chromosome | start (bp) | end (bp) | Type |
| --- | --- | --- | --- | --- |
| PCE_A_Chro1G0015000.1 | PCE_Avium_Chro1 | 1101202 | 1105617 | TIR-NB-LRR |
| PCE_A_Chro1G0015000.2 | PCE_Avium_Chro1 | 1101202 | 1105617 | TIR-NB-LRR |
| PCE_A_Chro1G0015000.3 | PCE_Avium_Chro1 | 1101202 | 1105617 | TIR-NB-LRR |
| PCE_A_Chro1G0030700.1 | PCE_Avium_Chro1 | 2236404 | 2240622 | CC-NB-LRR |
| PCE_A_Chro1G0031300.1 | PCE_Avium_Chro1 | 2306980 | 2313605 | CC-NB-LRR |
| PCE_A_Chro1G0032500.1 | PCE_Avium_Chro1 | 2467923 | 2472099 | CC-NB-LRR |
| PCE_A_Chro1G0032500.2 | PCE_Avium_Chro1 | 2467923 | 2472099 | CC-NB-LRR |
| PCE_A_Chro1G0032500.3 | PCE_Avium_Chro1 | 2467923 | 2472099 | CC-NB-LRR |
| PCE_A_Chro1G0035700.1 | PCE_Avium_Chro1 | 2640911 | 2657434 | CC-NB-LRR |
| PCE_A_Chro1G0035800.1 | PCE_Avium_Chro1 | 2660987 | 2665320 | CC-NB-LRR |
| PCE_A_Chro1G0035800.2 | PCE_Avium_Chro1 | 2660987 | 2665320 | CC-NB-LRR |
| PCE_A_Chro1G0035800.3 | PCE_Avium_Chro1 | 2660987 | 2665320 | CC-NB-LRR |
| PCE_A_Chro1G0035800.4 | PCE_Avium_Chro1 | 2660987 | 2665320 | CC-NB-LRR |
| PCE_A_Chro1G0035800.5 | PCE_Avium_Chro1 | 2660987 | 2665320 | CC-NB-LRR |
| PCE_A_Chro1G0036900.1 | PCE_Avium_Chro1 | 2748104 | 2752977 | CC-NB-LRR |
| PCE_A_Chro1G0037600.1 | PCE_Avium_Chro1 | 2781724 | 2785155 | CC-NB-LRR |
| PCE_A_Chro1G0037600.2 | PCE_Avium_Chro1 | 2781724 | 2785155 | CC-NB-LRR |
| PCE_A_Chro1G0038000.1 | PCE_Avium_Chro1 | 2797771 | 2802175 | CC-NB-LRR |
| PCE_A_Chro1G0038000.2 | PCE_Avium_Chro1 | 2797771 | 2802175 | CC-NB-LRR |
| PCE_A_Chro1G0041700.1 | PCE_Avium_Chro1 | 3071121 | 3077664 | CC-NB-LRR |
| PCE_A_Chro1G0041700.2 | PCE_Avium_Chro1 | 3071121 | 3077664 | CC-NB-LRR |
| PCE_A_Chro1G0042200.1 | PCE_Avium_Chro1 | 3104185 | 3107530 | CC-NB-LRR |
| PCE_A_Chro1G0042200.2 | PCE_Avium_Chro1 | 3104185 | 3107530 | CC-NB-LRR |
| PCE_A_Chro1G0042400.1 | PCE_Avium_Chro1 | 3114442 | 3117812 | CC-NB-LRR |
| PCE_A_Chro1G0042400.2 | PCE_Avium_Chro1 | 3114442 | 3117812 | CC-NB-LRR |
| PCE_A_Chro1G0048000.1 | PCE_Avium_Chro1 | 3523810 | 3529546 | RLK |
| PCE_A_Chro1G0048000.2 | PCE_Avium_Chro1 | 3523810 | 3529546 | RLK |
| PCE_A_Chro1G0049600.1 | PCE_Avium_Chro1 | 3612008 | 3616460 | RLK |
| PCE_A_Chro1G0049600.2 | PCE_Avium_Chro1 | 3612008 | 3616460 | RLK |
| PCE_A_Chro1G0063300.1 | PCE_Avium_Chro1 | 4892320 | 4896197 | RLK |
| PCE_A_Chro1G0063300.2 | PCE_Avium_Chro1 | 4892320 | 4896197 | RLK |
| PCE_A_Chro1G0085100.1 | PCE_Avium_Chro1 | 7529660 | 7534608 | RLK |
| PCE_A_Chro1G0085100.3 | PCE_Avium_Chro1 | 7529660 | 7534608 | RLK |
| PCE_A_Chro1G0085100.4 | PCE_Avium_Chro1 | 7530537 | 7534608 | RLK |
| PCE_A_Chro1G0124400.1 | PCE_Avium_Chro1 | 11044575 | 11046621 | RLP |
| PCE_A_Chro1G0124500.1 | PCE_Avium_Chro1 | 11048554 | 11051714 | RLK |
| PCE_A_Chro1G0150400.1 | PCE_Avium_Chro1 | 13459638 | 13466173 | CC-NB-LRR |
| PCE_A_Chro1G0150400.2 | PCE_Avium_Chro1 | 13459638 | 13466173 | CC-NB-LRR |
| PCE_A_Chro1G0155400.1 | PCE_Avium_Chro1 | 14155573 | 14159200 | RLK |
| PCE_A_Chro1G0155500.1 | PCE_Avium_Chro1 | 14162194 | 14165738 | RLK |
| PCE_A_Chro1G0168000.1 | PCE_Avium_Chro1 | 15517487 | 15521415 | RLK |
| PCE_A_Chro1G0168000.2 | PCE_Avium_Chro1 | 15517487 | 15521415 | RLK |
| PCE_A_Chro1G0168000.3 | PCE_Avium_Chro1 | 15517487 | 15521415 | RLK |
| PCE_A_Chro1G0180700.2 | PCE_Avium_Chro1 | 17118911 | 17121497 | SPTMK |
| PCE_A_Chro1G0180700.5 | PCE_Avium_Chro1 | 17118911 | 17121497 | SPTMK |
| PCE_A_Chro1G0180700.1 | PCE_Avium_Chro1 | 17118938 | 17121497 | SPTMK |
| PCE_A_Chro1G0180700.3 | PCE_Avium_Chro1 | 17118938 | 17121497 | SPTMK |
| PCE_A_Chro1G0180700.6 | PCE_Avium_Chro1 | 17118938 | 17121497 | SPTMK |
| PCE_A_Chro1G0181000.2 | PCE_Avium_Chro1 | 17190095 | 17204853 | SPTMK |
| PCE_A_Chro1G0181000.1 | PCE_Avium_Chro1 | 17190122 | 17204853 | SPTMK |
| PCE_A_Chro1G0181000.3 | PCE_Avium_Chro1 | 17190122 | 17204853 | SPTMK |
| PCE_A_Chro1G0181000.5 | PCE_Avium_Chro1 | 17190122 | 17204853 | SPTMK |
| PCE_A_Chro1G0181300.1 | PCE_Avium_Chro1 | 17256292 | 17259898 | SPTMK |
| PCE_A_Chro1G0181300.3 | PCE_Avium_Chro1 | 17256292 | 17259898 | SPTMK |
| PCE_A_Chro1G0181300.4 | PCE_Avium_Chro1 | 17256292 | 17259898 | SPTMK |
| PCE_A_Chro1G0181300.5 | PCE_Avium_Chro1 | 17256292 | 17259898 | SPTMK |
| PCE_A_Chro1G0181500.3 | PCE_Avium_Chro1 | 17268238 | 17271606 | SPTMK |
| PCE_A_Chro1G0181500.4 | PCE_Avium_Chro1 | 17268238 | 17271606 | SPTMK |
| PCE_A_Chro1G0181500.1 | PCE_Avium_Chro1 | 17268265 | 17271606 | SPTMK |
| PCE_A_Chro1G0181500.2 | PCE_Avium_Chro1 | 17268265 | 17271606 | SPTMK |
| PCE_A_Chro1G0181500.5 | PCE_Avium_Chro1 | 17268265 | 17271606 | SPTMK |
| PCE_A_Chro1G0181500.7 | PCE_Avium_Chro1 | 17268265 | 17271606 | SPTMK |
| PCE_A_Chro1G0187300.1 | PCE_Avium_Chro1 | 18127986 | 18131654 | RLK |
| PCE_A_Chro1G0190900.1 | PCE_Avium_Chro1 | 18656171 | 18662452 | CC-NB-LRR |
| PCE_A_Chro1G0226000.1 | PCE_Avium_Chro1 | 27383718 | 27387281 | SPTMK |
| PCE_A_Chro1G0226000.2 | PCE_Avium_Chro1 | 27383718 | 27387281 | SPTMK |
| PCE_A_Chro1G0236100.1 | PCE_Avium_Chro1 | 28535446 | 28539688 | RLK |
| PCE_A_Chro1G0236100.3 | PCE_Avium_Chro1 | 28535446 | 28539688 | RLK |
| PCE_A_Chro1G0246800.1 | PCE_Avium_Chro1 | 29605852 | 29611912 | RLK |
| PCE_A_Chro1G0246800.2 | PCE_Avium_Chro1 | 29605852 | 29611912 | RLK |
| PCE_A_Chro1G0246800.5 | PCE_Avium_Chro1 | 29605852 | 29611912 | RLK |
| PCE_A_Chro1G0256200.1 | PCE_Avium_Chro1 | 30404041 | 30407395 | LysM |
| PCE_A_Chro1G0257600.1 | PCE_Avium_Chro1 | 30516280 | 30519280 | SPTMK |
| PCE_A_Chro1G0257600.2 | PCE_Avium_Chro1 | 30516280 | 30518300 | SPTMK |
| PCE_A_Chro1G0257600.3 | PCE_Avium_Chro1 | 30516280 | 30518956 | SPTMK |
| PCE_A_Chro1G0257700.1 | PCE_Avium_Chro1 | 30520181 | 30524435 | SPTMK |
| PCE_A_Chro1G0257700.2 | PCE_Avium_Chro1 | 30520181 | 30523382 | SPTMK |
| PCE_A_Chro1G0257800.1 | PCE_Avium_Chro1 | 30548002 | 30552607 | SPTMK |
| PCE_A_Chro1G0257800.2 | PCE_Avium_Chro1 | 30548002 | 30551012 | SPTMK |
| PCE_A_Chro1G0258400.10 | PCE_Avium_Chro1 | 30602049 | 30607635 | RLK |
| PCE_A_Chro1G0258400.12 | PCE_Avium_Chro1 | 30602049 | 30607635 | RLK |
| PCE_A_Chro1G0258400.13 | PCE_Avium_Chro1 | 30602049 | 30607635 | RLK |
| PCE_A_Chro1G0258400.14 | PCE_Avium_Chro1 | 30602049 | 30607635 | RLK |
| PCE_A_Chro1G0258400.15 | PCE_Avium_Chro1 | 30602049 | 30607635 | RLK |
| PCE_A_Chro1G0258400.16 | PCE_Avium_Chro1 | 30602049 | 30607635 | RLK |
| PCE_A_Chro1G0258400.17 | PCE_Avium_Chro1 | 30602049 | 30607635 | RLK |
| PCE_A_Chro1G0258400.18 | PCE_Avium_Chro1 | 30602049 | 30607635 | RLK |
| PCE_A_Chro1G0258400.1 | PCE_Avium_Chro1 | 30602049 | 30607635 | RLK |
| PCE_A_Chro1G0258400.2 | PCE_Avium_Chro1 | 30602049 | 30607635 | RLK |
| PCE_A_Chro1G0258400.4 | PCE_Avium_Chro1 | 30602049 | 30607635 | RLK |
| PCE_A_Chro1G0258400.5 | PCE_Avium_Chro1 | 30602049 | 30607635 | RLK |
| PCE_A_Chro1G0258400.6 | PCE_Avium_Chro1 | 30602049 | 30607635 | RLK |
| PCE_A_Chro1G0258400.9 | PCE_Avium_Chro1 | 30602049 | 30607635 | RLK |
| PCE_A_Chro1G0261100.5 | PCE_Avium_Chro1 | 30743319 | 30746602 | SPTMK |
| PCE_A_Chro1G0263300.1 | PCE_Avium_Chro1 | 30892184 | 30894816 | SPTMK |
| PCE_A_Chro1G0263300.2 | PCE_Avium_Chro1 | 30892184 | 30894816 | SPTMK |
| PCE_A_Chro1G0271600.1 | PCE_Avium_Chro1 | 31587217 | 31589450 | RLK |
| PCE_A_Chro1G0271600.2 | PCE_Avium_Chro1 | 31587217 | 31589450 | RLK |
| PCE_A_Chro1G0272300.1 | PCE_Avium_Chro1 | 31687199 | 31693121 | TIR-NB-LRR |
| PCE_A_Chro1G0272300.4 | PCE_Avium_Chro1 | 31687199 | 31693121 | TIR-NB-LRR |
| PCE_A_Chro1G0273900.1 | PCE_Avium_Chro1 | 31897541 | 31899948 | SPTMK |
| PCE_A_Chro1G0278300.3 | PCE_Avium_Chro1 | 32274204 | 32279828 | RLK |
| PCE_A_Chro1G0278300.1 | PCE_Avium_Chro1 | 32274204 | 32279828 | RLK |
| PCE_A_Chro1G0278300.2 | PCE_Avium_Chro1 | 32274204 | 32279828 | RLK |
| PCE_A_Chro1G0289600.1 | PCE_Avium_Chro1 | 33216969 | 33220959 | RLK |
| PCE_A_Chro1G0301700.1 | PCE_Avium_Chro1 | 33906536 | 33910811 | RLK |
| PCE_A_Chro1G0301700.3 | PCE_Avium_Chro1 | 33906536 | 33910811 | RLK |
| PCE_A_Chro1G0305700.1 | PCE_Avium_Chro1 | 34182764 | 34185123 | RLP |
| PCE_A_Chro1G0307900.1 | PCE_Avium_Chro1 | 34317966 | 34323141 | RLP |
| PCE_A_Chro1G0307900.2 | PCE_Avium_Chro1 | 34317966 | 34323141 | RLP |
| PCE_A_Chro1G0316400.1 | PCE_Avium_Chro1 | 34830232 | 34833174 | RLK |
| PCE_A_Chro1G0316500.1 | PCE_Avium_Chro1 | 34836267 | 34839716 | SPTMK |
| PCE_A_Chro1G0317100.1 | PCE_Avium_Chro1 | 34860919 | 34863837 | SPTMK |
| PCE_A_Chro1G0317100.2 | PCE_Avium_Chro1 | 34860919 | 34863837 | SPTMK |
| PCE_A_Chro1G0319400.1 | PCE_Avium_Chro1 | 34986023 | 34988664 | RLK |
| PCE_A_Chro1G0320500.1 | PCE_Avium_Chro1 | 35036686 | 35040019 | SPTMK |
| PCE_A_Chro1G0320500.2 | PCE_Avium_Chro1 | 35036686 | 35043658 | SPTMK |
| PCE_A_Chro1G0321500.1 | PCE_Avium_Chro1 | 35098525 | 35101435 | RLK |
| PCE_A_Chro1G0339400.1 | PCE_Avium_Chro1 | 36383490 | 36386309 | SPTMK |
| PCE_A_Chro1G0339700.1 | PCE_Avium_Chro1 | 36398752 | 36403290 | RLK |
| PCE_A_Chro1G0342100.1 | PCE_Avium_Chro1 | 36571369 | 36575448 | RLK |
| PCE_A_Chro1G0346500.1 | PCE_Avium_Chro1 | 36892855 | 36896717 | RLK |
| PCE_A_Chro1G0350300.1 | PCE_Avium_Chro1 | 37209337 | 37212702 | LysM |
| PCE_A_Chro1G0350300.2 | PCE_Avium_Chro1 | 37209337 | 37212702 | LysM |
| PCE_A_Chro1G0350300.3 | PCE_Avium_Chro1 | 37209337 | 37212702 | LysM |
| PCE_A_Chro1G0365800.1 | PCE_Avium_Chro1 | 38166961 | 38170650 | RLK |
| PCE_A_Chro1G0372600.1 | PCE_Avium_Chro1 | 38625679 | 38629408 | RLK |
| PCE_A_Chro1G0381600.1 | PCE_Avium_Chro1 | 39137192 | 39141674 | RLK |
| PCE_A_Chro1G0381600.2 | PCE_Avium_Chro1 | 39137192 | 39141674 | RLK |
| PCE_A_Chro1G0381600.3 | PCE_Avium_Chro1 | 39137192 | 39141674 | RLK |
| PCE_A_Chro1G0387800.1 | PCE_Avium_Chro1 | 39553730 | 39558345 | SPTMK |
| PCE_A_Chro1G0390500.1 | PCE_Avium_Chro1 | 39763280 | 39766560 | RPW8-NB-LRR |
| PCE_A_Chro1G0398100.1 | PCE_Avium_Chro1 | 40343160 | 40351436 | RLK |
| PCE_A_Chro1G0398100.2 | PCE_Avium_Chro1 | 40343160 | 40351436 | RLK |
| PCE_A_Chro1G0398100.3 | PCE_Avium_Chro1 | 40343160 | 40351436 | RLK |
| PCE_A_Chro1G0407200.1 | PCE_Avium_Chro1 | 40834238 | 40836616 | RLK |
| PCE_A_Chro1G0422600.1 | PCE_Avium_Chro1 | 41720301 | 41723797 | RLK |
| PCE_A_Chro1G0422700.2 | PCE_Avium_Chro1 | 41726557 | 41729731 | RLK |
| PCE_A_Chro1G0422700.1 | PCE_Avium_Chro1 | 41726557 | 41729731 | RLK |
| PCE_A_Chro1G0424100.1 | PCE_Avium_Chro1 | 41800704 | 41803432 | SPTMK |
| PCE_A_Chro1G0424200.1 | PCE_Avium_Chro1 | 41803938 | 41806803 | SPTMK |
| PCE_A_Chro1G0424300.1 | PCE_Avium_Chro1 | 41814454 | 41816829 | SPTMK |
| PCE_A_Chro1G0424400.1 | PCE_Avium_Chro1 | 41829501 | 41831930 | SPTMK |
| PCE_A_Chro1G0424500.1 | PCE_Avium_Chro1 | 41834309 | 41836723 | SPTMK |
| PCE_A_Chro1G0424600.1 | PCE_Avium_Chro1 | 41840533 | 41842938 | SPTMK |
| PCE_A_Chro1G0426000.1 | PCE_Avium_Chro1 | 41923258 | 41926780 | RLK |
| PCE_A_Chro1G0433000.1 | PCE_Avium_Chro1 | 42326746 | 42331121 | RLK |
| PCE_A_Chro1G0441200.1 | PCE_Avium_Chro1 | 42868884 | 42872616 | RLK |
| PCE_A_Chro1G0441200.2 | PCE_Avium_Chro1 | 42868884 | 42872616 | RLK |
| PCE_A_Chro1G0441200.3 | PCE_Avium_Chro1 | 42868884 | 42872616 | RLK |
| PCE_A_Chro1G0441200.4 | PCE_Avium_Chro1 | 42868884 | 42872616 | RLK |
| PCE_A_Chro1G0441200.7 | PCE_Avium_Chro1 | 42868884 | 42872616 | RLK |
| PCE_A_Chro1G0441200.8 | PCE_Avium_Chro1 | 42868884 | 42872616 | RLK |
| PCE_A_Chro1G0441200.5 | PCE_Avium_Chro1 | 42868884 | 42872616 | RLK |
| PCE_A_Chro1G0450500.1 | PCE_Avium_Chro1 | 43440753 | 43443471 | RLK |
| PCE_A_Chro1G0458500.1 | PCE_Avium_Chro1 | 44104212 | 44106955 | SPTMK |
| PCE_A_Chro1G0459100.1 | PCE_Avium_Chro1 | 44174604 | 44177629 | SPTMK |
| PCE_A_Chro1G0459100.2 | PCE_Avium_Chro1 | 44174604 | 44177629 | SPTMK |
| PCE_A_Chro1G0459300.1 | PCE_Avium_Chro1 | 44199661 | 44202678 | SPTMK |
| PCE_A_Chro1G0459300.2 | PCE_Avium_Chro1 | 44199661 | 44202678 | SPTMK |
| PCE_A_Chro1G0459300.3 | PCE_Avium_Chro1 | 44199661 | 44201860 | SPTMK |
| PCE_A_Chro1G0466300.1 | PCE_Avium_Chro1 | 44634856 | 44639248 | SPTMK |
| PCE_A_Chro1G0470300.1 | PCE_Avium_Chro1 | 44928892 | 44931349 | SPTMK |
| PCE_A_Chro1G0471500.1 | PCE_Avium_Chro1 | 45011673 | 45015447 | SPTMK |
| PCE_A_Chro1G0472100.1 | PCE_Avium_Chro1 | 45042879 | 45047154 | RLP |
| PCE_A_Chro1G0477700.1 | PCE_Avium_Chro1 | 45396028 | 45399813 | SPTMK |
| PCE_A_Chro1G0483800.1 | PCE_Avium_Chro1 | 45862614 | 45865797 | LysM |
| PCE_A_Chro1G0483800.2 | PCE_Avium_Chro1 | 45862614 | 45865797 | LysM |
| PCE_A_Chro1G0483800.3 | PCE_Avium_Chro1 | 45862614 | 45865797 | LysM |
| PCE_A_Chro1G0494300.1 | PCE_Avium_Chro1 | 46622196 | 46625728 | RLK |
| PCE_A_Chro1G0495800.1 | PCE_Avium_Chro1 | 46752577 | 46755894 | RLK |
| PCE_A_Chro1G0496100.2 | PCE_Avium_Chro1 | 46767204 | 46770747 | RLK |
| PCE_A_Chro1G0496200.1 | PCE_Avium_Chro1 | 46771283 | 46774486 | RLK |
| PCE_A_Chro1G0504300.1 | PCE_Avium_Chro1 | 47335204 | 47344826 | CC-NB-LRR |
| PCE_A_Chro1G0504500.1 | PCE_Avium_Chro1 | 47349978 | 47359047 | CC-NB-LRR |
| PCE_A_Chro1G0504800.1 | PCE_Avium_Chro1 | 47373355 | 47376270 | CC-NB-LRR |
| PCE_A_Chro1G0508500.1 | PCE_Avium_Chro1 | 47683229 | 47686075 | CC-NB-LRR |
| PCE_A_Chro1G0508600.1 | PCE_Avium_Chro1 | 47693351 | 47698519 | CC-NB-LRR |
| PCE_A_Chro1G0518700.1 | PCE_Avium_Chro1 | 48494531 | 48497996 | RLP |
| PCE_A_Chro1G0521400.2 | PCE_Avium_Chro1 | 48734064 | 48738827 | RLK |
| PCE_A_Chro1G0521400.1 | PCE_Avium_Chro1 | 48734064 | 48738827 | RLK |
| PCE_A_Chro1G0521400.3 | PCE_Avium_Chro1 | 48734064 | 48738827 | RLK |
| PCE_A_Chro1G0522400.1 | PCE_Avium_Chro1 | 48852506 | 48856396 | RLK |
| PCE_A_Chro1G0522500.1 | PCE_Avium_Chro1 | 48863446 | 48867807 | RLK |
| PCE_A_Chro1G0524900.1 | PCE_Avium_Chro1 | 49106011 | 49108921 | CC-NB-LRR |
| PCE_A_Chro1G0525000.1 | PCE_Avium_Chro1 | 49119284 | 49122129 | CC-NB-LRR |
| PCE_A_Chro1G0525200.1 | PCE_Avium_Chro1 | 49137929 | 49140763 | CC-NB-LRR |
| PCE_A_Chro1G0526800.1 | PCE_Avium_Chro1 | 49324143 | 49326965 | CC-NB-LRR |
| PCE_A_Chro1G0526900.1 | PCE_Avium_Chro1 | 49328525 | 49331329 | CC-NB-LRR |
| PCE_A_Chro1G0530100.1 | PCE_Avium_Chro1 | 49592067 | 49595772 | TIR-NB-LRR |
| PCE_A_Chro1G0539400.1 | PCE_Avium_Chro1 | 50426689 | 50432785 | RLK |
| PCE_A_Chro1G0539400.2 | PCE_Avium_Chro1 | 50426689 | 50432785 | RLK |
| PCE_A_Chro1G0539400.4 | PCE_Avium_Chro1 | 50426689 | 50432785 | RLK |
| PCE_A_Chro1G0539400.5 | PCE_Avium_Chro1 | 50426689 | 50432785 | RLK |
| PCE_A_Chro1G0539400.6 | PCE_Avium_Chro1 | 50426689 | 50432785 | RLK |
| PCE_A_Chro1G0539400.3 | PCE_Avium_Chro1 | 50426689 | 50432785 | RLK |
| PCE_A_Chro1G0551200.1 | PCE_Avium_Chro1 | 51404789 | 51408058 | SPTMK |
| PCE_A_Chro1G0556100.1 | PCE_Avium_Chro1 | 51833097 | 51836077 | RLP |
| PCE_A_Chro1G0556100.2 | PCE_Avium_Chro1 | 51833097 | 51836077 | RLP |
| PCE_A_Chro1G0562900.1 | PCE_Avium_Chro1 | 52457571 | 52460504 | CC-NB-LRR |
| PCE_A_Chro1G0563200.1 | PCE_Avium_Chro1 | 52487394 | 52490306 | CC-NB-LRR |
| PCE_A_Chro1G0567300.1 | PCE_Avium_Chro1 | 52774137 | 52779568 | RLK |
| PCE_A_Chro2G0002300.1 | PCE_Avium_Chro2 | 197412 | 199677 | RLP |
| PCE_A_Chro2G0026900.1 | PCE_Avium_Chro2 | 1598060 | 1600984 | RLP |
| PCE_A_Chro2G0027200.1 | PCE_Avium_Chro2 | 1610852 | 1614289 | SPTMK |
| PCE_A_Chro2G0031500.1 | PCE_Avium_Chro2 | 1880150 | 1883451 | SPTMK |
| PCE_A_Chro2G0039300.1 | PCE_Avium_Chro2 | 2265542 | 2270255 | RLK |
| PCE_A_Chro2G0047000.1 | PCE_Avium_Chro2 | 2724915 | 2727950 | CC-NB-LRR |
| PCE_A_Chro2G0048800.1 | PCE_Avium_Chro2 | 2835060 | 2838896 | RLK |
| PCE_A_Chro2G0048800.2 | PCE_Avium_Chro2 | 2835060 | 2838896 | RLK |
| PCE_A_Chro2G0054400.1 | PCE_Avium_Chro2 | 3107331 | 3111733 | RLK |
| PCE_A_Chro2G0055100.1 | PCE_Avium_Chro2 | 3140803 | 3144180 | CC-NB-LRR |
| PCE_A_Chro2G0061000.1 | PCE_Avium_Chro2 | 3498765 | 3501902 | RLP |
| PCE_A_Chro2G0061000.2 | PCE_Avium_Chro2 | 3498765 | 3501914 | RLP |
| PCE_A_Chro2G0066700.1 | PCE_Avium_Chro2 | 3836620 | 3839287 | RLK |
| PCE_A_Chro2G0069500.1 | PCE_Avium_Chro2 | 3997269 | 4001878 | RLK |
| PCE_A_Chro2G0069500.2 | PCE_Avium_Chro2 | 3997269 | 4001878 | RLK |
| PCE_A_Chro2G0076000.1 | PCE_Avium_Chro2 | 4360680 | 4364143 | RLK |
| PCE_A_Chro2G0079000.1 | PCE_Avium_Chro2 | 4523366 | 4527624 | RLK |
| PCE_A_Chro2G0082500.1 | PCE_Avium_Chro2 | 4762474 | 4765134 | SPTMK |
| PCE_A_Chro2G0084400.1 | PCE_Avium_Chro2 | 4872628 | 4876912 | RLK |
| PCE_A_Chro2G0093100.1 | PCE_Avium_Chro2 | 5434714 | 5437401 | SPTMK |
| PCE_A_Chro2G0093100.2 | PCE_Avium_Chro2 | 5434714 | 5437401 | SPTMK |
| PCE_A_Chro2G0096200.1 | PCE_Avium_Chro2 | 5609266 | 5611973 | SPTMK |
| PCE_A_Chro2G0110900.1 | PCE_Avium_Chro2 | 6489005 | 6492484 | SPTMK |
| PCE_A_Chro2G0135200.1 | PCE_Avium_Chro2 | 8343075 | 8345846 | SPTMK |
| PCE_A_Chro2G0162200.1 | PCE_Avium_Chro2 | 10410057 | 10414320 | SPTMK |
| PCE_A_Chro2G0162200.2 | PCE_Avium_Chro2 | 10410057 | 10414320 | SPTMK |
| PCE_A_Chro2G0163200.1 | PCE_Avium_Chro2 | 10518935 | 10524585 | RLP |
| PCE_A_Chro2G0166400.1 | PCE_Avium_Chro2 | 10749823 | 10752635 | RLP |
| PCE_A_Chro2G0166700.1 | PCE_Avium_Chro2 | 10769430 | 10772516 | RLP |
| PCE_A_Chro2G0167000.1 | PCE_Avium_Chro2 | 10784221 | 10787741 | RLP |
| PCE_A_Chro2G0168800.1 | PCE_Avium_Chro2 | 10956487 | 10959977 | RLP |
| PCE_A_Chro2G0177600.1 | PCE_Avium_Chro2 | 11422875 | 11425807 | RLP |
| PCE_A_Chro2G0181100.1 | PCE_Avium_Chro2 | 11730445 | 11733135 | RLP |
| PCE_A_Chro2G0181400.2 | PCE_Avium_Chro2 | 11760913 | 11763789 | RLP |
| PCE_A_Chro2G0181400.1 | PCE_Avium_Chro2 | 11760913 | 11763789 | RLP |
| PCE_A_Chro2G0181700.1 | PCE_Avium_Chro2 | 11787961 | 11791205 | RLP |
| PCE_A_Chro2G0193700.1 | PCE_Avium_Chro2 | 12623716 | 12627743 | RLK |
| PCE_A_Chro2G0193900.2 | PCE_Avium_Chro2 | 12644565 | 12648644 | CC-NB-LRR |
| PCE_A_Chro2G0204800.1 | PCE_Avium_Chro2 | 13578497 | 13583475 | TIR-NB-LRR |
| PCE_A_Chro2G0204800.3 | PCE_Avium_Chro2 | 13578497 | 13583475 | TIR-NB-LRR |
| PCE_A_Chro2G0204800.2 | PCE_Avium_Chro2 | 13578497 | 13582815 | TIR-NB-LRR |
| PCE_A_Chro2G0204900.1 | PCE_Avium_Chro2 | 13584611 | 13588764 | TIR-NB-LRR |
| PCE_A_Chro2G0205100.1 | PCE_Avium_Chro2 | 13655527 | 13663508 | TIR-NB-LRR |
| PCE_A_Chro2G0205100.2 | PCE_Avium_Chro2 | 13655527 | 13663239 | TIR-NB-LRR |
| PCE_A_Chro2G0210700.1 | PCE_Avium_Chro2 | 14206992 | 14210055 | RLP |
| PCE_A_Chro2G0210900.1 | PCE_Avium_Chro2 | 14226719 | 14229580 | RLP |
| PCE_A_Chro2G0211000.1 | PCE_Avium_Chro2 | 14239554 | 14242854 | RLP |
| PCE_A_Chro2G0211200.1 | PCE_Avium_Chro2 | 14283232 | 14286113 | RLP |
| PCE_A_Chro2G0211300.1 | PCE_Avium_Chro2 | 14297743 | 14300562 | RLP |
| PCE_A_Chro2G0211500.1 | PCE_Avium_Chro2 | 14373558 | 14377142 | RLP |
| PCE_A_Chro2G0211600.1 | PCE_Avium_Chro2 | 14401057 | 14407054 | RLP |
| PCE_A_Chro2G0213400.1 | PCE_Avium_Chro2 | 14588632 | 14591439 | RLP |
| PCE_A_Chro2G0213500.1 | PCE_Avium_Chro2 | 14614082 | 14617009 | RLP |
| PCE_A_Chro2G0213900.1 | PCE_Avium_Chro2 | 14633626 | 14636871 | RLP |
| PCE_A_Chro2G0214100.1 | PCE_Avium_Chro2 | 14659236 | 14662515 | RLP |
| PCE_A_Chro2G0216100.1 | PCE_Avium_Chro2 | 14824246 | 14829643 | TIR-NB-LRR |
| PCE_A_Chro2G0216100.2 | PCE_Avium_Chro2 | 14824246 | 14829643 | TIR-NB-LRR |
| PCE_A_Chro2G0216200.4 | PCE_Avium_Chro2 | 14833192 | 14837683 | TIR-NB-LRR |
| PCE_A_Chro2G0216200.2 | PCE_Avium_Chro2 | 14833192 | 14837683 | TIR-NB-LRR |
| PCE_A_Chro2G0216200.1 | PCE_Avium_Chro2 | 14833574 | 14837683 | TIR-NB-LRR |
| PCE_A_Chro2G0216200.3 | PCE_Avium_Chro2 | 14833887 | 14837683 | TIR-NB-LRR |
| PCE_A_Chro2G0216600.1 | PCE_Avium_Chro2 | 14870876 | 14875401 | TIR-NB-LRR |
| PCE_A_Chro2G0216600.2 | PCE_Avium_Chro2 | 14871381 | 14875401 | TIR-NB-LRR |
| PCE_A_Chro2G0216600.3 | PCE_Avium_Chro2 | 14871996 | 14875401 | TIR-NB-LRR |
| PCE_A_Chro2G0216800.1 | PCE_Avium_Chro2 | 14886225 | 14892017 | TIR-NB-LRR |
| PCE_A_Chro2G0216900.1 | PCE_Avium_Chro2 | 14905252 | 14909926 | TIR-NB-LRR |
| PCE_A_Chro2G0216900.4 | PCE_Avium_Chro2 | 14905252 | 14909926 | TIR-NB-LRR |
| PCE_A_Chro2G0216900.3 | PCE_Avium_Chro2 | 14905252 | 14909926 | TIR-NB-LRR |
| PCE_A_Chro2G0216900.2 | PCE_Avium_Chro2 | 14905889 | 14909926 | TIR-NB-LRR |
| PCE_A_Chro2G0217200.1 | PCE_Avium_Chro2 | 14929984 | 14934500 | TIR-NB-LRR |
| PCE_A_Chro2G0217700.1 | PCE_Avium_Chro2 | 14992882 | 15005621 | TIR-NB-LRR |
| PCE_A_Chro2G0217700.2 | PCE_Avium_Chro2 | 15000928 | 15005621 | TIR-NB-LRR |
| PCE_A_Chro2G0218100.1 | PCE_Avium_Chro2 | 15027389 | 15032154 | TIR-NB-LRR |
| PCE_A_Chro2G0218100.4 | PCE_Avium_Chro2 | 15027389 | 15032154 | TIR-NB-LRR |
| PCE_A_Chro2G0218100.3 | PCE_Avium_Chro2 | 15027389 | 15032154 | TIR-NB-LRR |
| PCE_A_Chro2G0218100.2 | PCE_Avium_Chro2 | 15027921 | 15032154 | TIR-NB-LRR |
| PCE_A_Chro2G0226300.1 | PCE_Avium_Chro2 | 15900465 | 15906909 | CC-NB-LRR |
| PCE_A_Chro2G0226700.3 | PCE_Avium_Chro2 | 15948655 | 15953364 | CC-NB-LRR |
| PCE_A_Chro2G0226700.1 | PCE_Avium_Chro2 | 15948655 | 15955258 | CC-NB-LRR |
| PCE_A_Chro2G0226700.2 | PCE_Avium_Chro2 | 15948655 | 15955258 | CC-NB-LRR |
| PCE_A_Chro2G0226800.1 | PCE_Avium_Chro2 | 15963010 | 15973024 | CC-NB-LRR |
| PCE_A_Chro2G0226800.2 | PCE_Avium_Chro2 | 15963010 | 15972681 | CC-NB-LRR |
| PCE_A_Chro2G0227900.2 | PCE_Avium_Chro2 | 16026492 | 16029637 | RLK |
| PCE_A_Chro2G0228300.1 | PCE_Avium_Chro2 | 16084075 | 16087259 | RLK |
| PCE_A_Chro2G0228700.1 | PCE_Avium_Chro2 | 16144286 | 16149689 | RLK |
| PCE_A_Chro2G0241100.1 | PCE_Avium_Chro2 | 17669411 | 17671431 | SPTMK |
| PCE_A_Chro2G0241400.1 | PCE_Avium_Chro2 | 17690299 | 17706712 | SPTMK |
| PCE_A_Chro2G0241400.2 | PCE_Avium_Chro2 | 17690299 | 17706712 | SPTMK |
| PCE_A_Chro2G0241600.2 | PCE_Avium_Chro2 | 17767031 | 17769601 | SPTMK |
| PCE_A_Chro2G0243100.1 | PCE_Avium_Chro2 | 17919423 | 17924117 | TIR-NB-LRR |
| PCE_A_Chro2G0244600.1 | PCE_Avium_Chro2 | 18218850 | 18222095 | CC-NB-LRR |
| PCE_A_Chro2G0245900.1 | PCE_Avium_Chro2 | 18454526 | 18459856 | RLP |
| PCE_A_Chro2G0246000.1 | PCE_Avium_Chro2 | 18468250 | 18470478 | RLP |
| PCE_A_Chro2G0246100.1 | PCE_Avium_Chro2 | 18491913 | 18494141 | RLP |
| PCE_A_Chro2G0247400.1 | PCE_Avium_Chro2 | 18675953 | 18678181 | RLP |
| PCE_A_Chro2G0247500.1 | PCE_Avium_Chro2 | 18682932 | 18685265 | RLP |
| PCE_A_Chro2G0247600.1 | PCE_Avium_Chro2 | 18699717 | 18703685 | CC-NB-LRR |
| PCE_A_Chro2G0247700.1 | PCE_Avium_Chro2 | 18710961 | 18713189 | RLP |
| PCE_A_Chro2G0251900.1 | PCE_Avium_Chro2 | 19201982 | 19204607 | SPTMK |
| PCE_A_Chro2G0251900.2 | PCE_Avium_Chro2 | 19201982 | 19204607 | SPTMK |
| PCE_A_Chro2G0252100.1 | PCE_Avium_Chro2 | 19223214 | 19225813 | SPTMK |
| PCE_A_Chro2G0252100.2 | PCE_Avium_Chro2 | 19223214 | 19225813 | SPTMK |
| PCE_A_Chro2G0255800.3 | PCE_Avium_Chro2 | 19818262 | 19820448 | SPTMK |
| PCE_A_Chro2G0255800.4 | PCE_Avium_Chro2 | 19818262 | 19820448 | SPTMK |
| PCE_A_Chro2G0255800.1 | PCE_Avium_Chro2 | 19818283 | 19820448 | SPTMK |
| PCE_A_Chro2G0255800.2 | PCE_Avium_Chro2 | 19818283 | 19820448 | SPTMK |
| PCE_A_Chro2G0256400.1 | PCE_Avium_Chro2 | 19942135 | 19944878 | SPTMK |
| PCE_A_Chro2G0256400.2 | PCE_Avium_Chro2 | 19942135 | 19944878 | SPTMK |
| PCE_A_Chro2G0256700.1 | PCE_Avium_Chro2 | 19960402 | 19962829 | SPTMK |
| PCE_A_Chro2G0256700.2 | PCE_Avium_Chro2 | 19960402 | 19962829 | SPTMK |
| PCE_A_Chro2G0256700.3 | PCE_Avium_Chro2 | 19960402 | 19962859 | SPTMK |
| PCE_A_Chro2G0256700.4 | PCE_Avium_Chro2 | 19960402 | 19962829 | SPTMK |
| PCE_A_Chro2G0256800.1 | PCE_Avium_Chro2 | 19963682 | 19966312 | SPTMK |
| PCE_A_Chro2G0256800.2 | PCE_Avium_Chro2 | 19963682 | 19966312 | SPTMK |
| PCE_A_Chro2G0256800.3 | PCE_Avium_Chro2 | 19963682 | 19966312 | SPTMK |
| PCE_A_Chro2G0257000.1 | PCE_Avium_Chro2 | 20008855 | 20011547 | SPTMK |
| PCE_A_Chro2G0257000.2 | PCE_Avium_Chro2 | 20008855 | 20011547 | SPTMK |
| PCE_A_Chro2G0257400.1 | PCE_Avium_Chro2 | 20106400 | 20108608 | SPTMK |
| PCE_A_Chro2G0257400.2 | PCE_Avium_Chro2 | 20106400 | 20108608 | SPTMK |
| PCE_A_Chro2G0257400.3 | PCE_Avium_Chro2 | 20106400 | 20108608 | SPTMK |
| PCE_A_Chro2G0258000.1 | PCE_Avium_Chro2 | 20264584 | 20266673 | SPTMK |
| PCE_A_Chro2G0258200.1 | PCE_Avium_Chro2 | 20301661 | 20303737 | SPTMK |
| PCE_A_Chro2G0258200.2 | PCE_Avium_Chro2 | 20301661 | 20303737 | SPTMK |
| PCE_A_Chro2G0269300.1 | PCE_Avium_Chro2 | 22287799 | 22291216 | RLP |
| PCE_A_Chro2G0270500.1 | PCE_Avium_Chro2 | 22384609 | 22388178 | RLP |
| PCE_A_Chro2G0273000.1 | PCE_Avium_Chro2 | 22701099 | 22704579 | RLK |
| PCE_A_Chro2G0273700.1 | PCE_Avium_Chro2 | 22790851 | 22793718 | RLP |
| PCE_A_Chro2G0286400.1 | PCE_Avium_Chro2 | 26591707 | 26598030 | RLK |
| PCE_A_Chro2G0286400.2 | PCE_Avium_Chro2 | 26591707 | 26598030 | RLK |
| PCE_A_Chro2G0286400.3 | PCE_Avium_Chro2 | 26591707 | 26598030 | RLK |
| PCE_A_Chro2G0286400.5 | PCE_Avium_Chro2 | 26591707 | 26598030 | RLK |
| PCE_A_Chro2G0286400.6 | PCE_Avium_Chro2 | 26591707 | 26598030 | RLK |
| PCE_A_Chro2G0292000.1 | PCE_Avium_Chro2 | 27783118 | 27786593 | CC-NB-LRR |
| PCE_A_Chro2G0293400.1 | PCE_Avium_Chro2 | 28023578 | 28029355 | TIR-NB-LRR |
| PCE_A_Chro2G0294700.1 | PCE_Avium_Chro2 | 28232445 | 28249496 | TIR-NB-LRR |
| PCE_A_Chro2G0294700.2 | PCE_Avium_Chro2 | 28232445 | 28255099 | TIR-NB-LRR |
| PCE_A_Chro2G0296700.2 | PCE_Avium_Chro2 | 28495580 | 28500829 | TIR-NB-LRR |
| PCE_A_Chro2G0296700.3 | PCE_Avium_Chro2 | 28495580 | 28500829 | TIR-NB-LRR |
| PCE_A_Chro2G0296700.4 | PCE_Avium_Chro2 | 28495580 | 28500829 | TIR-NB-LRR |
| PCE_A_Chro2G0296700.1 | PCE_Avium_Chro2 | 28496072 | 28500829 | TIR-NB-LRR |
| PCE_A_Chro2G0296700.5 | PCE_Avium_Chro2 | 28496072 | 28500829 | TIR-NB-LRR |
| PCE_A_Chro2G0298900.3 | PCE_Avium_Chro2 | 28810325 | 28815962 | TIR-NB-LRR |
| PCE_A_Chro2G0298900.1 | PCE_Avium_Chro2 | 28810325 | 28815962 | TIR-NB-LRR |
| PCE_A_Chro2G0298900.2 | PCE_Avium_Chro2 | 28810325 | 28815962 | TIR-NB-LRR |
| PCE_A_Chro2G0298900.4 | PCE_Avium_Chro2 | 28810325 | 28815962 | TIR-NB-LRR |
| PCE_A_Chro2G0298900.5 | PCE_Avium_Chro2 | 28810325 | 28815962 | TIR-NB-LRR |
| PCE_A_Chro2G0300100.1 | PCE_Avium_Chro2 | 29125217 | 29129161 | CC-NB-LRR |
| PCE_A_Chro2G0300300.1 | PCE_Avium_Chro2 | 29160624 | 29166851 | CC-NB-LRR |
| PCE_A_Chro2G0300500.1 | PCE_Avium_Chro2 | 29225778 | 29229852 | CC-NB-LRR |
| PCE_A_Chro2G0300600.1 | PCE_Avium_Chro2 | 29250491 | 29254273 | CC-NB-LRR |
| PCE_A_Chro2G0307300.1 | PCE_Avium_Chro2 | 30516939 | 30528790 | CC-NB-LRR |
| PCE_A_Chro2G0309000.1 | PCE_Avium_Chro2 | 30753783 | 30757762 | CC-NB-LRR |
| PCE_A_Chro2G0309100.1 | PCE_Avium_Chro2 | 30762642 | 30766508 | CC-NB-LRR |
| PCE_A_Chro2G0309200.1 | PCE_Avium_Chro2 | 30781980 | 30785970 | CC-NB-LRR |
| PCE_A_Chro2G0312600.1 | PCE_Avium_Chro2 | 31245301 | 31253964 | CC-NB-LRR |
| PCE_A_Chro2G0312700.1 | PCE_Avium_Chro2 | 31269791 | 31276842 | CC-NB-LRR |
| PCE_A_Chro2G0315800.1 | PCE_Avium_Chro2 | 31576568 | 31581062 | CC-NB-LRR |
| PCE_A_Chro2G0318900.1 | PCE_Avium_Chro2 | 32058204 | 32062553 | CC-NB-LRR |
| PCE_A_Chro2G0325800.1 | PCE_Avium_Chro2 | 33144098 | 33149720 | CC-NB-LRR |
| PCE_A_Chro2G0326300.1 | PCE_Avium_Chro2 | 33217991 | 33222126 | TIR-NB-LRR |
| PCE_A_Chro2G0330300.1 | PCE_Avium_Chro2 | 33868748 | 33873671 | CC-NB-LRR |
| PCE_A_Chro2G0335500.1 | PCE_Avium_Chro2 | 34621847 | 34627340 | CC-NB-LRR |
| PCE_A_Chro2G0335500.2 | PCE_Avium_Chro2 | 34621847 | 34627340 | CC-NB-LRR |
| PCE_A_Chro2G0336400.1 | PCE_Avium_Chro2 | 34744044 | 34749111 | CC-NB-LRR |
| PCE_A_Chro2G0337000.1 | PCE_Avium_Chro2 | 34827492 | 34833380 | CC-NB-LRR |
| PCE_A_Chro2G0338000.1 | PCE_Avium_Chro2 | 34929737 | 34934510 | CC-NB-LRR |
| PCE_A_Chro2G0339700.1 | PCE_Avium_Chro2 | 35180007 | 35183497 | RLP |
| PCE_A_Chro2G0346000.1 | PCE_Avium_Chro2 | 35912689 | 35918014 | CC-NB-LRR |
| PCE_A_Chro2G0348000.1 | PCE_Avium_Chro2 | 36144197 | 36147884 | CC-NB-LRR |
| PCE_A_Chro2G0357300.1 | PCE_Avium_Chro2 | 37359049 | 37361472 | SPTMK |
| PCE_A_Chro2G0357500.1 | PCE_Avium_Chro2 | 37437542 | 37439968 | SPTMK |
| PCE_A_Chro3G0012200.1 | PCE_Avium_Chro3 | 623119 | 629617 | LysM |
| PCE_A_Chro3G0013000.1 | PCE_Avium_Chro3 | 672217 | 677018 | RLK |
| PCE_A_Chro3G0013000.2 | PCE_Avium_Chro3 | 672217 | 677018 | RLK |
| PCE_A_Chro3G0013000.4 | PCE_Avium_Chro3 | 672217 | 677018 | RLK |
| PCE_A_Chro3G0028800.1 | PCE_Avium_Chro3 | 1619796 | 1623356 | SPTMK |
| PCE_A_Chro3G0037100.1 | PCE_Avium_Chro3 | 2073810 | 2075936 | RLK |
| PCE_A_Chro3G0043800.1 | PCE_Avium_Chro3 | 2438825 | 2441475 | RLK |
| PCE_A_Chro3G0063100.1 | PCE_Avium_Chro3 | 3836361 | 3843978 | SPTMK |
| PCE_A_Chro3G0063800.1 | PCE_Avium_Chro3 | 3897552 | 3900651 | SPTMK |
| PCE_A_Chro3G0064000.1 | PCE_Avium_Chro3 | 3907242 | 3910718 | SPTMK |
| PCE_A_Chro3G0064400.1 | PCE_Avium_Chro3 | 3917787 | 3920844 | SPTMK |
| PCE_A_Chro3G0064700.1 | PCE_Avium_Chro3 | 3937049 | 3940301 | SPTMK |
| PCE_A_Chro3G0064800.1 | PCE_Avium_Chro3 | 3944168 | 3957803 | SPTMK |
| PCE_A_Chro3G0081700.6 | PCE_Avium_Chro3 | 5264141 | 5268951 | RLK |
| PCE_A_Chro3G0081700.1 | PCE_Avium_Chro3 | 5264726 | 5268951 | RLK |
| PCE_A_Chro3G0081700.2 | PCE_Avium_Chro3 | 5264726 | 5268951 | RLK |
| PCE_A_Chro3G0081700.5 | PCE_Avium_Chro3 | 5264726 | 5268951 | RLK |
| PCE_A_Chro3G0081700.7 | PCE_Avium_Chro3 | 5264726 | 5268951 | RLK |
| PCE_A_Chro3G0081700.8 | PCE_Avium_Chro3 | 5264726 | 5268951 | RLK |
| PCE_A_Chro3G0087900.1 | PCE_Avium_Chro3 | 5806847 | 5814130 | RLK |
| PCE_A_Chro3G0087900.2 | PCE_Avium_Chro3 | 5806847 | 5814130 | RLK |
| PCE_A_Chro3G0087900.4 | PCE_Avium_Chro3 | 5806847 | 5814130 | RLK |
| PCE_A_Chro3G0087900.5 | PCE_Avium_Chro3 | 5806847 | 5814130 | RLK |
| PCE_A_Chro3G0090900.1 | PCE_Avium_Chro3 | 5950395 | 5950801 | LysM |
| PCE_A_Chro3G0090900.2 | PCE_Avium_Chro3 | 5950395 | 5950801 | LysM |
| PCE_A_Chro3G0091000.1 | PCE_Avium_Chro3 | 5968852 | 5969228 | LysM |
| PCE_A_Chro3G0091100.1 | PCE_Avium_Chro3 | 5974558 | 5974934 | LysM |
| PCE_A_Chro3G0093300.1 | PCE_Avium_Chro3 | 6093762 | 6096681 | SPTMK |
| PCE_A_Chro3G0093400.1 | PCE_Avium_Chro3 | 6128821 | 6131596 | SPTMK |
| PCE_A_Chro3G0093900.1 | PCE_Avium_Chro3 | 6191291 | 6193953 | SPTMK |
| PCE_A_Chro3G0094200.1 | PCE_Avium_Chro3 | 6226696 | 6229208 | SPTMK |
| PCE_A_Chro3G0094300.1 | PCE_Avium_Chro3 | 6234049 | 6236568 | SPTMK |
| PCE_A_Chro3G0094800.1 | PCE_Avium_Chro3 | 6295115 | 6299400 | SPTMK |
| PCE_A_Chro3G0094800.2 | PCE_Avium_Chro3 | 6295115 | 6299400 | SPTMK |
| PCE_A_Chro3G0094800.4 | PCE_Avium_Chro3 | 6295115 | 6299400 | SPTMK |
| PCE_A_Chro3G0094900.1 | PCE_Avium_Chro3 | 6318142 | 6322960 | SPTMK |
| PCE_A_Chro3G0094900.2 | PCE_Avium_Chro3 | 6318142 | 6322960 | SPTMK |
| PCE_A_Chro3G0094900.3 | PCE_Avium_Chro3 | 6318142 | 6322960 | SPTMK |
| PCE_A_Chro3G0094900.5 | PCE_Avium_Chro3 | 6318142 | 6322960 | SPTMK |
| PCE_A_Chro3G0094900.7 | PCE_Avium_Chro3 | 6318142 | 6322960 | SPTMK |
| PCE_A_Chro3G0095100.1 | PCE_Avium_Chro3 | 6360007 | 6364291 | SPTMK |
| PCE_A_Chro3G0095100.4 | PCE_Avium_Chro3 | 6360007 | 6363261 | SPTMK |
| PCE_A_Chro3G0095100.6 | PCE_Avium_Chro3 | 6360007 | 6364291 | SPTMK |
| PCE_A_Chro3G0095200.1 | PCE_Avium_Chro3 | 6374019 | 6377517 | SPTMK |
| PCE_A_Chro3G0095600.1 | PCE_Avium_Chro3 | 6450056 | 6456605 | SPTMK |
| PCE_A_Chro3G0095600.3 | PCE_Avium_Chro3 | 6450056 | 6455947 | SPTMK |
| PCE_A_Chro3G0095600.2 | PCE_Avium_Chro3 | 6450077 | 6456605 | SPTMK |
| PCE_A_Chro3G0096200.1 | PCE_Avium_Chro3 | 6551822 | 6557156 | SPTMK |
| PCE_A_Chro3G0096200.2 | PCE_Avium_Chro3 | 6551822 | 6557156 | SPTMK |
| PCE_A_Chro3G0096200.3 | PCE_Avium_Chro3 | 6551822 | 6556320 | SPTMK |
| PCE_A_Chro3G0096300.1 | PCE_Avium_Chro3 | 6562767 | 6565376 | SPTMK |
| PCE_A_Chro3G0100200.1 | PCE_Avium_Chro3 | 6802918 | 6805697 | SPTMK |
| PCE_A_Chro3G0100200.2 | PCE_Avium_Chro3 | 6802918 | 6805697 | SPTMK |
| PCE_A_Chro3G0108600.1 | PCE_Avium_Chro3 | 7394535 | 7432252 | LysM |
| PCE_A_Chro3G0108600.2 | PCE_Avium_Chro3 | 7394535 | 7432252 | LysM |
| PCE_A_Chro3G0108600.3 | PCE_Avium_Chro3 | 7394535 | 7409744 | SPTMK |
| PCE_A_Chro3G0116000.1 | PCE_Avium_Chro3 | 7929679 | 7932624 | RLP |
| PCE_A_Chro3G0118600.1 | PCE_Avium_Chro3 | 8071470 | 8074931 | RLP |
| PCE_A_Chro3G0118800.1 | PCE_Avium_Chro3 | 8090126 | 8093429 | RLP |
| PCE_A_Chro3G0118900.1 | PCE_Avium_Chro3 | 8109170 | 8112130 | RLP |
| PCE_A_Chro3G0132200.1 | PCE_Avium_Chro3 | 9382123 | 9386752 | SPTMK |
| PCE_A_Chro3G0132200.2 | PCE_Avium_Chro3 | 9382129 | 9386752 | SPTMK |
| PCE_A_Chro3G0132600.1 | PCE_Avium_Chro3 | 9443197 | 9446654 | SPTMK |
| PCE_A_Chro3G0132900.1 | PCE_Avium_Chro3 | 9461768 | 9465772 | SPTMK |
| PCE_A_Chro3G0133000.1 | PCE_Avium_Chro3 | 9466308 | 9469612 | SPTMK |
| PCE_A_Chro3G0133000.2 | PCE_Avium_Chro3 | 9466308 | 9469612 | SPTMK |
| PCE_A_Chro3G0133000.3 | PCE_Avium_Chro3 | 9466308 | 9469612 | SPTMK |
| PCE_A_Chro3G0133100.1 | PCE_Avium_Chro3 | 9482314 | 9485572 | SPTMK |
| PCE_A_Chro3G0133100.3 | PCE_Avium_Chro3 | 9482314 | 9485572 | SPTMK |
| PCE_A_Chro3G0133500.1 | PCE_Avium_Chro3 | 9526235 | 9529277 | SPTMK |
| PCE_A_Chro3G0133500.2 | PCE_Avium_Chro3 | 9526235 | 9529277 | SPTMK |
| PCE_A_Chro3G0133900.1 | PCE_Avium_Chro3 | 9601719 | 9605015 | SPTMK |
| PCE_A_Chro3G0133900.2 | PCE_Avium_Chro3 | 9601719 | 9605015 | SPTMK |
| PCE_A_Chro3G0133900.3 | PCE_Avium_Chro3 | 9601719 | 9605015 | SPTMK |
| PCE_A_Chro3G0134000.1 | PCE_Avium_Chro3 | 9611158 | 9614580 | SPTMK |
| PCE_A_Chro3G0134000.2 | PCE_Avium_Chro3 | 9611158 | 9614580 | SPTMK |
| PCE_A_Chro3G0134500.1 | PCE_Avium_Chro3 | 9644782 | 9647350 | SPTMK |
| PCE_A_Chro3G0134500.2 | PCE_Avium_Chro3 | 9644782 | 9647350 | SPTMK |
| PCE_A_Chro3G0134600.1 | PCE_Avium_Chro3 | 9649900 | 9653591 | SPTMK |
| PCE_A_Chro3G0134700.1 | PCE_Avium_Chro3 | 9654551 | 9657088 | SPTMK |
| PCE_A_Chro3G0143400.2 | PCE_Avium_Chro3 | 10351468 | 10354745 | RLK |
| PCE_A_Chro3G0145700.1 | PCE_Avium_Chro3 | 10672791 | 10674718 | RLP |
| PCE_A_Chro3G0146000.1 | PCE_Avium_Chro3 | 10700999 | 10708810 | RLK |
| PCE_A_Chro3G0146000.3 | PCE_Avium_Chro3 | 10700999 | 10708810 | RLK |
| PCE_A_Chro3G0146000.5 | PCE_Avium_Chro3 | 10700999 | 10708810 | RLK |
| PCE_A_Chro3G0146000.6 | PCE_Avium_Chro3 | 10700999 | 10708810 | RLK |
| PCE_A_Chro3G0146000.7 | PCE_Avium_Chro3 | 10700999 | 10708810 | RLK |
| PCE_A_Chro3G0146900.1 | PCE_Avium_Chro3 | 10778420 | 10781780 | RLK |
| PCE_A_Chro3G0146900.2 | PCE_Avium_Chro3 | 10778420 | 10781780 | RLK |
| PCE_A_Chro3G0146900.3 | PCE_Avium_Chro3 | 10778420 | 10781733 | RLK |
| PCE_A_Chro3G0147000.1 | PCE_Avium_Chro3 | 10805996 | 10810268 | RLK |
| PCE_A_Chro3G0150000.1 | PCE_Avium_Chro3 | 11145551 | 11149917 | RLK |
| PCE_A_Chro3G0157600.1 | PCE_Avium_Chro3 | 11900816 | 11903386 | RLP |
| PCE_A_Chro3G0162100.1 | PCE_Avium_Chro3 | 12399307 | 12402141 | SPTMK |
| PCE_A_Chro3G0162100.2 | PCE_Avium_Chro3 | 12399307 | 12402141 | SPTMK |
| PCE_A_Chro3G0171600.1 | PCE_Avium_Chro3 | 13819199 | 13823473 | SPTMK |
| PCE_A_Chro3G0171700.1 | PCE_Avium_Chro3 | 13828712 | 13832616 | SPTMK |
| PCE_A_Chro3G0173000.1 | PCE_Avium_Chro3 | 14202361 | 14204802 | SPTMK |
| PCE_A_Chro3G0174000.1 | PCE_Avium_Chro3 | 14285104 | 14287176 | SPTMK |
| PCE_A_Chro3G0176000.1 | PCE_Avium_Chro3 | 14467559 | 14469967 | SPTMK |
| PCE_A_Chro3G0194400.1 | PCE_Avium_Chro3 | 20447813 | 20449341 | LysM |
| PCE_A_Chro3G0199400.1 | PCE_Avium_Chro3 | 21063926 | 21067450 | SPTMK |
| PCE_A_Chro3G0208300.1 | PCE_Avium_Chro3 | 22282505 | 22287065 | RLK |
| PCE_A_Chro3G0212300.1 | PCE_Avium_Chro3 | 22916082 | 22920297 | SPTMK |
| PCE_A_Chro3G0212300.2 | PCE_Avium_Chro3 | 22916082 | 22920297 | SPTMK |
| PCE_A_Chro3G0215600.1 | PCE_Avium_Chro3 | 23327391 | 23330366 | CC-NB-LRR |
| PCE_A_Chro3G0220400.1 | PCE_Avium_Chro3 | 23705880 | 23708973 | CC-NB-LRR |
| PCE_A_Chro3G0221500.1 | PCE_Avium_Chro3 | 23788655 | 23791923 | CC-NB-LRR |
| PCE_A_Chro3G0221500.2 | PCE_Avium_Chro3 | 23788655 | 23792444 | CC-NB-LRR |
| PCE_A_Chro3G0221800.1 | PCE_Avium_Chro3 | 23816128 | 23819174 | CC-NB-LRR |
| PCE_A_Chro3G0222900.1 | PCE_Avium_Chro3 | 23943249 | 23946321 | CC-NB-LRR |
| PCE_A_Chro3G0224200.1 | PCE_Avium_Chro3 | 24225939 | 24229491 | SPTMK |
| PCE_A_Chro3G0227600.1 | PCE_Avium_Chro3 | 24517888 | 24527629 | CC-NB-LRR |
| PCE_A_Chro3G0239500.1 | PCE_Avium_Chro3 | 25518470 | 25521815 | SPTMK |
| PCE_A_Chro3G0239500.2 | PCE_Avium_Chro3 | 25518470 | 25521032 | SPTMK |
| PCE_A_Chro3G0250900.2 | PCE_Avium_Chro3 | 26770079 | 26776220 | LysM |
| PCE_A_Chro3G0250900.4 | PCE_Avium_Chro3 | 26770079 | 26776220 | LysM |
| PCE_A_Chro3G0250900.1 | PCE_Avium_Chro3 | 26770079 | 26776220 | LysM |
| PCE_A_Chro3G0250900.3 | PCE_Avium_Chro3 | 26770079 | 26776220 | LysM |
| PCE_A_Chro3G0253400.1 | PCE_Avium_Chro3 | 26983736 | 26988873 | RLK |
| PCE_A_Chro3G0253400.2 | PCE_Avium_Chro3 | 26983736 | 26988873 | RLK |
| PCE_A_Chro3G0253400.3 | PCE_Avium_Chro3 | 26983736 | 26988873 | RLK |
| PCE_A_Chro3G0253800.1 | PCE_Avium_Chro3 | 27032571 | 27044782 | RLK |
| PCE_A_Chro3G0254100.1 | PCE_Avium_Chro3 | 27077516 | 27083799 | RLK |
| PCE_A_Chro3G0254100.4 | PCE_Avium_Chro3 | 27077516 | 27083799 | RLK |
| PCE_A_Chro3G0254100.2 | PCE_Avium_Chro3 | 27079209 | 27083799 | RLK |
| PCE_A_Chro3G0254100.3 | PCE_Avium_Chro3 | 27079209 | 27083799 | RLK |
| PCE_A_Chro3G0254100.5 | PCE_Avium_Chro3 | 27079209 | 27083799 | RLK |
| PCE_A_Chro3G0254200.2 | PCE_Avium_Chro3 | 27094175 | 27120975 | RLK |
| PCE_A_Chro3G0254200.1 | PCE_Avium_Chro3 | 27108930 | 27120975 | RLK |
| PCE_A_Chro3G0254400.1 | PCE_Avium_Chro3 | 27148477 | 27153734 | RLK |
| PCE_A_Chro3G0255800.1 | PCE_Avium_Chro3 | 27336463 | 27340320 | RLK |
| PCE_A_Chro3G0256000.1 | PCE_Avium_Chro3 | 27346005 | 27350139 | RLK |
| PCE_A_Chro3G0256000.3 | PCE_Avium_Chro3 | 27346005 | 27350139 | RLK |
| PCE_A_Chro3G0262100.1 | PCE_Avium_Chro3 | 27872858 | 27877525 | SPTMK |
| PCE_A_Chro3G0274100.1 | PCE_Avium_Chro3 | 28678266 | 28680479 | RLK |
| PCE_A_Chro3G0282800.1 | PCE_Avium_Chro3 | 29274545 | 29282804 | RLK |
| PCE_A_Chro3G0283300.1 | PCE_Avium_Chro3 | 29314293 | 29318671 | RLK |
| PCE_A_Chro3G0284900.1 | PCE_Avium_Chro3 | 29473370 | 29479259 | RLK |
| PCE_A_Chro3G0286100.1 | PCE_Avium_Chro3 | 29592652 | 29595045 | RLK |
| PCE_A_Chro3G0286100.2 | PCE_Avium_Chro3 | 29592658 | 29595045 | RLK |
| PCE_A_Chro3G0286100.3 | PCE_Avium_Chro3 | 29592670 | 29595045 | RLK |
| PCE_A_Chro3G0286100.5 | PCE_Avium_Chro3 | 29592676 | 29595045 | RLK |
| PCE_A_Chro3G0286100.4 | PCE_Avium_Chro3 | 29592688 | 29595045 | RLK |
| PCE_A_Chro3G0287000.1 | PCE_Avium_Chro3 | 29645183 | 29647534 | RLP |
| PCE_A_Chro3G0287100.1 | PCE_Avium_Chro3 | 29675943 | 29678217 | RLP |
| PCE_A_Chro4G0013200.1 | PCE_Avium_Chro4 | 5895756 | 5903582 | RLK |
| PCE_A_Chro4G0013200.2 | PCE_Avium_Chro4 | 5895756 | 5903582 | RLK |
| PCE_A_Chro4G0018700.1 | PCE_Avium_Chro4 | 6849631 | 6852595 | SPTMK |
| PCE_A_Chro4G0029800.1 | PCE_Avium_Chro4 | 8672556 | 8675086 | RLP |
| PCE_A_Chro4G0030300.1 | PCE_Avium_Chro4 | 8818367 | 8820580 | RLP |
| PCE_A_Chro4G0035700.1 | PCE_Avium_Chro4 | 9322395 | 9325352 | RLP |
| PCE_A_Chro4G0035900.1 | PCE_Avium_Chro4 | 9345278 | 9348277 | RLP |
| PCE_A_Chro4G0036000.1 | PCE_Avium_Chro4 | 9353365 | 9356448 | RLP |
| PCE_A_Chro4G0036300.1 | PCE_Avium_Chro4 | 9395187 | 9398156 | RLP |
| PCE_A_Chro4G0039300.1 | PCE_Avium_Chro4 | 9823413 | 9829080 | SPTMK |
| PCE_A_Chro4G0039300.2 | PCE_Avium_Chro4 | 9823413 | 9829080 | SPTMK |
| PCE_A_Chro4G0039300.3 | PCE_Avium_Chro4 | 9823413 | 9829080 | SPTMK |
| PCE_A_Chro4G0039300.5 | PCE_Avium_Chro4 | 9823413 | 9829080 | SPTMK |
| PCE_A_Chro4G0039300.6 | PCE_Avium_Chro4 | 9823413 | 9829080 | SPTMK |
| PCE_A_Chro4G0039300.7 | PCE_Avium_Chro4 | 9823413 | 9829080 | SPTMK |
| PCE_A_Chro4G0039300.8 | PCE_Avium_Chro4 | 9823413 | 9829080 | SPTMK |
| PCE_A_Chro4G0039300.9 | PCE_Avium_Chro4 | 9823413 | 9829080 | SPTMK |
| PCE_A_Chro4G0041500.1 | PCE_Avium_Chro4 | 10159695 | 10162790 | RLP |
| PCE_A_Chro4G0041700.1 | PCE_Avium_Chro4 | 10181488 | 10184598 | RLP |
| PCE_A_Chro4G0041800.1 | PCE_Avium_Chro4 | 10203215 | 10206446 | RLP |
| PCE_A_Chro4G0042000.1 | PCE_Avium_Chro4 | 10250906 | 10253842 | RLP |
| PCE_A_Chro4G0043100.1 | PCE_Avium_Chro4 | 10346414 | 10348489 | RLK |
| PCE_A_Chro4G0043700.1 | PCE_Avium_Chro4 | 10399946 | 10409895 | RLK |
| PCE_A_Chro4G0043700.2 | PCE_Avium_Chro4 | 10399946 | 10409895 | RLK |
| PCE_A_Chro4G0043700.3 | PCE_Avium_Chro4 | 10399946 | 10409895 | RLK |
| PCE_A_Chro4G0044000.2 | PCE_Avium_Chro4 | 10445938 | 10452637 | RLK |
| PCE_A_Chro4G0044000.1 | PCE_Avium_Chro4 | 10445986 | 10452637 | RLK |
| PCE_A_Chro4G0044000.3 | PCE_Avium_Chro4 | 10446001 | 10452637 | RLK |
| PCE_A_Chro4G0044200.2 | PCE_Avium_Chro4 | 10467276 | 10474163 | RLK |
| PCE_A_Chro4G0044200.3 | PCE_Avium_Chro4 | 10467276 | 10474163 | RLK |
| PCE_A_Chro4G0049800.1 | PCE_Avium_Chro4 | 11249872 | 11255913 | TIR-NB-LRR |
| PCE_A_Chro4G0049900.1 | PCE_Avium_Chro4 | 11283077 | 11308364 | TIR-NB-LRR |
| PCE_A_Chro4G0074700.1 | PCE_Avium_Chro4 | 13495419 | 13501422 | SPTMK |
| PCE_A_Chro4G0074700.3 | PCE_Avium_Chro4 | 13495419 | 13501422 | SPTMK |
| PCE_A_Chro4G0074900.1 | PCE_Avium_Chro4 | 13502020 | 13509781 | SPTMK |
| PCE_A_Chro4G0075000.2 | PCE_Avium_Chro4 | 13512546 | 13519738 | SPTMK |
| PCE_A_Chro4G0075200.1 | PCE_Avium_Chro4 | 13585463 | 13590938 | SPTMK |
| PCE_A_Chro4G0075200.5 | PCE_Avium_Chro4 | 13585463 | 13590938 | SPTMK |
| PCE_A_Chro4G0075200.7 | PCE_Avium_Chro4 | 13585463 | 13590938 | SPTMK |
| PCE_A_Chro4G0075200.8 | PCE_Avium_Chro4 | 13586477 | 13590938 | SPTMK |
| PCE_A_Chro4G0075300.1 | PCE_Avium_Chro4 | 13602390 | 13605582 | SPTMK |
| PCE_A_Chro4G0075300.2 | PCE_Avium_Chro4 | 13602390 | 13605582 | SPTMK |
| PCE_A_Chro4G0075300.3 | PCE_Avium_Chro4 | 13602390 | 13605582 | SPTMK |
| PCE_A_Chro4G0075300.4 | PCE_Avium_Chro4 | 13602390 | 13605582 | SPTMK |
| PCE_A_Chro4G0075300.5 | PCE_Avium_Chro4 | 13602390 | 13605582 | SPTMK |
| PCE_A_Chro4G0075300.8 | PCE_Avium_Chro4 | 13602390 | 13605582 | SPTMK |
| PCE_A_Chro4G0075300.9 | PCE_Avium_Chro4 | 13602390 | 13605582 | SPTMK |
| PCE_A_Chro4G0075300.6 | PCE_Avium_Chro4 | 13603024 | 13605582 | SPTMK |
| PCE_A_Chro4G0075400.1 | PCE_Avium_Chro4 | 13623968 | 13628061 | SPTMK |
| PCE_A_Chro4G0075400.2 | PCE_Avium_Chro4 | 13624914 | 13628061 | SPTMK |
| PCE_A_Chro4G0075900.1 | PCE_Avium_Chro4 | 13691336 | 13700719 | SPTMK |
| PCE_A_Chro4G0076700.1 | PCE_Avium_Chro4 | 13758859 | 13762429 | SPTMK |
| PCE_A_Chro4G0077100.1 | PCE_Avium_Chro4 | 13804885 | 13808626 | SPTMK |
| PCE_A_Chro4G0077100.2 | PCE_Avium_Chro4 | 13804885 | 13808626 | SPTMK |
| PCE_A_Chro4G0077300.1 | PCE_Avium_Chro4 | 13822968 | 13827431 | SPTMK |
| PCE_A_Chro4G0077400.1 | PCE_Avium_Chro4 | 13830852 | 13836720 | SPTMK |
| PCE_A_Chro4G0082000.1 | PCE_Avium_Chro4 | 14269010 | 14272854 | SPTMK |
| PCE_A_Chro4G0092200.1 | PCE_Avium_Chro4 | 15181001 | 15185912 | RLK |
| PCE_A_Chro4G0094200.1 | PCE_Avium_Chro4 | 15302598 | 15304622 | SPTMK |
| PCE_A_Chro4G0094300.1 | PCE_Avium_Chro4 | 15306982 | 15309096 | SPTMK |
| PCE_A_Chro4G0094300.2 | PCE_Avium_Chro4 | 15306982 | 15309096 | SPTMK |
| PCE_A_Chro4G0094400.1 | PCE_Avium_Chro4 | 15313838 | 15315886 | SPTMK |
| PCE_A_Chro4G0094600.1 | PCE_Avium_Chro4 | 15333343 | 15335708 | SPTMK |
| PCE_A_Chro4G0094800.1 | PCE_Avium_Chro4 | 15362439 | 15373000 | SPTMK |
| PCE_A_Chro4G0094800.2 | PCE_Avium_Chro4 | 15362439 | 15373000 | SPTMK |
| PCE_A_Chro4G0095200.1 | PCE_Avium_Chro4 | 15442074 | 15444720 | SPTMK |
| PCE_A_Chro4G0095700.1 | PCE_Avium_Chro4 | 15541044 | 15543161 | SPTMK |
| PCE_A_Chro4G0096400.2 | PCE_Avium_Chro4 | 15583748 | 15592312 | SPTMK |
| PCE_A_Chro4G0096400.1 | PCE_Avium_Chro4 | 15584894 | 15592312 | SPTMK |
| PCE_A_Chro4G0097300.1 | PCE_Avium_Chro4 | 15698873 | 15700983 | SPTMK |
| PCE_A_Chro4G0097400.1 | PCE_Avium_Chro4 | 15705501 | 15722099 | SPTMK |
| PCE_A_Chro4G0098100.1 | PCE_Avium_Chro4 | 15784785 | 15786988 | SPTMK |
| PCE_A_Chro4G0098300.1 | PCE_Avium_Chro4 | 15794545 | 15796890 | SPTMK |
| PCE_A_Chro4G0098700.1 | PCE_Avium_Chro4 | 15810857 | 15812971 | SPTMK |
| PCE_A_Chro4G0098800.1 | PCE_Avium_Chro4 | 15815454 | 15817625 | SPTMK |
| PCE_A_Chro4G0098900.1 | PCE_Avium_Chro4 | 15821147 | 15823288 | SPTMK |
| PCE_A_Chro4G0102700.1 | PCE_Avium_Chro4 | 16141068 | 16143514 | RLP |
| PCE_A_Chro4G0102700.2 | PCE_Avium_Chro4 | 16141068 | 16143514 | RLP |
| PCE_A_Chro4G0102800.1 | PCE_Avium_Chro4 | 16170810 | 16173071 | RLP |
| PCE_A_Chro4G0103100.1 | PCE_Avium_Chro4 | 16201357 | 16203942 | RLP |
| PCE_A_Chro4G0103600.1 | PCE_Avium_Chro4 | 16220504 | 16222663 | RLP |
| PCE_A_Chro4G0103700.1 | PCE_Avium_Chro4 | 16233102 | 16235348 | RLP |
| PCE_A_Chro4G0104200.1 | PCE_Avium_Chro4 | 16312212 | 16314639 | RLP |
| PCE_A_Chro4G0104300.1 | PCE_Avium_Chro4 | 16326834 | 16329117 | RLP |
| PCE_A_Chro4G0104300.2 | PCE_Avium_Chro4 | 16326834 | 16329117 | RLP |
| PCE_A_Chro4G0104400.1 | PCE_Avium_Chro4 | 16346275 | 16348536 | RLP |
| PCE_A_Chro4G0104600.1 | PCE_Avium_Chro4 | 16356348 | 16358668 | RLP |
| PCE_A_Chro4G0106800.1 | PCE_Avium_Chro4 | 16580216 | 16582470 | RLP |
| PCE_A_Chro4G0110000.1 | PCE_Avium_Chro4 | 16861229 | 16870394 | RLK |
| PCE_A_Chro4G0112600.1 | PCE_Avium_Chro4 | 17080456 | 17091841 | RLK |
| PCE_A_Chro4G0120600.1 | PCE_Avium_Chro4 | 17707228 | 17711030 | CC-NB-LRR |
| PCE_A_Chro4G0121600.1 | PCE_Avium_Chro4 | 17764508 | 17767732 | RLP |
| PCE_A_Chro4G0124100.1 | PCE_Avium_Chro4 | 17918245 | 17923381 | RLK |
| PCE_A_Chro4G0124100.2 | PCE_Avium_Chro4 | 17918245 | 17923381 | RLK |
| PCE_A_Chro4G0124100.3 | PCE_Avium_Chro4 | 17918245 | 17924092 | RLK |
| PCE_A_Chro4G0126600.1 | PCE_Avium_Chro4 | 18177048 | 18184055 | SPTMK |
| PCE_A_Chro4G0126600.4 | PCE_Avium_Chro4 | 18177048 | 18183168 | SPTMK |
| PCE_A_Chro4G0126700.1 | PCE_Avium_Chro4 | 18184812 | 18188304 | SPTMK |
| PCE_A_Chro4G0126700.4 | PCE_Avium_Chro4 | 18184812 | 18187840 | SPTMK |
| PCE_A_Chro4G0126700.3 | PCE_Avium_Chro4 | 18184814 | 18188304 | SPTMK |
| PCE_A_Chro4G0126900.1 | PCE_Avium_Chro4 | 18189757 | 18197267 | SPTMK |
| PCE_A_Chro4G0126900.3 | PCE_Avium_Chro4 | 18189757 | 18197267 | SPTMK |
| PCE_A_Chro4G0126900.4 | PCE_Avium_Chro4 | 18189757 | 18196338 | SPTMK |
| PCE_A_Chro4G0127000.1 | PCE_Avium_Chro4 | 18216757 | 18224057 | SPTMK |
| PCE_A_Chro4G0127000.6 | PCE_Avium_Chro4 | 18216757 | 18219777 | SPTMK |
| PCE_A_Chro4G0127000.3 | PCE_Avium_Chro4 | 18216761 | 18224057 | SPTMK |
| PCE_A_Chro4G0127100.1 | PCE_Avium_Chro4 | 18225398 | 18229163 | SPTMK |
| PCE_A_Chro4G0127100.4 | PCE_Avium_Chro4 | 18225398 | 18228549 | SPTMK |
| PCE_A_Chro4G0127200.1 | PCE_Avium_Chro4 | 18229344 | 18234281 | SPTMK |
| PCE_A_Chro4G0127200.4 | PCE_Avium_Chro4 | 18229344 | 18233488 | SPTMK |
| PCE_A_Chro4G0134000.1 | PCE_Avium_Chro4 | 18721829 | 18725500 | SPTMK |
| PCE_A_Chro4G0134000.6 | PCE_Avium_Chro4 | 18721829 | 18725500 | SPTMK |
| PCE_A_Chro4G0134000.2 | PCE_Avium_Chro4 | 18722145 | 18725500 | SPTMK |
| PCE_A_Chro4G0134000.5 | PCE_Avium_Chro4 | 18722914 | 18725500 | SPTMK |
| PCE_A_Chro4G0136500.1 | PCE_Avium_Chro4 | 18914496 | 18923651 | RLK |
| PCE_A_Chro4G0136500.2 | PCE_Avium_Chro4 | 18914496 | 18923651 | RLK |
| PCE_A_Chro4G0136500.3 | PCE_Avium_Chro4 | 18914496 | 18923651 | RLK |
| PCE_A_Chro4G0138100.1 | PCE_Avium_Chro4 | 19079340 | 19082371 | SPTMK |
| PCE_A_Chro4G0138200.1 | PCE_Avium_Chro4 | 19083858 | 19086980 | SPTMK |
| PCE_A_Chro4G0138500.1 | PCE_Avium_Chro4 | 19114185 | 19117295 | SPTMK |
| PCE_A_Chro4G0138800.1 | PCE_Avium_Chro4 | 19153225 | 19158433 | SPTMK |
| PCE_A_Chro4G0138900.1 | PCE_Avium_Chro4 | 19166897 | 19172703 | SPTMK |
| PCE_A_Chro4G0139100.2 | PCE_Avium_Chro4 | 19179163 | 19182909 | SPTMK |
| PCE_A_Chro4G0139200.1 | PCE_Avium_Chro4 | 19192760 | 19196111 | SPTMK |
| PCE_A_Chro4G0139200.2 | PCE_Avium_Chro4 | 19192760 | 19196111 | SPTMK |
| PCE_A_Chro4G0142400.1 | PCE_Avium_Chro4 | 19461741 | 19464781 | RLP |
| PCE_A_Chro4G0147200.1 | PCE_Avium_Chro4 | 19935159 | 19938452 | RLP |
| PCE_A_Chro4G0147600.1 | PCE_Avium_Chro4 | 19969163 | 19972105 | RLP |
| PCE_A_Chro4G0147700.1 | PCE_Avium_Chro4 | 19975496 | 19979095 | RLP |
| PCE_A_Chro4G0149100.1 | PCE_Avium_Chro4 | 20058912 | 20063583 | RLK |
| PCE_A_Chro4G0149100.2 | PCE_Avium_Chro4 | 20058912 | 20063583 | RLK |
| PCE_A_Chro4G0150000.1 | PCE_Avium_Chro4 | 20154803 | 20159344 | RLK |
| PCE_A_Chro4G0154200.1 | PCE_Avium_Chro4 | 20534364 | 20537963 | RLP |
| PCE_A_Chro4G0154600.1 | PCE_Avium_Chro4 | 20555644 | 20558733 | RLP |
| PCE_A_Chro4G0169600.1 | PCE_Avium_Chro4 | 21900884 | 21910026 | RLK |
| PCE_A_Chro4G0169600.2 | PCE_Avium_Chro4 | 21900884 | 21910026 | RLK |
| PCE_A_Chro4G0169600.3 | PCE_Avium_Chro4 | 21900884 | 21910026 | RLK |
| PCE_A_Chro4G0169600.5 | PCE_Avium_Chro4 | 21900884 | 21910026 | RLK |
| PCE_A_Chro4G0169600.6 | PCE_Avium_Chro4 | 21900884 | 21910026 | RLK |
| PCE_A_Chro4G0169600.7 | PCE_Avium_Chro4 | 21900884 | 21910026 | RLK |
| PCE_A_Chro4G0169800.1 | PCE_Avium_Chro4 | 21934115 | 21947961 | RLK |
| PCE_A_Chro4G0169800.2 | PCE_Avium_Chro4 | 21934115 | 21947961 | RLK |
| PCE_A_Chro4G0169800.3 | PCE_Avium_Chro4 | 21934115 | 21947961 | RLK |
| PCE_A_Chro4G0169800.5 | PCE_Avium_Chro4 | 21934115 | 21947961 | RLK |
| PCE_A_Chro4G0169800.4 | PCE_Avium_Chro4 | 21941113 | 21947961 | RLK |
| PCE_A_Chro4G0170100.12 | PCE_Avium_Chro4 | 21964154 | 21975956 | RLK |
| PCE_A_Chro4G0170100.1 | PCE_Avium_Chro4 | 21964154 | 21975956 | RLK |
| PCE_A_Chro4G0170100.2 | PCE_Avium_Chro4 | 21964154 | 21975956 | RLK |
| PCE_A_Chro4G0170100.3 | PCE_Avium_Chro4 | 21964154 | 21975956 | RLK |
| PCE_A_Chro4G0170100.4 | PCE_Avium_Chro4 | 21964154 | 21975956 | RLK |
| PCE_A_Chro4G0170100.5 | PCE_Avium_Chro4 | 21964154 | 21975956 | RLK |
| PCE_A_Chro4G0170100.6 | PCE_Avium_Chro4 | 21964154 | 21975956 | RLK |
| PCE_A_Chro4G0170100.8 | PCE_Avium_Chro4 | 21964154 | 21975956 | RLK |
| PCE_A_Chro4G0174000.1 | PCE_Avium_Chro4 | 22238788 | 22241320 | SPTMK |
| PCE_A_Chro4G0174000.3 | PCE_Avium_Chro4 | 22238788 | 22241320 | SPTMK |
| PCE_A_Chro4G0174400.1 | PCE_Avium_Chro4 | 22273159 | 22274850 | RLK |
| PCE_A_Chro4G0174400.2 | PCE_Avium_Chro4 | 22273159 | 22274850 | RLK |
| PCE_A_Chro4G0179300.1 | PCE_Avium_Chro4 | 22638688 | 22641597 | SPTMK |
| PCE_A_Chro4G0179400.1 | PCE_Avium_Chro4 | 22642238 | 22645212 | SPTMK |
| PCE_A_Chro4G0179700.1 | PCE_Avium_Chro4 | 22657462 | 22686296 | SPTMK |
| PCE_A_Chro4G0179800.2 | PCE_Avium_Chro4 | 22699878 | 22702919 | SPTMK |
| PCE_A_Chro4G0180000.1 | PCE_Avium_Chro4 | 22729641 | 22732277 | SPTMK |
| PCE_A_Chro4G0180000.2 | PCE_Avium_Chro4 | 22729653 | 22732277 | SPTMK |
| PCE_A_Chro4G0180300.2 | PCE_Avium_Chro4 | 22757548 | 22760908 | SPTMK |
| PCE_A_Chro4G0180300.3 | PCE_Avium_Chro4 | 22757548 | 22760908 | SPTMK |
| PCE_A_Chro4G0180700.1 | PCE_Avium_Chro4 | 22786185 | 22791448 | SPTMK |
| PCE_A_Chro4G0180700.2 | PCE_Avium_Chro4 | 22786185 | 22791448 | SPTMK |
| PCE_A_Chro4G0180700.3 | PCE_Avium_Chro4 | 22786185 | 22791448 | SPTMK |
| PCE_A_Chro4G0180800.1 | PCE_Avium_Chro4 | 22796774 | 22799507 | SPTMK |
| PCE_A_Chro4G0180800.2 | PCE_Avium_Chro4 | 22796813 | 22799507 | SPTMK |
| PCE_A_Chro4G0180900.1 | PCE_Avium_Chro4 | 22819919 | 22823165 | SPTMK |
| PCE_A_Chro4G0181100.1 | PCE_Avium_Chro4 | 22847075 | 22849967 | SPTMK |
| PCE_A_Chro4G0181400.1 | PCE_Avium_Chro4 | 22879560 | 22884904 | SPTMK |
| PCE_A_Chro4G0181400.2 | PCE_Avium_Chro4 | 22879560 | 22884904 | SPTMK |
| PCE_A_Chro4G0181400.3 | PCE_Avium_Chro4 | 22879560 | 22884904 | SPTMK |
| PCE_A_Chro4G0181500.1 | PCE_Avium_Chro4 | 22894199 | 22897779 | SPTMK |
| PCE_A_Chro4G0181500.2 | PCE_Avium_Chro4 | 22894199 | 22897779 | SPTMK |
| PCE_A_Chro4G0181500.5 | PCE_Avium_Chro4 | 22894199 | 22897779 | SPTMK |
| PCE_A_Chro4G0181500.6 | PCE_Avium_Chro4 | 22894199 | 22897779 | SPTMK |
| PCE_A_Chro4G0181700.1 | PCE_Avium_Chro4 | 22935035 | 22939543 | SPTMK |
| PCE_A_Chro4G0181700.2 | PCE_Avium_Chro4 | 22935035 | 22939543 | SPTMK |
| PCE_A_Chro4G0182100.1 | PCE_Avium_Chro4 | 23006798 | 23009252 | SPTMK |
| PCE_A_Chro4G0182500.1 | PCE_Avium_Chro4 | 23026433 | 23028918 | SPTMK |
| PCE_A_Chro4G0182500.2 | PCE_Avium_Chro4 | 23026433 | 23028918 | SPTMK |
| PCE_A_Chro4G0183200.1 | PCE_Avium_Chro4 | 23077595 | 23083058 | RLK |
| PCE_A_Chro4G0183500.1 | PCE_Avium_Chro4 | 23106469 | 23111335 | SPTMK |
| PCE_A_Chro4G0192700.1 | PCE_Avium_Chro4 | 23711486 | 23716574 | RLK |
| PCE_A_Chro4G0192700.2 | PCE_Avium_Chro4 | 23711486 | 23721060 | RLK |
| PCE_A_Chro4G0192700.4 | PCE_Avium_Chro4 | 23711486 | 23721060 | RLK |
| PCE_A_Chro4G0192700.3 | PCE_Avium_Chro4 | 23711486 | 23716574 | RLK |
| PCE_A_Chro4G0197400.1 | PCE_Avium_Chro4 | 24008189 | 24015051 | RLK |
| PCE_A_Chro4G0204500.1 | PCE_Avium_Chro4 | 24425859 | 24433595 | RLK |
| PCE_A_Chro4G0204600.1 | PCE_Avium_Chro4 | 24435364 | 24442327 | RLK |
| PCE_A_Chro4G0204600.2 | PCE_Avium_Chro4 | 24435364 | 24442327 | RLK |
| PCE_A_Chro4G0204600.4 | PCE_Avium_Chro4 | 24435364 | 24442327 | RLK |
| PCE_A_Chro4G0204600.3 | PCE_Avium_Chro4 | 24435364 | 24442327 | RLK |
| PCE_A_Chro4G0204800.1 | PCE_Avium_Chro4 | 24487521 | 24498889 | RLK |
| PCE_A_Chro4G0204800.3 | PCE_Avium_Chro4 | 24487521 | 24498889 | RLK |
| PCE_A_Chro4G0205000.1 | PCE_Avium_Chro4 | 24506571 | 24515316 | RLK |
| PCE_A_Chro4G0205000.2 | PCE_Avium_Chro4 | 24506571 | 24513538 | RLK |
| PCE_A_Chro4G0205000.3 | PCE_Avium_Chro4 | 24506571 | 24513502 | RLK |
| PCE_A_Chro4G0205000.5 | PCE_Avium_Chro4 | 24506571 | 24515316 | RLK |
| PCE_A_Chro4G0205000.4 | PCE_Avium_Chro4 | 24506571 | 24515316 | RLK |
| PCE_A_Chro4G0205500.1 | PCE_Avium_Chro4 | 24544932 | 24553177 | RLK |
| PCE_A_Chro4G0205500.2 | PCE_Avium_Chro4 | 24544932 | 24552482 | RLK |
| PCE_A_Chro4G0205500.3 | PCE_Avium_Chro4 | 24544932 | 24553177 | RLK |
| PCE_A_Chro4G0205500.5 | PCE_Avium_Chro4 | 24544932 | 24553177 | RLK |
| PCE_A_Chro4G0205500.6 | PCE_Avium_Chro4 | 24544932 | 24553177 | RLK |
| PCE_A_Chro4G0205900.1 | PCE_Avium_Chro4 | 24564030 | 24571633 | RLK |
| PCE_A_Chro4G0205900.2 | PCE_Avium_Chro4 | 24564030 | 24571633 | RLK |
| PCE_A_Chro4G0205900.6 | PCE_Avium_Chro4 | 24564030 | 24571633 | RLK |
| PCE_A_Chro4G0209700.1 | PCE_Avium_Chro4 | 24816415 | 24818496 | RLP |
| PCE_A_Chro4G0210400.2 | PCE_Avium_Chro4 | 24833384 | 24837737 | RLK |
| PCE_A_Chro4G0210400.3 | PCE_Avium_Chro4 | 24833384 | 24837737 | RLK |
| PCE_A_Chro4G0210400.1 | PCE_Avium_Chro4 | 24833384 | 24837737 | RLK |
| PCE_A_Chro4G0210600.1 | PCE_Avium_Chro4 | 24847062 | 24863375 | RLK |
| PCE_A_Chro4G0211500.1 | PCE_Avium_Chro4 | 24951017 | 24953993 | RLK |
| PCE_A_Chro4G0212800.1 | PCE_Avium_Chro4 | 25044219 | 25050357 | RLK |
| PCE_A_Chro4G0212800.2 | PCE_Avium_Chro4 | 25044219 | 25050357 | RLK |
| PCE_A_Chro4G0221700.1 | PCE_Avium_Chro4 | 25514399 | 25518443 | RLK |
| PCE_A_Chro4G0227100.1 | PCE_Avium_Chro4 | 25826387 | 25833019 | RLK |
| PCE_A_Chro4G0227100.2 | PCE_Avium_Chro4 | 25826387 | 25833019 | RLK |
| PCE_A_Chro4G0227100.3 | PCE_Avium_Chro4 | 25826387 | 25831528 | RLK |
| PCE_A_Chro4G0228700.1 | PCE_Avium_Chro4 | 25947379 | 25947777 | LysM |
| PCE_A_Chro4G0233800.1 | PCE_Avium_Chro4 | 26239263 | 26243065 | SPTMK |
| PCE_A_Chro4G0233800.2 | PCE_Avium_Chro4 | 26239263 | 26243065 | SPTMK |
| PCE_A_Chro4G0233800.3 | PCE_Avium_Chro4 | 26239263 | 26243065 | SPTMK |
| PCE_A_Chro4G0233900.1 | PCE_Avium_Chro4 | 26243814 | 26247611 | SPTMK |
| PCE_A_Chro4G0233900.2 | PCE_Avium_Chro4 | 26243814 | 26247611 | SPTMK |
| PCE_A_Chro4G0234000.1 | PCE_Avium_Chro4 | 26248316 | 26254013 | SPTMK |
| PCE_A_Chro4G0234000.2 | PCE_Avium_Chro4 | 26248316 | 26252268 | SPTMK |
| PCE_A_Chro4G0234000.3 | PCE_Avium_Chro4 | 26248316 | 26252268 | SPTMK |
| PCE_A_Chro4G0234000.4 | PCE_Avium_Chro4 | 26248316 | 26252268 | SPTMK |
| PCE_A_Chro4G0234000.6 | PCE_Avium_Chro4 | 26248316 | 26252268 | SPTMK |
| PCE_A_Chro4G0234100.1 | PCE_Avium_Chro4 | 26254853 | 26259605 | SPTMK |
| PCE_A_Chro4G0234100.2 | PCE_Avium_Chro4 | 26254853 | 26258198 | SPTMK |
| PCE_A_Chro4G0234200.1 | PCE_Avium_Chro4 | 26262715 | 26267069 | SPTMK |
| PCE_A_Chro4G0234200.2 | PCE_Avium_Chro4 | 26262715 | 26267069 | SPTMK |
| PCE_A_Chro4G0234400.1 | PCE_Avium_Chro4 | 26274389 | 26277554 | SPTMK |
| PCE_A_Chro4G0234400.2 | PCE_Avium_Chro4 | 26274389 | 26277456 | SPTMK |
| PCE_A_Chro4G0234400.3 | PCE_Avium_Chro4 | 26274389 | 26276913 | SPTMK |
| PCE_A_Chro4G0234500.1 | PCE_Avium_Chro4 | 26278215 | 26281560 | SPTMK |
| PCE_A_Chro4G0234500.2 | PCE_Avium_Chro4 | 26278215 | 26281560 | SPTMK |
| PCE_A_Chro4G0234500.5 | PCE_Avium_Chro4 | 26278975 | 26281560 | SPTMK |
| PCE_A_Chro4G0234600.1 | PCE_Avium_Chro4 | 26283703 | 26286842 | SPTMK |
| PCE_A_Chro4G0234600.2 | PCE_Avium_Chro4 | 26283703 | 26286872 | SPTMK |
| PCE_A_Chro4G0234600.4 | PCE_Avium_Chro4 | 26284310 | 26286872 | SPTMK |
| PCE_A_Chro4G0234700.1 | PCE_Avium_Chro4 | 26297803 | 26300920 | SPTMK |
| PCE_A_Chro4G0234700.4 | PCE_Avium_Chro4 | 26297803 | 26300920 | SPTMK |
| PCE_A_Chro4G0234700.3 | PCE_Avium_Chro4 | 26298415 | 26300920 | SPTMK |
| PCE_A_Chro4G0234800.1 | PCE_Avium_Chro4 | 26304039 | 26307219 | SPTMK |
| PCE_A_Chro4G0234800.3 | PCE_Avium_Chro4 | 26304707 | 26307219 | SPTMK |
| PCE_A_Chro4G0234900.1 | PCE_Avium_Chro4 | 26309680 | 26313074 | SPTMK |
| PCE_A_Chro4G0235000.1 | PCE_Avium_Chro4 | 26314198 | 26317514 | SPTMK |
| PCE_A_Chro4G0235100.1 | PCE_Avium_Chro4 | 26318262 | 26321773 | SPTMK |
| PCE_A_Chro4G0235100.3 | PCE_Avium_Chro4 | 26318262 | 26321773 | SPTMK |
| PCE_A_Chro4G0237700.1 | PCE_Avium_Chro4 | 26458996 | 26461668 | SPTMK |
| PCE_A_Chro4G0237700.2 | PCE_Avium_Chro4 | 26458996 | 26461668 | SPTMK |
| PCE_A_Chro4G0237700.3 | PCE_Avium_Chro4 | 26458996 | 26461659 | SPTMK |
| PCE_A_Chro4G0237700.4 | PCE_Avium_Chro4 | 26458996 | 26461668 | SPTMK |
| PCE_A_Chro4G0237700.5 | PCE_Avium_Chro4 | 26458996 | 26461659 | SPTMK |
| PCE_A_Chro4G0237700.6 | PCE_Avium_Chro4 | 26458996 | 26461659 | SPTMK |
| PCE_A_Chro4G0237700.8 | PCE_Avium_Chro4 | 26458996 | 26461668 | SPTMK |
| PCE_A_Chro4G0237700.9 | PCE_Avium_Chro4 | 26458996 | 26461659 | SPTMK |
| PCE_A_Chro4G0237800.1 | PCE_Avium_Chro4 | 26462340 | 26485836 | SPTMK |
| PCE_A_Chro4G0238000.1 | PCE_Avium_Chro4 | 26486894 | 26490122 | SPTMK |
| PCE_A_Chro4G0238000.5 | PCE_Avium_Chro4 | 26486894 | 26490122 | SPTMK |
| PCE_A_Chro4G0238000.2 | PCE_Avium_Chro4 | 26488058 | 26490122 | SPTMK |
| PCE_A_Chro4G0238100.1 | PCE_Avium_Chro4 | 26499349 | 26502347 | SPTMK |
| PCE_A_Chro4G0238100.2 | PCE_Avium_Chro4 | 26499349 | 26502347 | SPTMK |
| PCE_A_Chro4G0238100.3 | PCE_Avium_Chro4 | 26499349 | 26502347 | SPTMK |
| PCE_A_Chro4G0238700.1 | PCE_Avium_Chro4 | 26530934 | 26533801 | SPTMK |
| PCE_A_Chro4G0238700.5 | PCE_Avium_Chro4 | 26530934 | 26533801 | SPTMK |
| PCE_A_Chro4G0238700.3 | PCE_Avium_Chro4 | 26531777 | 26533801 | SPTMK |
| PCE_A_Chro4G0239200.1 | PCE_Avium_Chro4 | 26573815 | 26576642 | SPTMK |
| PCE_A_Chro4G0239200.3 | PCE_Avium_Chro4 | 26574580 | 26576642 | SPTMK |
| PCE_A_Chro4G0239600.1 | PCE_Avium_Chro4 | 26623101 | 26626168 | SPTMK |
| PCE_A_Chro4G0239600.2 | PCE_Avium_Chro4 | 26623101 | 26626168 | SPTMK |
| PCE_A_Chro4G0239600.3 | PCE_Avium_Chro4 | 26623988 | 26626168 | SPTMK |
| PCE_A_Chro4G0239700.1 | PCE_Avium_Chro4 | 26627594 | 26631405 | SPTMK |
| PCE_A_Chro4G0239700.5 | PCE_Avium_Chro4 | 26627594 | 26631405 | SPTMK |
| PCE_A_Chro4G0239700.2 | PCE_Avium_Chro4 | 26628801 | 26631405 | SPTMK |
| PCE_A_Chro4G0240100.1 | PCE_Avium_Chro4 | 26663268 | 26675921 | SPTMK |
| PCE_A_Chro4G0240100.3 | PCE_Avium_Chro4 | 26663268 | 26675921 | SPTMK |
| PCE_A_Chro4G0240100.4 | PCE_Avium_Chro4 | 26663268 | 26675921 | SPTMK |
| PCE_A_Chro4G0240100.5 | PCE_Avium_Chro4 | 26663268 | 26675921 | SPTMK |
| PCE_A_Chro4G0240300.1 | PCE_Avium_Chro4 | 26680452 | 26684569 | SPTMK |
| PCE_A_Chro4G0240600.1 | PCE_Avium_Chro4 | 26693882 | 26696619 | SPTMK |
| PCE_A_Chro4G0241000.1 | PCE_Avium_Chro4 | 26715054 | 26718444 | SPTMK |
| PCE_A_Chro4G0241000.2 | PCE_Avium_Chro4 | 26715054 | 26718444 | SPTMK |
| PCE_A_Chro4G0241000.3 | PCE_Avium_Chro4 | 26715054 | 26718444 | SPTMK |
| PCE_A_Chro4G0241000.4 | PCE_Avium_Chro4 | 26715054 | 26718444 | SPTMK |
| PCE_A_Chro4G0241100.1 | PCE_Avium_Chro4 | 26749435 | 26752120 | SPTMK |
| PCE_A_Chro4G0241100.2 | PCE_Avium_Chro4 | 26749435 | 26752126 | SPTMK |
| PCE_A_Chro4G0241200.1 | PCE_Avium_Chro4 | 26753287 | 26755874 | SPTMK |
| PCE_A_Chro4G0252900.1 | PCE_Avium_Chro4 | 27340602 | 27345054 | SPTMK |
| PCE_A_Chro4G0252900.2 | PCE_Avium_Chro4 | 27340602 | 27345054 | SPTMK |
| PCE_A_Chro4G0252900.3 | PCE_Avium_Chro4 | 27340602 | 27345054 | SPTMK |
| PCE_A_Chro4G0252900.4 | PCE_Avium_Chro4 | 27340602 | 27345054 | SPTMK |
| PCE_A_Chro4G0253300.1 | PCE_Avium_Chro4 | 27358774 | 27364709 | RLK |
| PCE_A_Chro4G0253300.2 | PCE_Avium_Chro4 | 27358774 | 27364709 | RLK |
| PCE_A_Chro4G0253300.3 | PCE_Avium_Chro4 | 27358774 | 27364709 | RLK |
| PCE_A_Chro4G0256100.2 | PCE_Avium_Chro4 | 27508419 | 27509934 | SPTMK |
| PCE_A_Chro4G0259300.1 | PCE_Avium_Chro4 | 27676689 | 27680616 | CC-NB-LRR |
| PCE_A_Chro5G0002700.1 | PCE_Avium_Chro5 | 2644368 | 2647201 | LysM |
| PCE_A_Chro5G0007400.1 | PCE_Avium_Chro5 | 4406767 | 4410402 | CC-NB-LRR |
| PCE_A_Chro5G0007400.2 | PCE_Avium_Chro5 | 4406767 | 4410402 | CC-NB-LRR |
| PCE_A_Chro5G0007400.3 | PCE_Avium_Chro5 | 4406767 | 4410402 | CC-NB-LRR |
| PCE_A_Chro5G0007600.1 | PCE_Avium_Chro5 | 4427134 | 4430497 | CC-NB-LRR |
| PCE_A_Chro5G0007600.2 | PCE_Avium_Chro5 | 4427134 | 4430497 | CC-NB-LRR |
| PCE_A_Chro5G0007600.3 | PCE_Avium_Chro5 | 4427134 | 4430497 | CC-NB-LRR |
| PCE_A_Chro5G0007700.1 | PCE_Avium_Chro5 | 4466872 | 4471429 | CC-NB-LRR |
| PCE_A_Chro5G0007800.1 | PCE_Avium_Chro5 | 4474482 | 4477755 | CC-NB-LRR |
| PCE_A_Chro5G0007800.2 | PCE_Avium_Chro5 | 4474482 | 4477755 | CC-NB-LRR |
| PCE_A_Chro5G0007800.3 | PCE_Avium_Chro5 | 4474482 | 4477755 | CC-NB-LRR |
| PCE_A_Chro5G0007900.1 | PCE_Avium_Chro5 | 4478403 | 4481706 | CC-NB-LRR |
| PCE_A_Chro5G0007900.2 | PCE_Avium_Chro5 | 4478403 | 4481706 | CC-NB-LRR |
| PCE_A_Chro5G0007900.3 | PCE_Avium_Chro5 | 4478403 | 4481706 | CC-NB-LRR |
| PCE_A_Chro5G0008000.1 | PCE_Avium_Chro5 | 4505056 | 4508221 | CC-NB-LRR |
| PCE_A_Chro5G0022400.1 | PCE_Avium_Chro5 | 7210202 | 7212430 | RLP |
| PCE_A_Chro5G0029400.1 | PCE_Avium_Chro5 | 8494489 | 8499013 | SPTMK |
| PCE_A_Chro5G0029400.3 | PCE_Avium_Chro5 | 8494489 | 8499013 | SPTMK |
| PCE_A_Chro5G0041600.1 | PCE_Avium_Chro5 | 9982752 | 9985340 | CC-NB-LRR |
| PCE_A_Chro5G0041900.1 | PCE_Avium_Chro5 | 9995544 | 9998582 | CC-NB-LRR |
| PCE_A_Chro5G0042200.1 | PCE_Avium_Chro5 | 10062726 | 10066818 | TIR-NB-LRR |
| PCE_A_Chro5G0051600.1 | PCE_Avium_Chro5 | 11105388 | 11108782 | SPTMK |
| PCE_A_Chro5G0051600.2 | PCE_Avium_Chro5 | 11105388 | 11108782 | SPTMK |
| PCE_A_Chro5G0051600.3 | PCE_Avium_Chro5 | 11105388 | 11108782 | SPTMK |
| PCE_A_Chro5G0051600.4 | PCE_Avium_Chro5 | 11105388 | 11108782 | SPTMK |
| PCE_A_Chro5G0051700.1 | PCE_Avium_Chro5 | 11113494 | 11119026 | SPTMK |
| PCE_A_Chro5G0051700.2 | PCE_Avium_Chro5 | 11113494 | 11119026 | SPTMK |
| PCE_A_Chro5G0051700.3 | PCE_Avium_Chro5 | 11113494 | 11119026 | SPTMK |
| PCE_A_Chro5G0051700.4 | PCE_Avium_Chro5 | 11113494 | 11119026 | SPTMK |
| PCE_A_Chro5G0052200.1 | PCE_Avium_Chro5 | 11144843 | 11147854 | SPTMK |
| PCE_A_Chro5G0052200.2 | PCE_Avium_Chro5 | 11144843 | 11147854 | SPTMK |
| PCE_A_Chro5G0052200.3 | PCE_Avium_Chro5 | 11144843 | 11147854 | SPTMK |
| PCE_A_Chro5G0052200.4 | PCE_Avium_Chro5 | 11144843 | 11147854 | SPTMK |
| PCE_A_Chro5G0074100.1 | PCE_Avium_Chro5 | 13031337 | 13036445 | TIR-NB-LRR |
| PCE_A_Chro5G0074100.2 | PCE_Avium_Chro5 | 13031337 | 13036445 | TIR-NB-LRR |
| PCE_A_Chro5G0074200.1 | PCE_Avium_Chro5 | 13045336 | 13050515 | TIR-NB-LRR |
| PCE_A_Chro5G0080700.1 | PCE_Avium_Chro5 | 13640121 | 13642025 | SPTMK |
| PCE_A_Chro5G0089200.1 | PCE_Avium_Chro5 | 14372382 | 14377483 | RLK |
| PCE_A_Chro5G0101100.1 | PCE_Avium_Chro5 | 15340641 | 15343834 | CC-NB-LRR |
| PCE_A_Chro5G0103300.1 | PCE_Avium_Chro5 | 15571418 | 15574382 | RLK |
| PCE_A_Chro5G0106300.1 | PCE_Avium_Chro5 | 15728623 | 15731011 | SPTMK |
| PCE_A_Chro5G0144400.1 | PCE_Avium_Chro5 | 18154125 | 18157313 | RLK |
| PCE_A_Chro5G0144400.2 | PCE_Avium_Chro5 | 18154125 | 18157343 | RLK |
| PCE_A_Chro5G0144400.3 | PCE_Avium_Chro5 | 18154125 | 18157313 | RLK |
| PCE_A_Chro5G0152800.1 | PCE_Avium_Chro5 | 18743609 | 18745744 | RLK |
| PCE_A_Chro5G0158500.1 | PCE_Avium_Chro5 | 19124027 | 19126420 | SPTMK |
| PCE_A_Chro5G0160400.1 | PCE_Avium_Chro5 | 19209791 | 19212608 | SPTMK |
| PCE_A_Chro5G0160400.2 | PCE_Avium_Chro5 | 19209821 | 19212608 | SPTMK |
| PCE_A_Chro5G0162200.1 | PCE_Avium_Chro5 | 19285525 | 19288340 | SPTMK |
| PCE_A_Chro5G0162200.2 | PCE_Avium_Chro5 | 19285549 | 19288340 | SPTMK |
| PCE_A_Chro5G0163400.1 | PCE_Avium_Chro5 | 19349298 | 19354313 | RLK |
| PCE_A_Chro5G0164400.1 | PCE_Avium_Chro5 | 19405832 | 19409983 | RLP |
| PCE_A_Chro5G0164900.1 | PCE_Avium_Chro5 | 19434272 | 19437231 | SPTMK |
| PCE_A_Chro5G0170300.1 | PCE_Avium_Chro5 | 19812644 | 19814990 | RLK |
| PCE_A_Chro5G0193300.1 | PCE_Avium_Chro5 | 21041452 | 21044554 | SPTMK |
| PCE_A_Chro5G0194100.1 | PCE_Avium_Chro5 | 21105833 | 21109381 | RLK |
| PCE_A_Chro5G0194100.2 | PCE_Avium_Chro5 | 21105881 | 21109381 | RLK |
| PCE_A_Chro5G0208400.2 | PCE_Avium_Chro5 | 22058813 | 22060310 | RLP |
| PCE_A_Chro5G0212500.1 | PCE_Avium_Chro5 | 22291555 | 22293687 | LysM |
| PCE_A_Chro5G0212500.2 | PCE_Avium_Chro5 | 22291555 | 22293687 | LysM |
| PCE_A_Chro5G0212500.3 | PCE_Avium_Chro5 | 22291555 | 22292923 | LysM |
| PCE_A_Chro5G0215400.4 | PCE_Avium_Chro5 | 22446604 | 22451972 | SPTMK |
| PCE_A_Chro5G0225700.1 | PCE_Avium_Chro5 | 23000888 | 23003908 | RLK |
| PCE_A_Chro5G0225800.1 | PCE_Avium_Chro5 | 23078099 | 23080612 | RLK |
| PCE_A_Chro5G0225800.2 | PCE_Avium_Chro5 | 23078099 | 23080612 | RLK |
| PCE_A_Chro5G0225800.3 | PCE_Avium_Chro5 | 23078099 | 23080612 | RLK |
| PCE_A_Chro5G0234400.1 | PCE_Avium_Chro5 | 23746613 | 23748800 | RLK |
| PCE_A_Chro6G0014300.1 | PCE_Avium_Chro6 | 968659 | 970841 | RLP |
| PCE_A_Chro6G0014900.1 | PCE_Avium_Chro6 | 1011593 | 1014007 | SPTMK |
| PCE_A_Chro6G0015100.1 | PCE_Avium_Chro6 | 1032364 | 1034772 | SPTMK |
| PCE_A_Chro6G0015200.1 | PCE_Avium_Chro6 | 1037146 | 1043713 | SPTMK |
| PCE_A_Chro6G0015300.1 | PCE_Avium_Chro6 | 1047616 | 1050533 | SPTMK |
| PCE_A_Chro6G0015400.1 | PCE_Avium_Chro6 | 1051741 | 1054531 | SPTMK |
| PCE_A_Chro6G0018500.1 | PCE_Avium_Chro6 | 1313546 | 1316188 | SPTMK |
| PCE_A_Chro6G0018600.1 | PCE_Avium_Chro6 | 1318281 | 1330137 | SPTMK |
| PCE_A_Chro6G0018600.2 | PCE_Avium_Chro6 | 1318281 | 1330137 | SPTMK |
| PCE_A_Chro6G0018900.1 | PCE_Avium_Chro6 | 1340931 | 1343360 | SPTMK |
| PCE_A_Chro6G0022700.1 | PCE_Avium_Chro6 | 1669736 | 1672137 | SPTMK |
| PCE_A_Chro6G0034200.1 | PCE_Avium_Chro6 | 2578231 | 2583184 | RLK |
| PCE_A_Chro6G0037900.1 | PCE_Avium_Chro6 | 2846888 | 2855885 | RLK |
| PCE_A_Chro6G0037900.2 | PCE_Avium_Chro6 | 2846888 | 2855885 | RLK |
| PCE_A_Chro6G0050100.1 | PCE_Avium_Chro6 | 3631502 | 3636045 | RLK |
| PCE_A_Chro6G0072500.1 | PCE_Avium_Chro6 | 5190144 | 5193637 | RLK |
| PCE_A_Chro6G0072500.2 | PCE_Avium_Chro6 | 5190144 | 5193637 | RLK |
| PCE_A_Chro6G0072700.1 | PCE_Avium_Chro6 | 5210963 | 5214332 | RLK |
| PCE_A_Chro6G0072700.3 | PCE_Avium_Chro6 | 5210963 | 5214332 | RLK |
| PCE_A_Chro6G0072800.1 | PCE_Avium_Chro6 | 5219892 | 5223037 | RLK |
| PCE_A_Chro6G0072800.2 | PCE_Avium_Chro6 | 5219907 | 5223037 | RLK |
| PCE_A_Chro6G0081000.1 | PCE_Avium_Chro6 | 5780262 | 5786405 | TIR-NB-LRR |
| PCE_A_Chro6G0085600.1 | PCE_Avium_Chro6 | 6099237 | 6115590 | RLP |
| PCE_A_Chro6G0085800.1 | PCE_Avium_Chro6 | 6124483 | 6127715 | SPTMK |
| PCE_A_Chro6G0085900.1 | PCE_Avium_Chro6 | 6183449 | 6185960 | SPTMK |
| PCE_A_Chro6G0098000.1 | PCE_Avium_Chro6 | 7224600 | 7230865 | SPTMK |
| PCE_A_Chro6G0098000.2 | PCE_Avium_Chro6 | 7224600 | 7230865 | SPTMK |
| PCE_A_Chro6G0101400.1 | PCE_Avium_Chro6 | 7752311 | 7754149 | LysM |
| PCE_A_Chro6G0103200.1 | PCE_Avium_Chro6 | 7898754 | 7902880 | RLK |
| PCE_A_Chro6G0120400.1 | PCE_Avium_Chro6 | 10062221 | 10071811 | RLK |
| PCE_A_Chro6G0120400.2 | PCE_Avium_Chro6 | 10062221 | 10071811 | RLK |
| PCE_A_Chro6G0121700.1 | PCE_Avium_Chro6 | 10272861 | 10276201 | RLP |
| PCE_A_Chro6G0123100.1 | PCE_Avium_Chro6 | 10428620 | 10434408 | CC-NB-LRR |
| PCE_A_Chro6G0132500.1 | PCE_Avium_Chro6 | 11586362 | 11588355 | SPTMK |
| PCE_A_Chro6G0132500.3 | PCE_Avium_Chro6 | 11586362 | 11588355 | SPTMK |
| PCE_A_Chro6G0132500.4 | PCE_Avium_Chro6 | 11586362 | 11588355 | SPTMK |
| PCE_A_Chro6G0134200.1 | PCE_Avium_Chro6 | 11778487 | 11781454 | SPTMK |
| PCE_A_Chro6G0135500.1 | PCE_Avium_Chro6 | 11968373 | 11970389 | SPTMK |
| PCE_A_Chro6G0135500.3 | PCE_Avium_Chro6 | 11968373 | 11970389 | SPTMK |
| PCE_A_Chro6G0135500.4 | PCE_Avium_Chro6 | 11968373 | 11970389 | SPTMK |
| PCE_A_Chro6G0136600.1 | PCE_Avium_Chro6 | 12108291 | 12113072 | RLK |
| PCE_A_Chro6G0136600.2 | PCE_Avium_Chro6 | 12108291 | 12113072 | RLK |
| PCE_A_Chro6G0155300.1 | PCE_Avium_Chro6 | 15747471 | 15750554 | RLK |
| PCE_A_Chro6G0156900.1 | PCE_Avium_Chro6 | 15974233 | 15976967 | SPTMK |
| PCE_A_Chro6G0157000.1 | PCE_Avium_Chro6 | 15977980 | 15980680 | SPTMK |
| PCE_A_Chro6G0157100.1 | PCE_Avium_Chro6 | 15991269 | 15994401 | SPTMK |
| PCE_A_Chro6G0157200.1 | PCE_Avium_Chro6 | 15998584 | 16001395 | SPTMK |
| PCE_A_Chro6G0157300.1 | PCE_Avium_Chro6 | 16001913 | 16004707 | SPTMK |
| PCE_A_Chro6G0157600.1 | PCE_Avium_Chro6 | 16048328 | 16050652 | SPTMK |
| PCE_A_Chro6G0160400.2 | PCE_Avium_Chro6 | 19137862 | 19142246 | RLK |
| PCE_A_Chro6G0161100.2 | PCE_Avium_Chro6 | 19379003 | 19381259 | RLP |
| PCE_A_Chro6G0161100.3 | PCE_Avium_Chro6 | 19379003 | 19381259 | RLP |
| PCE_A_Chro6G0161100.1 | PCE_Avium_Chro6 | 19379016 | 19381259 | RLP |
| PCE_A_Chro6G0163500.1 | PCE_Avium_Chro6 | 19584287 | 19586521 | RLP |
| PCE_A_Chro6G0163500.2 | PCE_Avium_Chro6 | 19584287 | 19586521 | RLP |
| PCE_A_Chro6G0163600.1 | PCE_Avium_Chro6 | 19593754 | 19595868 | RLP |
| PCE_A_Chro6G0175100.1 | PCE_Avium_Chro6 | 21217884 | 21219660 | SPTMK |
| PCE_A_Chro6G0175200.1 | PCE_Avium_Chro6 | 21257276 | 21260414 | SPTMK |
| PCE_A_Chro6G0175200.2 | PCE_Avium_Chro6 | 21257276 | 21260414 | SPTMK |
| PCE_A_Chro6G0175700.3 | PCE_Avium_Chro6 | 21327976 | 21339212 | SPTMK |
| PCE_A_Chro6G0175700.5 | PCE_Avium_Chro6 | 21327976 | 21339212 | SPTMK |
| PCE_A_Chro6G0175700.2 | PCE_Avium_Chro6 | 21327988 | 21339212 | SPTMK |
| PCE_A_Chro6G0175700.1 | PCE_Avium_Chro6 | 21335870 | 21339212 | SPTMK |
| PCE_A_Chro6G0175700.4 | PCE_Avium_Chro6 | 21335870 | 21338247 | SPTMK |
| PCE_A_Chro6G0175700.6 | PCE_Avium_Chro6 | 21335870 | 21339212 | SPTMK |
| PCE_A_Chro6G0176400.1 | PCE_Avium_Chro6 | 21439553 | 21442357 | SPTMK |
| PCE_A_Chro6G0176400.3 | PCE_Avium_Chro6 | 21439553 | 21441697 | SPTMK |
| PCE_A_Chro6G0181200.1 | PCE_Avium_Chro6 | 21973499 | 21984586 | RLK |
| PCE_A_Chro6G0181200.2 | PCE_Avium_Chro6 | 21973499 | 21984586 | RLK |
| PCE_A_Chro6G0181200.3 | PCE_Avium_Chro6 | 21973499 | 21984586 | RLK |
| PCE_A_Chro6G0192900.1 | PCE_Avium_Chro6 | 23180331 | 23183417 | CC-NB-LRR |
| PCE_A_Chro6G0193200.1 | PCE_Avium_Chro6 | 23209751 | 23212450 | CC-NB-LRR |
| PCE_A_Chro6G0193200.2 | PCE_Avium_Chro6 | 23209868 | 23212450 | CC-NB-LRR |
| PCE_A_Chro6G0193900.1 | PCE_Avium_Chro6 | 23320379 | 23323577 | CC-NB-LRR |
| PCE_A_Chro6G0200600.1 | PCE_Avium_Chro6 | 23943194 | 23951808 | RLK |
| PCE_A_Chro6G0201200.1 | PCE_Avium_Chro6 | 24002424 | 24004369 | SPTMK |
| PCE_A_Chro6G0201400.1 | PCE_Avium_Chro6 | 24046640 | 24048715 | SPTMK |
| PCE_A_Chro6G0201600.1 | PCE_Avium_Chro6 | 24075625 | 24080061 | CC-NB-LRR |
| PCE_A_Chro6G0204400.1 | PCE_Avium_Chro6 | 24352837 | 24357313 | RLK |
| PCE_A_Chro6G0204700.1 | PCE_Avium_Chro6 | 24440839 | 24445038 | RLK |
| PCE_A_Chro6G0208500.1 | PCE_Avium_Chro6 | 24806654 | 24808265 | RLP |
| PCE_A_Chro6G0219000.1 | PCE_Avium_Chro6 | 25678215 | 25684254 | SPTMK |
| PCE_A_Chro6G0219100.1 | PCE_Avium_Chro6 | 25684491 | 25686906 | SPTMK |
| PCE_A_Chro6G0219200.1 | PCE_Avium_Chro6 | 25689838 | 25692969 | SPTMK |
| PCE_A_Chro6G0219400.1 | PCE_Avium_Chro6 | 25706596 | 25709242 | SPTMK |
| PCE_A_Chro6G0220400.1 | PCE_Avium_Chro6 | 25776053 | 25779473 | RLK |
| PCE_A_Chro6G0222200.1 | PCE_Avium_Chro6 | 25869557 | 25873855 | RLK |
| PCE_A_Chro6G0228600.1 | PCE_Avium_Chro6 | 26355887 | 26358299 | SPTMK |
| PCE_A_Chro6G0230400.1 | PCE_Avium_Chro6 | 26484999 | 26487823 | SPTMK |
| PCE_A_Chro6G0232700.1 | PCE_Avium_Chro6 | 26651409 | 26658273 | RLK |
| PCE_A_Chro6G0234100.1 | PCE_Avium_Chro6 | 26733655 | 26740537 | CC-NB-LRR |
| PCE_A_Chro6G0234100.2 | PCE_Avium_Chro6 | 26733655 | 26740537 | CC-NB-LRR |
| PCE_A_Chro6G0234100.3 | PCE_Avium_Chro6 | 26733655 | 26740537 | CC-NB-LRR |
| PCE_A_Chro6G0234800.1 | PCE_Avium_Chro6 | 26778815 | 26783805 | CC-NB-LRR |
| PCE_A_Chro6G0239600.1 | PCE_Avium_Chro6 | 27028053 | 27057187 | RLK |
| PCE_A_Chro6G0239700.1 | PCE_Avium_Chro6 | 27070031 | 27073415 | RLK |
| PCE_A_Chro6G0248800.1 | PCE_Avium_Chro6 | 27638945 | 27643200 | RLP |
| PCE_A_Chro6G0252400.1 | PCE_Avium_Chro6 | 27838546 | 27840779 | SPTMK |
| PCE_A_Chro6G0252500.1 | PCE_Avium_Chro6 | 27841729 | 27844078 | SPTMK |
| PCE_A_Chro6G0252600.1 | PCE_Avium_Chro6 | 27845220 | 27848794 | SPTMK |
| PCE_A_Chro6G0252800.1 | PCE_Avium_Chro6 | 27851837 | 27854138 | SPTMK |
| PCE_A_Chro6G0252800.4 | PCE_Avium_Chro6 | 27851837 | 27854138 | SPTMK |
| PCE_A_Chro6G0252800.5 | PCE_Avium_Chro6 | 27851837 | 27854138 | SPTMK |
| PCE_A_Chro6G0252900.1 | PCE_Avium_Chro6 | 27859101 | 27861941 | SPTMK |
| PCE_A_Chro6G0254000.1 | PCE_Avium_Chro6 | 27923291 | 27930052 | SPTMK |
| PCE_A_Chro6G0254000.2 | PCE_Avium_Chro6 | 27923291 | 27930052 | SPTMK |
| PCE_A_Chro6G0254100.1 | PCE_Avium_Chro6 | 27931202 | 27933356 | SPTMK |
| PCE_A_Chro6G0254400.1 | PCE_Avium_Chro6 | 27956120 | 27958259 | SPTMK |
| PCE_A_Chro6G0254400.2 | PCE_Avium_Chro6 | 27956120 | 27958259 | SPTMK |
| PCE_A_Chro6G0254400.4 | PCE_Avium_Chro6 | 27956120 | 27958259 | SPTMK |
| PCE_A_Chro6G0256200.1 | PCE_Avium_Chro6 | 28077677 | 28080058 | SPTMK |
| PCE_A_Chro6G0256300.1 | PCE_Avium_Chro6 | 28082730 | 28085108 | SPTMK |
| PCE_A_Chro6G0267600.1 | PCE_Avium_Chro6 | 28838230 | 28841609 | RLK |
| PCE_A_Chro6G0267800.1 | PCE_Avium_Chro6 | 28848921 | 28852527 | RLK |
| PCE_A_Chro6G0268000.1 | PCE_Avium_Chro6 | 28859151 | 28870432 | RLK |
| PCE_A_Chro6G0268500.1 | PCE_Avium_Chro6 | 28872431 | 28876145 | RLK |
| PCE_A_Chro6G0273200.1 | PCE_Avium_Chro6 | 29177215 | 29180541 | RLP |
| PCE_A_Chro6G0273300.1 | PCE_Avium_Chro6 | 29197597 | 29200976 | RLP |
| PCE_A_Chro6G0273400.1 | PCE_Avium_Chro6 | 29202745 | 29205969 | RLP |
| PCE_A_Chro6G0273500.1 | PCE_Avium_Chro6 | 29206924 | 29210422 | RLP |
| PCE_A_Chro6G0273600.1 | PCE_Avium_Chro6 | 29231550 | 29240106 | RLP |
| PCE_A_Chro6G0273900.1 | PCE_Avium_Chro6 | 29309872 | 29313213 | RLP |
| PCE_A_Chro6G0274100.1 | PCE_Avium_Chro6 | 29346896 | 29350783 | RLP |
| PCE_A_Chro6G0274200.1 | PCE_Avium_Chro6 | 29358395 | 29366497 | SPTMK |
| PCE_A_Chro6G0274200.2 | PCE_Avium_Chro6 | 29358395 | 29365431 | SPTMK |
| PCE_A_Chro6G0274200.4 | PCE_Avium_Chro6 | 29358395 | 29366497 | SPTMK |
| PCE_A_Chro6G0274200.5 | PCE_Avium_Chro6 | 29358395 | 29366497 | SPTMK |
| PCE_A_Chro6G0274300.1 | PCE_Avium_Chro6 | 29394945 | 29398132 | RLP |
| PCE_A_Chro6G0274400.1 | PCE_Avium_Chro6 | 29399696 | 29403744 | RLP |
| PCE_A_Chro6G0276700.1 | PCE_Avium_Chro6 | 29665550 | 29667577 | SPTMK |
| PCE_A_Chro6G0277100.1 | PCE_Avium_Chro6 | 29683798 | 29685816 | SPTMK |
| PCE_A_Chro6G0279100.1 | PCE_Avium_Chro6 | 29778231 | 29781477 | RLK |
| PCE_A_Chro6G0279100.2 | PCE_Avium_Chro6 | 29778231 | 29781477 | RLK |
| PCE_A_Chro6G0279100.3 | PCE_Avium_Chro6 | 29778231 | 29781477 | RLK |
| PCE_A_Chro6G0279100.4 | PCE_Avium_Chro6 | 29778231 | 29781477 | RLK |
| PCE_A_Chro6G0288900.1 | PCE_Avium_Chro6 | 30523132 | 30527618 | RLK |
| PCE_A_Chro6G0290900.1 | PCE_Avium_Chro6 | 30658893 | 30663972 | SPTMK |
| PCE_A_Chro6G0291400.1 | PCE_Avium_Chro6 | 30676965 | 30681430 | RLK |
| PCE_A_Chro6G0292000.1 | PCE_Avium_Chro6 | 30728528 | 30735307 | RLK |
| PCE_A_Chro6G0292000.2 | PCE_Avium_Chro6 | 30728528 | 30735307 | RLK |
| PCE_A_Chro6G0292000.3 | PCE_Avium_Chro6 | 30728528 | 30735307 | RLK |
| PCE_A_Chro6G0292000.5 | PCE_Avium_Chro6 | 30728528 | 30735307 | RLK |
| PCE_A_Chro6G0292000.4 | PCE_Avium_Chro6 | 30728528 | 30735307 | RLK |
| PCE_A_Chro6G0292100.1 | PCE_Avium_Chro6 | 30742731 | 30749214 | RLK |
| PCE_A_Chro6G0292100.2 | PCE_Avium_Chro6 | 30742731 | 30749214 | RLK |
| PCE_A_Chro6G0292100.4 | PCE_Avium_Chro6 | 30742731 | 30749214 | RLK |
| PCE_A_Chro6G0292100.5 | PCE_Avium_Chro6 | 30742731 | 30749214 | RLK |
| PCE_A_Chro6G0292100.6 | PCE_Avium_Chro6 | 30742731 | 30749214 | RLK |
| PCE_A_Chro6G0292100.3 | PCE_Avium_Chro6 | 30742731 | 30749214 | RLK |
| PCE_A_Chro6G0295900.1 | PCE_Avium_Chro6 | 30914699 | 30920860 | RLK |
| PCE_A_Chro6G0295900.2 | PCE_Avium_Chro6 | 30914699 | 30920860 | RLK |
| PCE_A_Chro6G0295900.3 | PCE_Avium_Chro6 | 30914699 | 30920860 | RLK |
| PCE_A_Chro6G0295900.4 | PCE_Avium_Chro6 | 30914699 | 30920860 | RLK |
| PCE_A_Chro6G0296000.1 | PCE_Avium_Chro6 | 30926399 | 30931951 | RLK |
| PCE_A_Chro6G0296000.2 | PCE_Avium_Chro6 | 30926399 | 30931951 | RLK |
| PCE_A_Chro6G0296000.3 | PCE_Avium_Chro6 | 30926399 | 30931951 | RLK |
| PCE_A_Chro6G0296400.1 | PCE_Avium_Chro6 | 30956594 | 30962934 | RLK |
| PCE_A_Chro6G0296400.2 | PCE_Avium_Chro6 | 30956594 | 30962934 | RLK |
| PCE_A_Chro6G0296400.3 | PCE_Avium_Chro6 | 30956594 | 30962934 | RLK |
| PCE_A_Chro6G0296400.4 | PCE_Avium_Chro6 | 30956594 | 30962934 | RLK |
| PCE_A_Chro6G0296400.5 | PCE_Avium_Chro6 | 30956594 | 30962934 | RLK |
| PCE_A_Chro6G0304400.1 | PCE_Avium_Chro6 | 31446696 | 31452878 | RLK |
| PCE_A_Chro6G0304400.2 | PCE_Avium_Chro6 | 31446696 | 31452878 | RLK |
| PCE_A_Chro6G0304400.3 | PCE_Avium_Chro6 | 31446696 | 31452878 | RLK |
| PCE_A_Chro6G0304400.4 | PCE_Avium_Chro6 | 31446696 | 31452878 | RLK |
| PCE_A_Chro6G0304400.7 | PCE_Avium_Chro6 | 31446696 | 31452878 | RLK |
| PCE_A_Chro6G0304500.1 | PCE_Avium_Chro6 | 31461472 | 31467184 | RLK |
| PCE_A_Chro6G0304500.2 | PCE_Avium_Chro6 | 31461472 | 31467184 | RLK |
| PCE_A_Chro6G0304500.3 | PCE_Avium_Chro6 | 31461472 | 31467184 | RLK |
| PCE_A_Chro6G0304600.1 | PCE_Avium_Chro6 | 31491844 | 31498373 | RLK |
| PCE_A_Chro6G0304600.2 | PCE_Avium_Chro6 | 31491844 | 31498373 | RLK |
| PCE_A_Chro6G0304600.3 | PCE_Avium_Chro6 | 31491844 | 31498373 | RLK |
| PCE_A_Chro6G0304800.1 | PCE_Avium_Chro6 | 31518781 | 31531917 | RLK |
| PCE_A_Chro6G0304800.2 | PCE_Avium_Chro6 | 31518781 | 31531917 | RLK |
| PCE_A_Chro6G0328100.1 | PCE_Avium_Chro6 | 33041404 | 33042311 | LysM |
| PCE_A_Chro6G0328100.2 | PCE_Avium_Chro6 | 33041404 | 33042307 | LysM |
| PCE_A_Chro6G0328100.3 | PCE_Avium_Chro6 | 33041404 | 33042311 | LysM |
| PCE_A_Chro6G0328900.1 | PCE_Avium_Chro6 | 33103385 | 33106979 | RLK |
| PCE_A_Chro6G0335100.1 | PCE_Avium_Chro6 | 33398581 | 33400446 | SPTMK |
| PCE_A_Chro6G0335200.1 | PCE_Avium_Chro6 | 33404191 | 33406095 | SPTMK |
| PCE_A_Chro6G0347800.1 | PCE_Avium_Chro6 | 34103569 | 34106199 | SPTMK |
| PCE_A_Chro6G0354000.1 | PCE_Avium_Chro6 | 34545258 | 34548418 | RLK |
| PCE_A_Chro6G0354500.1 | PCE_Avium_Chro6 | 34576753 | 34579866 | SPTMK |
| PCE_A_Chro6G0354500.2 | PCE_Avium_Chro6 | 34576753 | 34579866 | SPTMK |
| PCE_A_Chro6G0354600.1 | PCE_Avium_Chro6 | 34580219 | 34582399 | SPTMK |
| PCE_A_Chro6G0356200.1 | PCE_Avium_Chro6 | 34709332 | 34713778 | RLK |
| PCE_A_Chro6G0356200.4 | PCE_Avium_Chro6 | 34709332 | 34714445 | RLK |
| PCE_A_Chro6G0357300.1 | PCE_Avium_Chro6 | 34755903 | 34758506 | SPTMK |
| PCE_A_Chro6G0357300.3 | PCE_Avium_Chro6 | 34755903 | 34758506 | SPTMK |
| PCE_A_Chro6G0358500.1 | PCE_Avium_Chro6 | 34833601 | 34835889 | LysM |
| PCE_A_Chro6G0359500.1 | PCE_Avium_Chro6 | 34906563 | 34908832 | SPTMK |
| PCE_A_Chro6G0361200.1 | PCE_Avium_Chro6 | 34965347 | 34968794 | RLP |
| PCE_A_Chro6G0373200.1 | PCE_Avium_Chro6 | 35734271 | 35737926 | RLK |
| PCE_A_Chro6G0373800.1 | PCE_Avium_Chro6 | 35769052 | 35771234 | SPTMK |
| PCE_A_Chro7G0010200.1 | PCE_Avium_Chro7 | 2343454 | 2346099 | RLP |
| PCE_A_Chro7G0016000.1 | PCE_Avium_Chro7 | 3480504 | 3487396 | RLK |
| PCE_A_Chro7G0016000.2 | PCE_Avium_Chro7 | 3480504 | 3487396 | RLK |
| PCE_A_Chro7G0016000.3 | PCE_Avium_Chro7 | 3480504 | 3487396 | RLK |
| PCE_A_Chro7G0016000.4 | PCE_Avium_Chro7 | 3480504 | 3487396 | RLK |
| PCE_A_Chro7G0016000.5 | PCE_Avium_Chro7 | 3480504 | 3487396 | RLK |
| PCE_A_Chro7G0033300.1 | PCE_Avium_Chro7 | 6885134 | 6888158 | SPTMK |
| PCE_A_Chro7G0033300.2 | PCE_Avium_Chro7 | 6885134 | 6888158 | SPTMK |
| PCE_A_Chro7G0033300.3 | PCE_Avium_Chro7 | 6885134 | 6888158 | SPTMK |
| PCE_A_Chro7G0035200.1 | PCE_Avium_Chro7 | 7444903 | 7449845 | SPTMK |
| PCE_A_Chro7G0035200.3 | PCE_Avium_Chro7 | 7444903 | 7449845 | SPTMK |
| PCE_A_Chro7G0036400.1 | PCE_Avium_Chro7 | 7685529 | 7689462 | TIR-NB-LRR |
| PCE_A_Chro7G0042100.1 | PCE_Avium_Chro7 | 10696390 | 10699825 | RLK |
| PCE_A_Chro7G0047900.1 | PCE_Avium_Chro7 | 11838684 | 11841620 | RLP |
| PCE_A_Chro7G0048300.1 | PCE_Avium_Chro7 | 11888868 | 11891555 | RLP |
| PCE_A_Chro7G0048400.1 | PCE_Avium_Chro7 | 11911415 | 11916265 | RLP |
| PCE_A_Chro7G0049700.1 | PCE_Avium_Chro7 | 12111760 | 12114591 | RLP |
| PCE_A_Chro7G0050000.1 | PCE_Avium_Chro7 | 12151018 | 12155425 | RLP |
| PCE_A_Chro7G0052300.1 | PCE_Avium_Chro7 | 12576451 | 12598475 | TIR-NB-LRR |
| PCE_A_Chro7G0058900.1 | PCE_Avium_Chro7 | 13337061 | 13339382 | CC-NB-LRR |
| PCE_A_Chro7G0062900.1 | PCE_Avium_Chro7 | 14043331 | 14045697 | RLP |
| PCE_A_Chro7G0077700.1 | PCE_Avium_Chro7 | 15979480 | 15982570 | RLP |
| PCE_A_Chro7G0080400.1 | PCE_Avium_Chro7 | 16302048 | 16305883 | CC-NB-LRR |
| PCE_A_Chro7G0080600.1 | PCE_Avium_Chro7 | 16342013 | 16345007 | CC-NB-LRR |
| PCE_A_Chro7G0081000.1 | PCE_Avium_Chro7 | 16401620 | 16404110 | CC-NB-LRR |
| PCE_A_Chro7G0081000.2 | PCE_Avium_Chro7 | 16401620 | 16404110 | CC-NB-LRR |
| PCE_A_Chro7G0081000.3 | PCE_Avium_Chro7 | 16401620 | 16404110 | CC-NB-LRR |
| PCE_A_Chro7G0081200.1 | PCE_Avium_Chro7 | 16456980 | 16461212 | RLK |
| PCE_A_Chro7G0082100.1 | PCE_Avium_Chro7 | 16503033 | 16517549 | CC-NB-LRR |
| PCE_A_Chro7G0084400.1 | PCE_Avium_Chro7 | 16702595 | 16707787 | TIR-NB-LRR |
| PCE_A_Chro7G0084500.1 | PCE_Avium_Chro7 | 16709376 | 16718467 | TIR-NB-LRR |
| PCE_A_Chro7G0084700.1 | PCE_Avium_Chro7 | 16739032 | 16743432 | SPTMK |
| PCE_A_Chro7G0087500.1 | PCE_Avium_Chro7 | 17032839 | 17038034 | RLK |
| PCE_A_Chro7G0087500.2 | PCE_Avium_Chro7 | 17032839 | 17038034 | RLK |
| PCE_A_Chro7G0104800.1 | PCE_Avium_Chro7 | 19050313 | 19054350 | RLK |
| PCE_A_Chro7G0130300.1 | PCE_Avium_Chro7 | 21074499 | 21077415 | RLK |
| PCE_A_Chro7G0131800.1 | PCE_Avium_Chro7 | 21220675 | 21222567 | SPTMK |
| PCE_A_Chro7G0149400.1 | PCE_Avium_Chro7 | 22552251 | 22555867 | RPW8-NB-LRR |
| PCE_A_Chro7G0149600.1 | PCE_Avium_Chro7 | 22560440 | 22564002 | RPW8-NB-LRR |
| PCE_A_Chro7G0149600.2 | PCE_Avium_Chro7 | 22560440 | 22564002 | RPW8-NB-LRR |
| PCE_A_Chro7G0149700.2 | PCE_Avium_Chro7 | 22564765 | 22569957 | RPW8-NB-LRR |
| PCE_A_Chro7G0149700.1 | PCE_Avium_Chro7 | 22564765 | 22569957 | RPW8-NB-LRR |
| PCE_A_Chro7G0149700.3 | PCE_Avium_Chro7 | 22564765 | 22569957 | RPW8-NB-LRR |
| PCE_A_Chro7G0149800.1 | PCE_Avium_Chro7 | 22571203 | 22578064 | RPW8-NB-LRR |
| PCE_A_Chro7G0150000.1 | PCE_Avium_Chro7 | 22592379 | 22598522 | RPW8-NB-LRR |
| PCE_A_Chro7G0150100.1 | PCE_Avium_Chro7 | 22601666 | 22605425 | RPW8-NB-LRR |
| PCE_A_Chro7G0150100.3 | PCE_Avium_Chro7 | 22601666 | 22605425 | RPW8-NB-LRR |
| PCE_A_Chro7G0150100.2 | PCE_Avium_Chro7 | 22601666 | 22605425 | RPW8-NB-LRR |
| PCE_A_Chro7G0150200.1 | PCE_Avium_Chro7 | 22629531 | 22633546 | RPW8-NB-LRR |
| PCE_A_Chro7G0150400.1 | PCE_Avium_Chro7 | 22642506 | 22646314 | RPW8-NB-LRR |
| PCE_A_Chro7G0150400.2 | PCE_Avium_Chro7 | 22642506 | 22646314 | RPW8-NB-LRR |
| PCE_A_Chro7G0150500.1 | PCE_Avium_Chro7 | 22647166 | 22651417 | RPW8-NB-LRR |
| PCE_A_Chro7G0150500.2 | PCE_Avium_Chro7 | 22647166 | 22651417 | RPW8-NB-LRR |
| PCE_A_Chro7G0155600.1 | PCE_Avium_Chro7 | 22963964 | 22968921 | SPTMK |
| PCE_A_Chro7G0158000.1 | PCE_Avium_Chro7 | 23113420 | 23115279 | LysM |
| PCE_A_Chro7G0158100.1 | PCE_Avium_Chro7 | 23120686 | 23122599 | SPTMK |
| PCE_A_Chro7G0158200.1 | PCE_Avium_Chro7 | 23123215 | 23125434 | LysM |
| PCE_A_Chro7G0170100.1 | PCE_Avium_Chro7 | 23850756 | 23854863 | CC-NB-LRR |
| PCE_A_Chro7G0176100.1 | PCE_Avium_Chro7 | 24195397 | 24199894 | RLK |
| PCE_A_Chro7G0195200.1 | PCE_Avium_Chro7 | 25475003 | 25479207 | SPTMK |
| PCE_A_Chro7G0195200.2 | PCE_Avium_Chro7 | 25475003 | 25479207 | SPTMK |
| PCE_A_Chro7G0200000.1 | PCE_Avium_Chro7 | 25944961 | 25948789 | SPTMK |
| PCE_A_Chro7G0200000.2 | PCE_Avium_Chro7 | 25944961 | 25957177 | SPTMK |
| PCE_A_Chro7G0206300.1 | PCE_Avium_Chro7 | 26446594 | 26449408 | RLK |
| PCE_A_Chro7G0207400.1 | PCE_Avium_Chro7 | 26516680 | 26518910 | RLK |
| PCE_A_Chro7G0215600.1 | PCE_Avium_Chro7 | 26954703 | 26959201 | LysM |
| PCE_A_Chro7G0226700.1 | PCE_Avium_Chro7 | 27671106 | 27675491 | RLK |
| PCE_A_Chro7G0226700.2 | PCE_Avium_Chro7 | 27671106 | 27675491 | RLK |
| PCE_A_Chro7G0226700.3 | PCE_Avium_Chro7 | 27671106 | 27675491 | RLK |
| PCE_A_Chro7G0229100.1 | PCE_Avium_Chro7 | 27843114 | 27846740 | RLK |
| PCE_A_Chro7G0229200.1 | PCE_Avium_Chro7 | 27848328 | 27851805 | RLK |
| PCE_A_Chro7G0229300.1 | PCE_Avium_Chro7 | 27881016 | 27884391 | RLK |
| PCE_A_Chro7G0240300.1 | PCE_Avium_Chro7 | 28470184 | 28473452 | SPTMK |
| PCE_A_Chro7G0246000.1 | PCE_Avium_Chro7 | 28797730 | 28801501 | RLK |
| PCE_A_Chro7G0249300.1 | PCE_Avium_Chro7 | 28952019 | 28960875 | SPTMK |
| PCE_A_Chro7G0263400.1 | PCE_Avium_Chro7 | 29796484 | 29800780 | SPTMK |
| PCE_A_Chro7G0263400.2 | PCE_Avium_Chro7 | 29796484 | 29800780 | SPTMK |
| PCE_A_Chro7G0263400.4 | PCE_Avium_Chro7 | 29796484 | 29800780 | SPTMK |
| PCE_A_Chro7G0267400.1 | PCE_Avium_Chro7 | 30065335 | 30069713 | SPTMK |
| PCE_A_Chro7G0267400.2 | PCE_Avium_Chro7 | 30065335 | 30069713 | SPTMK |
| PCE_A_Chro7G0267400.3 | PCE_Avium_Chro7 | 30065335 | 30069713 | SPTMK |
| PCE_A_Chro7G0277500.1 | PCE_Avium_Chro7 | 30668201 | 30671869 | RLK |
| PCE_A_Chro7G0287500.1 | PCE_Avium_Chro7 | 31299713 | 31303454 | RLK |
| PCE_A_Chro7G0291400.1 | PCE_Avium_Chro7 | 31456559 | 31459634 | RLK |
| PCE_A_Chro7G0291400.2 | PCE_Avium_Chro7 | 31456559 | 31459628 | RLK |
| PCE_A_Chro8G0021400.1 | PCE_Avium_Chro8 | 1182869 | 1185492 | SPTMK |
| PCE_A_Chro8G0031600.1 | PCE_Avium_Chro8 | 1692524 | 1696704 | SPTMK |
| PCE_A_Chro8G0031600.2 | PCE_Avium_Chro8 | 1692524 | 1696704 | SPTMK |
| PCE_A_Chro8G0031600.4 | PCE_Avium_Chro8 | 1693969 | 1696704 | SPTMK |
| PCE_A_Chro8G0031800.1 | PCE_Avium_Chro8 | 1704510 | 1716020 | SPTMK |
| PCE_A_Chro8G0031800.2 | PCE_Avium_Chro8 | 1704510 | 1716020 | SPTMK |
| PCE_A_Chro8G0031900.1 | PCE_Avium_Chro8 | 1705808 | 1709238 | SPTMK |
| PCE_A_Chro8G0031900.2 | PCE_Avium_Chro8 | 1705808 | 1709211 | SPTMK |
| PCE_A_Chro8G0031900.5 | PCE_Avium_Chro8 | 1705808 | 1709238 | SPTMK |
| PCE_A_Chro8G0031900.4 | PCE_Avium_Chro8 | 1706539 | 1709238 | SPTMK |
| PCE_A_Chro8G0031800.4 | PCE_Avium_Chro8 | 1713216 | 1716020 | SPTMK |
| PCE_A_Chro8G0033100.1 | PCE_Avium_Chro8 | 1806448 | 1811415 | TIR-NB-LRR |
| PCE_A_Chro8G0035000.3 | PCE_Avium_Chro8 | 1997613 | 2006549 | SPTMK |
| PCE_A_Chro8G0044000.1 | PCE_Avium_Chro8 | 2510517 | 2512715 | RLP |
| PCE_A_Chro8G0053400.1 | PCE_Avium_Chro8 | 3101636 | 3105627 | RLK |
| PCE_A_Chro8G0053400.2 | PCE_Avium_Chro8 | 3101636 | 3105627 | RLK |
| PCE_A_Chro8G0053600.3 | PCE_Avium_Chro8 | 3110780 | 3114686 | RLK |
| PCE_A_Chro8G0053600.2 | PCE_Avium_Chro8 | 3110780 | 3114686 | RLK |
| PCE_A_Chro8G0053900.1 | PCE_Avium_Chro8 | 3144571 | 3148757 | RLK |
| PCE_A_Chro8G0055500.1 | PCE_Avium_Chro8 | 3228283 | 3231965 | RLK |
| PCE_A_Chro8G0060200.1 | PCE_Avium_Chro8 | 3497004 | 3501192 | RLK |
| PCE_A_Chro8G0060200.2 | PCE_Avium_Chro8 | 3497004 | 3501192 | RLK |
| PCE_A_Chro8G0060200.3 | PCE_Avium_Chro8 | 3497004 | 3501192 | RLK |
| PCE_A_Chro8G0061000.1 | PCE_Avium_Chro8 | 3545052 | 3548266 | RLK |
| PCE_A_Chro8G0066100.1 | PCE_Avium_Chro8 | 3903684 | 3908550 | RLK |
| PCE_A_Chro8G0066100.2 | PCE_Avium_Chro8 | 3903684 | 3908550 | RLK |
| PCE_A_Chro8G0081300.1 | PCE_Avium_Chro8 | 4778976 | 4782432 | RLK |
| PCE_A_Chro8G0081300.2 | PCE_Avium_Chro8 | 4778976 | 4782432 | RLK |
| PCE_A_Chro8G0081300.3 | PCE_Avium_Chro8 | 4778976 | 4782432 | RLK |
| PCE_A_Chro8G0081400.1 | PCE_Avium_Chro8 | 4783122 | 4783922 | RLP |
| PCE_A_Chro8G0081500.1 | PCE_Avium_Chro8 | 4785021 | 4785758 | RLP |
| PCE_A_Chro8G0082100.1 | PCE_Avium_Chro8 | 4843571 | 4844833 | RLP |
| PCE_A_Chro8G0083800.1 | PCE_Avium_Chro8 | 5005116 | 5010140 | TIR-NB-LRR |
| PCE_A_Chro8G0083800.2 | PCE_Avium_Chro8 | 5005596 | 5010140 | TIR-NB-LRR |
| PCE_A_Chro8G0085600.1 | PCE_Avium_Chro8 | 5090430 | 5094656 | SPTMK |
| PCE_A_Chro8G0089200.1 | PCE_Avium_Chro8 | 5336424 | 5341682 | TIR-NB-LRR |
| PCE_A_Chro8G0089200.5 | PCE_Avium_Chro8 | 5336424 | 5341682 | TIR-NB-LRR |
| PCE_A_Chro8G0089200.2 | PCE_Avium_Chro8 | 5336424 | 5341682 | TIR-NB-LRR |
| PCE_A_Chro8G0089300.1 | PCE_Avium_Chro8 | 5360369 | 5367588 | TIR-NB-LRR |
| PCE_A_Chro8G0089300.2 | PCE_Avium_Chro8 | 5360369 | 5365146 | TIR-NB-LRR |
| PCE_A_Chro8G0089400.1 | PCE_Avium_Chro8 | 5374019 | 5378821 | TIR-NB-LRR |
| PCE_A_Chro8G0089400.2 | PCE_Avium_Chro8 | 5374019 | 5378821 | TIR-NB-LRR |
| PCE_A_Chro8G0089400.4 | PCE_Avium_Chro8 | 5374019 | 5378821 | TIR-NB-LRR |
| PCE_A_Chro8G0089500.1 | PCE_Avium_Chro8 | 5384785 | 5390550 | TIR-NB-LRR |
| PCE_A_Chro8G0089500.2 | PCE_Avium_Chro8 | 5384785 | 5390550 | TIR-NB-LRR |
| PCE_A_Chro8G0093900.1 | PCE_Avium_Chro8 | 5808815 | 5811129 | LysM |
| PCE_A_Chro8G0094400.1 | PCE_Avium_Chro8 | 5838864 | 5842864 | TIR-NB-LRR |
| PCE_A_Chro8G0094800.2 | PCE_Avium_Chro8 | 5869270 | 5874480 | TIR-NB-LRR |
| PCE_A_Chro8G0094800.4 | PCE_Avium_Chro8 | 5869270 | 5874480 | TIR-NB-LRR |
| PCE_A_Chro8G0094800.1 | PCE_Avium_Chro8 | 5869270 | 5874480 | TIR-NB-LRR |
| PCE_A_Chro8G0094800.3 | PCE_Avium_Chro8 | 5869270 | 5874480 | TIR-NB-LRR |
| PCE_A_Chro8G0095400.2 | PCE_Avium_Chro8 | 5928508 | 5933958 | TIR-NB-LRR |
| PCE_A_Chro8G0095400.1 | PCE_Avium_Chro8 | 5928508 | 5933958 | TIR-NB-LRR |
| PCE_A_Chro8G0095400.3 | PCE_Avium_Chro8 | 5928508 | 5933958 | TIR-NB-LRR |
| PCE_A_Chro8G0112100.2 | PCE_Avium_Chro8 | 6984495 | 6988023 | RLK |
| PCE_A_Chro8G0112100.1 | PCE_Avium_Chro8 | 6984495 | 6988023 | RLK |
| PCE_A_Chro8G0130500.1 | PCE_Avium_Chro8 | 8394204 | 8397110 | RLK |
| PCE_A_Chro8G0135700.1 | PCE_Avium_Chro8 | 8754491 | 8758745 | RLK |
| PCE_A_Chro8G0138000.1 | PCE_Avium_Chro8 | 8863718 | 8866519 | CC-NB-LRR |
| PCE_A_Chro8G0138100.1 | PCE_Avium_Chro8 | 8885721 | 8889428 | CC-NB-LRR |
| PCE_A_Chro8G0138200.1 | PCE_Avium_Chro8 | 8895048 | 8897855 | CC-NB-LRR |
| PCE_A_Chro8G0138300.1 | PCE_Avium_Chro8 | 8921234 | 8924857 | CC-NB-LRR |
| PCE_A_Chro8G0138400.1 | PCE_Avium_Chro8 | 8929834 | 8932642 | CC-NB-LRR |
| PCE_A_Chro8G0138600.1 | PCE_Avium_Chro8 | 8958774 | 8962360 | CC-NB-LRR |
| PCE_A_Chro8G0138700.1 | PCE_Avium_Chro8 | 8964702 | 8966816 | CC-NB-LRR |
| PCE_A_Chro8G0139200.1 | PCE_Avium_Chro8 | 8981619 | 8984423 | CC-NB-LRR |
| PCE_A_Chro8G0147700.1 | PCE_Avium_Chro8 | 9621455 | 9627009 | SPTMK |
| PCE_A_Chro8G0148000.1 | PCE_Avium_Chro8 | 9640312 | 9643119 | CC-NB-LRR |
| PCE_A_Chro8G0148200.1 | PCE_Avium_Chro8 | 9647785 | 9650346 | CC-NB-LRR |
| PCE_A_Chro8G0148300.1 | PCE_Avium_Chro8 | 9655398 | 9658249 | CC-NB-LRR |
| PCE_A_Chro8G0158100.1 | PCE_Avium_Chro8 | 10336855 | 10341048 | CC-NB-LRR |
| PCE_A_Chro8G0161700.1 | PCE_Avium_Chro8 | 10632152 | 10637007 | TIR-NB-LRR |
| PCE_A_Chro8G0161700.2 | PCE_Avium_Chro8 | 10632152 | 10637007 | TIR-NB-LRR |
| PCE_A_Chro8G0164600.1 | PCE_Avium_Chro8 | 10810655 | 10817209 | TIR-NB-LRR |
| PCE_A_Chro8G0164700.1 | PCE_Avium_Chro8 | 10819938 | 10825379 | TIR-NB-LRR |
| PCE_A_Chro8G0164900.1 | PCE_Avium_Chro8 | 10828857 | 10835261 | TIR-NB-LRR |
| PCE_A_Chro8G0165100.1 | PCE_Avium_Chro8 | 10839683 | 10843925 | TIR-NB-LRR |
| PCE_A_Chro8G0167000.1 | PCE_Avium_Chro8 | 11053490 | 11057811 | TIR-NB-LRR |
| PCE_A_Chro8G0168100.1 | PCE_Avium_Chro8 | 11171166 | 11176275 | TIR-NB-LRR |
| PCE_A_Chro8G0179700.1 | PCE_Avium_Chro8 | 12218302 | 12225577 | RLK |
| PCE_A_Chro8G0179700.2 | PCE_Avium_Chro8 | 12220646 | 12225577 | RLK |
| PCE_A_Chro8G0186100.1 | PCE_Avium_Chro8 | 12802652 | 12805240 | SPTMK |
| PCE_A_Chro8G0196900.1 | PCE_Avium_Chro8 | 14110419 | 14114167 | RLK |
| PCE_A_Chro8G0196900.2 | PCE_Avium_Chro8 | 14110434 | 14114167 | RLK |
| PCE_A_Chro8G0197200.2 | PCE_Avium_Chro8 | 14152374 | 14156055 | RLK |
| PCE_A_Chro8G0197200.1 | PCE_Avium_Chro8 | 14152389 | 14156055 | RLK |
| PCE_A_Chro8G0203900.1 | PCE_Avium_Chro8 | 15226276 | 15230355 | TIR-NB-LRR |
| PCE_A_Chro8G0203900.2 | PCE_Avium_Chro8 | 15226276 | 15230355 | TIR-NB-LRR |
| PCE_A_Chro8G0203900.3 | PCE_Avium_Chro8 | 15226276 | 15230355 | TIR-NB-LRR |
| PCE_A_Chro8G0203900.4 | PCE_Avium_Chro8 | 15226276 | 15229942 | TIR-NB-LRR |
| PCE_A_Chro8G0204200.1 | PCE_Avium_Chro8 | 15243074 | 15255470 | RLK |
| PCE_A_Chro8G0209800.2 | PCE_Avium_Chro8 | 16217969 | 16220326 | RLK |
| PCE_A_Chro8G0209800.3 | PCE_Avium_Chro8 | 16217969 | 16220296 | RLK |
| PCE_A_Chro8G0221400.1 | PCE_Avium_Chro8 | 19214985 | 19217570 | RLP |
| PCE_A_Chro8G0221600.1 | PCE_Avium_Chro8 | 19233619 | 19236434 | RLP |
| PCE_A_Chro8G0222400.1 | PCE_Avium_Chro8 | 19558365 | 19561697 | RLP |
| PCE_A_Chro8G0224300.1 | PCE_Avium_Chro8 | 19979878 | 19982794 | SPTMK |
| PCE_A_Chro8G0224800.2 | PCE_Avium_Chro8 | 20048174 | 20050040 | RLP |
| PCE_A_Chro8G0225100.1 | PCE_Avium_Chro8 | 20111368 | 20113290 | RLP |
| PCE_A_Chro8G0227600.1 | PCE_Avium_Chro8 | 20534107 | 20542080 | RLK |
| PCE_A_Chro8G0235200.1 | PCE_Avium_Chro8 | 21865320 | 21868593 | TIR-NB-LRR |
| PCE_A_Chro8G0235300.1 | PCE_Avium_Chro8 | 21875318 | 21880591 | TIR-NB-LRR |
| PCE_A_Chro8G0236400.1 | PCE_Avium_Chro8 | 21983912 | 21989490 | TIR-NB-LRR |
| PCE_A_Chro8G0236400.2 | PCE_Avium_Chro8 | 21983912 | 21989490 | TIR-NB-LRR |
| PCE_A_Chro8G0238000.1 | PCE_Avium_Chro8 | 22155000 | 22159406 | RLK |
| PCE_A_Chro8G0238000.2 | PCE_Avium_Chro8 | 22155000 | 22159406 | RLK |
| PCE_A_Chro8G0240200.1 | PCE_Avium_Chro8 | 22506271 | 22510398 | TIR-NB-LRR |
| PCE_A_Chro8G0242000.1 | PCE_Avium_Chro8 | 22762942 | 22768199 | SPTMK |
| PCE_A_Chro8G0242100.1 | PCE_Avium_Chro8 | 22771099 | 22780259 | SPTMK |
| PCE_A_Chro8G0247500.1 | PCE_Avium_Chro8 | 23380135 | 23389277 | TIR-NB-LRR |
| PCE_A_Chro8G0247500.2 | PCE_Avium_Chro8 | 23380135 | 23389277 | TIR-NB-LRR |
| PCE_A_Chro8G0247500.3 | PCE_Avium_Chro8 | 23380135 | 23389277 | TIR-NB-LRR |
| PCE_A_Chro8G0247500.4 | PCE_Avium_Chro8 | 23380135 | 23389277 | TIR-NB-LRR |
| PCE_A_Chro8G0247500.7 | PCE_Avium_Chro8 | 23380135 | 23389277 | TIR-NB-LRR |
| PCE_A_Chro8G0247500.8 | PCE_Avium_Chro8 | 23380135 | 23389277 | TIR-NB-LRR |
| PCE_A_Chro8G0249300.1 | PCE_Avium_Chro8 | 23542739 | 23547793 | TIR-NB-LRR |
| PCE_A_Chro8G0251600.1 | PCE_Avium_Chro8 | 23726335 | 23736970 | TIR-NB-LRR |
| PCE_A_Chro8G0251600.2 | PCE_Avium_Chro8 | 23730449 | 23736970 | TIR-NB-LRR |
| PCE_A_Chro8G0251900.1 | PCE_Avium_Chro8 | 23770514 | 23777848 | TIR-NB-LRR |
| PCE_A_Chro8G0251900.3 | PCE_Avium_Chro8 | 23770514 | 23777848 | TIR-NB-LRR |
| PCE_A_Chro8G0251900.4 | PCE_Avium_Chro8 | 23770514 | 23777848 | TIR-NB-LRR |
| PCE_A_Chro8G0252100.2 | PCE_Avium_Chro8 | 23799036 | 23804476 | TIR-NB-LRR |
| PCE_A_Chro8G0252100.1 | PCE_Avium_Chro8 | 23799116 | 23804476 | TIR-NB-LRR |
| PCE_A_Chro8G0257900.1 | PCE_Avium_Chro8 | 24353939 | 24356182 | RLP |
| PCE_A_Chro8G0258500.2 | PCE_Avium_Chro8 | 24400003 | 24405675 | TIR-NB-LRR |
| PCE_A_Chro8G0258500.1 | PCE_Avium_Chro8 | 24400003 | 24405675 | TIR-NB-LRR |
| PCE_A_Chro8G0258500.3 | PCE_Avium_Chro8 | 24400003 | 24405675 | TIR-NB-LRR |
| PCE_A_Chro8G0258800.1 | PCE_Avium_Chro8 | 24448771 | 24452697 | TIR-NB-LRR |
| PCE_A_Chro8G0258800.2 | PCE_Avium_Chro8 | 24449483 | 24452697 | TIR-NB-LRR |
| PCE_A_Chro8G0260700.1 | PCE_Avium_Chro8 | 24643888 | 24646209 | SPTMK |
| PCE_A_Chro8G0261600.1 | PCE_Avium_Chro8 | 24748148 | 24754292 | TIR-NB-LRR |
| PCE_A_Chro8G0261600.2 | PCE_Avium_Chro8 | 24748148 | 24754292 | TIR-NB-LRR |
| PCE_A_Chro8G0262100.1 | PCE_Avium_Chro8 | 24778438 | 24783158 | TIR-NB-LRR |
| PCE_A_Chro8G0262100.2 | PCE_Avium_Chro8 | 24778438 | 24783158 | TIR-NB-LRR |
| PCE_A_Chro8G0262800.1 | PCE_Avium_Chro8 | 24817125 | 24822737 | TIR-NB-LRR |
| PCE_A_Chro8G0262800.2 | PCE_Avium_Chro8 | 24817125 | 24822737 | TIR-NB-LRR |
| PCE_A_Chro8G0263400.1 | PCE_Avium_Chro8 | 24877111 | 24886697 | TIR-NB-LRR |
| PCE_A_Chro8G0263400.2 | PCE_Avium_Chro8 | 24877111 | 24886697 | TIR-NB-LRR |
| PCE_A_Chro8G0263400.3 | PCE_Avium_Chro8 | 24877111 | 24886697 | TIR-NB-LRR |
| PCE_A_Chro8G0263400.4 | PCE_Avium_Chro8 | 24877111 | 24886697 | TIR-NB-LRR |
| PCE_A_Chro8G0263500.1 | PCE_Avium_Chro8 | 24894700 | 24900762 | TIR-NB-LRR |
| PCE_A_Chro8G0264100.1 | PCE_Avium_Chro8 | 24952915 | 24962797 | TIR-NB-LRR |
| PCE_A_Chro8G0264500.1 | PCE_Avium_Chro8 | 24975077 | 24978734 | TIR-NB-LRR |
| PCE_A_Chro8G0264800.1 | PCE_Avium_Chro8 | 25030141 | 25036389 | TIR-NB-LRR |
| PCE_A_Chro8G0264900.1 | PCE_Avium_Chro8 | 25040429 | 25043097 | SPTMK |
| PCE_A_Chro8G0265600.1 | PCE_Avium_Chro8 | 25139369 | 25143331 | RLK |
| PCE_A_Chro8G0266200.1 | PCE_Avium_Chro8 | 25235180 | 25237885 | CC-NB-LRR |
| PCE_A_Chro8G0272200.1 | PCE_Avium_Chro8 | 25848519 | 25852725 | SPTMK |
| PCE_A_Chro8G0272200.3 | PCE_Avium_Chro8 | 25848519 | 25851683 | SPTMK |
| PCE_A_Chro8G0272300.1 | PCE_Avium_Chro8 | 25854321 | 25858428 | SPTMK |
| PCE_A_Chro8G0272300.3 | PCE_Avium_Chro8 | 25854321 | 25858428 | SPTMK |
| PCE_A_Chro8G0275600.1 | PCE_Avium_Chro8 | 26223706 | 26226861 | CC-NB-LRR |
| PCE_A_Chro8G0277300.1 | PCE_Avium_Chro8 | 26447682 | 26450190 | CC-NB-LRR |
| PCE_A_Chro8G0277300.2 | PCE_Avium_Chro8 | 26447682 | 26450190 | CC-NB-LRR |
| PCE_A_Chro8G0277600.1 | PCE_Avium_Chro8 | 26485059 | 26487809 | CC-NB-LRR |
| PCE_A_Chro8G0277700.1 | PCE_Avium_Chro8 | 26535560 | 26538304 | CC-NB-LRR |
| PCE_A_Chro8G0277700.2 | PCE_Avium_Chro8 | 26535560 | 26538304 | CC-NB-LRR |
| PCE_A_Chro8G0277900.1 | PCE_Avium_Chro8 | 26593412 | 26597271 | CC-NB-LRR |
| PCE_A_Chro8G0277900.2 | PCE_Avium_Chro8 | 26593412 | 26597271 | CC-NB-LRR |
| PCE_A_Chro8G0278000.1 | PCE_Avium_Chro8 | 26601728 | 26604875 | CC-NB-LRR |
| PCE_A_Chro8G0278000.2 | PCE_Avium_Chro8 | 26601728 | 26604875 | CC-NB-LRR |
| PCE_A_Chro8G0279700.1 | PCE_Avium_Chro8 | 26830244 | 26834066 | RLK |
| PCE_A_Chro8G0282700.1 | PCE_Avium_Chro8 | 27077939 | 27082388 | TIR-NB-LRR |
| PCE_A_Chro8G0283800.1 | PCE_Avium_Chro8 | 27256749 | 27261215 | TIR-NB-LRR |
| PCE_A_Chro8G0283900.1 | PCE_Avium_Chro8 | 27284611 | 27291251 | TIR-NB-LRR |
| PCE_F_Chro1G0002900.1 | PCE_Fruticosa_Chro1 | 238145 | 241328 | SPTMK |
| PCE_F_Chro1G0007100.1 | PCE_Fruticosa_Chro1 | 557724 | 560550 | RLP |
| PCE_F_Chro1G0007200.1 | PCE_Fruticosa_Chro1 | 565840 | 568900 | RLP |
| PCE_F_Chro1G0007200.2 | PCE_Fruticosa_Chro1 | 565840 | 568900 | RLP |
| PCE_F_Chro1G0007600.1 | PCE_Fruticosa_Chro1 | 631761 | 634851 | RLP |
| PCE_F_Chro1G0007700.1 | PCE_Fruticosa_Chro1 | 636757 | 639960 | RLP |
| PCE_F_Chro1G0008900.1 | PCE_Fruticosa_Chro1 | 748012 | 753513 | RLK |
| PCE_F_Chro1G0008900.2 | PCE_Fruticosa_Chro1 | 748012 | 753513 | RLK |
| PCE_F_Chro1G0012700.1 | PCE_Fruticosa_Chro1 | 1007404 | 1015124 | CC-NB-LRR |
| PCE_F_Chro1G0018100.1 | PCE_Fruticosa_Chro1 | 1484054 | 1488678 | TIR-NB-LRR |
| PCE_F_Chro1G0018800.1 | PCE_Fruticosa_Chro1 | 1565808 | 1571400 | RLK |
| PCE_F_Chro1G0018800.2 | PCE_Fruticosa_Chro1 | 1565808 | 1571400 | RLK |
| PCE_F_Chro1G0022300.1 | PCE_Fruticosa_Chro1 | 1906799 | 1911183 | TIR-NB-LRR |
| PCE_F_Chro1G0022300.3 | PCE_Fruticosa_Chro1 | 1906799 | 1911183 | TIR-NB-LRR |
| PCE_F_Chro1G0024800.1 | PCE_Fruticosa_Chro1 | 2141688 | 2146093 | TIR-NB-LRR |
| PCE_F_Chro1G0026100.1 | PCE_Fruticosa_Chro1 | 2278805 | 2281759 | CC-NB-LRR |
| PCE_F_Chro1G0028500.1 | PCE_Fruticosa_Chro1 | 2572366 | 2574672 | CC-NB-LRR |
| PCE_F_Chro1G0028500.2 | PCE_Fruticosa_Chro1 | 2572366 | 2574672 | CC-NB-LRR |
| PCE_F_Chro1G0030300.1 | PCE_Fruticosa_Chro1 | 2708172 | 2710973 | CC-NB-LRR |
| PCE_F_Chro1G0033400.1 | PCE_Fruticosa_Chro1 | 2997975 | 3001947 | RLK |
| PCE_F_Chro1G0033500.2 | PCE_Fruticosa_Chro1 | 3009349 | 3013154 | RLK |
| PCE_F_Chro1G0033500.1 | PCE_Fruticosa_Chro1 | 3009349 | 3013154 | RLK |
| PCE_F_Chro1G0034000.1 | PCE_Fruticosa_Chro1 | 3066893 | 3070328 | RLP |
| PCE_F_Chro1G0034700.1 | PCE_Fruticosa_Chro1 | 3134818 | 3144587 | CC-NB-LRR |
| PCE_F_Chro1G0035700.1 | PCE_Fruticosa_Chro1 | 3267126 | 3270500 | RLP |
| PCE_F_Chro1G0037800.1 | PCE_Fruticosa_Chro1 | 3443325 | 3446606 | RLP |
| PCE_F_Chro1G0044400.1 | PCE_Fruticosa_Chro1 | 4058746 | 4067101 | CC-NB-LRR |
| PCE_F_Chro1G0044500.1 | PCE_Fruticosa_Chro1 | 4077294 | 4082025 | CC-NB-LRR |
| PCE_F_Chro1G0044600.1 | PCE_Fruticosa_Chro1 | 4083559 | 4089393 | CC-NB-LRR |
| PCE_F_Chro1G0048800.1 | PCE_Fruticosa_Chro1 | 4414990 | 4431984 | CC-NB-LRR |
| PCE_F_Chro1G0058100.1 | PCE_Fruticosa_Chro1 | 5108824 | 5112036 | RLK |
| PCE_F_Chro1G0058200.2 | PCE_Fruticosa_Chro1 | 5112483 | 5116105 | RLK |
| PCE_F_Chro1G0058300.1 | PCE_Fruticosa_Chro1 | 5116950 | 5120990 | RLK |
| PCE_F_Chro1G0058500.1 | PCE_Fruticosa_Chro1 | 5134474 | 5138268 | RLK |
| PCE_F_Chro1G0058500.2 | PCE_Fruticosa_Chro1 | 5134474 | 5138268 | RLK |
| PCE_F_Chro1G0058700.3 | PCE_Fruticosa_Chro1 | 5142939 | 5145986 | RLK |
| PCE_F_Chro1G0058700.1 | PCE_Fruticosa_Chro1 | 5142939 | 5146997 | RLK |
| PCE_F_Chro1G0058700.2 | PCE_Fruticosa_Chro1 | 5142939 | 5146997 | RLK |
| PCE_F_Chro1G0060100.1 | PCE_Fruticosa_Chro1 | 5229412 | 5232936 | RLK |
| PCE_F_Chro1G0070000.1 | PCE_Fruticosa_Chro1 | 6100204 | 6102653 | LysM |
| PCE_F_Chro1G0072400.1 | PCE_Fruticosa_Chro1 | 6273914 | 6277444 | SPTMK |
| PCE_F_Chro1G0077800.1 | PCE_Fruticosa_Chro1 | 6676198 | 6678203 | SPTMK |
| PCE_F_Chro1G0081800.1 | PCE_Fruticosa_Chro1 | 7030033 | 7033649 | SPTMK |
| PCE_F_Chro1G0086900.1 | PCE_Fruticosa_Chro1 | 7363447 | 7367715 | RLP |
| PCE_F_Chro1G0087500.1 | PCE_Fruticosa_Chro1 | 7408122 | 7412013 | SPTMK |
| PCE_F_Chro1G0092300.4 | PCE_Fruticosa_Chro1 | 7755425 | 7759279 | SPTMK |
| PCE_F_Chro1G0092300.2 | PCE_Fruticosa_Chro1 | 7755746 | 7759279 | SPTMK |
| PCE_F_Chro1G0092300.1 | PCE_Fruticosa_Chro1 | 7756570 | 7759279 | SPTMK |
| PCE_F_Chro1G0092700.1 | PCE_Fruticosa_Chro1 | 7793452 | 7801327 | SPTMK |
| PCE_F_Chro1G0101400.1 | PCE_Fruticosa_Chro1 | 8435476 | 8438463 | SPTMK |
| PCE_F_Chro1G0101400.2 | PCE_Fruticosa_Chro1 | 8435476 | 8438463 | SPTMK |
| PCE_F_Chro1G0101400.3 | PCE_Fruticosa_Chro1 | 8436264 | 8438463 | SPTMK |
| PCE_F_Chro1G0101500.1 | PCE_Fruticosa_Chro1 | 8456705 | 8469679 | SPTMK |
| PCE_F_Chro1G0101500.2 | PCE_Fruticosa_Chro1 | 8456705 | 8469679 | SPTMK |
| PCE_F_Chro1G0101500.3 | PCE_Fruticosa_Chro1 | 8467459 | 8469679 | SPTMK |
| PCE_F_Chro1G0101500.4 | PCE_Fruticosa_Chro1 | 8467459 | 8469679 | SPTMK |
| PCE_F_Chro1G0101700.1 | PCE_Fruticosa_Chro1 | 8485355 | 8488477 | SPTMK |
| PCE_F_Chro1G0102200.1 | PCE_Fruticosa_Chro1 | 8532419 | 8536034 | SPTMK |
| PCE_F_Chro1G0119900.1 | PCE_Fruticosa_Chro1 | 10008102 | 10010756 | SPTMK |
| PCE_F_Chro1G0119900.2 | PCE_Fruticosa_Chro1 | 10008102 | 10010753 | SPTMK |
| PCE_F_Chro1G0119900.3 | PCE_Fruticosa_Chro1 | 10008102 | 10010753 | SPTMK |
| PCE_F_Chro1G0122000.1 | PCE_Fruticosa_Chro1 | 10132744 | 10136514 | RLK |
| PCE_F_Chro1G0129200.1 | PCE_Fruticosa_Chro1 | 10604913 | 10608201 | RLK |
| PCE_F_Chro1G0130700.1 | PCE_Fruticosa_Chro1 | 10700514 | 10702917 | SPTMK |
| PCE_F_Chro1G0130800.1 | PCE_Fruticosa_Chro1 | 10704583 | 10707148 | SPTMK |
| PCE_F_Chro1G0130900.1 | PCE_Fruticosa_Chro1 | 10708388 | 10710787 | SPTMK |
| PCE_F_Chro1G0131000.1 | PCE_Fruticosa_Chro1 | 10711118 | 10713670 | SPTMK |
| PCE_F_Chro1G0132400.1 | PCE_Fruticosa_Chro1 | 10793953 | 10800292 | RLK |
| PCE_F_Chro1G0132800.1 | PCE_Fruticosa_Chro1 | 10816545 | 10819495 | RLK |
| PCE_F_Chro1G0147900.1 | PCE_Fruticosa_Chro1 | 11732450 | 11734630 | RLK |
| PCE_F_Chro1G0159400.1 | PCE_Fruticosa_Chro1 | 12471626 | 12479643 | RLK |
| PCE_F_Chro1G0159400.2 | PCE_Fruticosa_Chro1 | 12471626 | 12479643 | RLK |
| PCE_F_Chro1G0159400.3 | PCE_Fruticosa_Chro1 | 12471626 | 12479643 | RLK |
| PCE_F_Chro1G0169000.1 | PCE_Fruticosa_Chro1 | 13034018 | 13037406 | RPW8-NB-LRR |
| PCE_F_Chro1G0171900.1 | PCE_Fruticosa_Chro1 | 13227510 | 13232063 | SPTMK |
| PCE_F_Chro1G0177800.1 | PCE_Fruticosa_Chro1 | 13708768 | 13714199 | RLK |
| PCE_F_Chro1G0177800.2 | PCE_Fruticosa_Chro1 | 13708768 | 13714199 | RLK |
| PCE_F_Chro1G0177800.3 | PCE_Fruticosa_Chro1 | 13708768 | 13714199 | RLK |
| PCE_F_Chro1G0186400.1 | PCE_Fruticosa_Chro1 | 14292208 | 14296038 | RLK |
| PCE_F_Chro1G0193300.1 | PCE_Fruticosa_Chro1 | 14749724 | 14753829 | RLK |
| PCE_F_Chro1G0208900.1 | PCE_Fruticosa_Chro1 | 15727774 | 15731001 | LysM |
| PCE_F_Chro1G0208900.2 | PCE_Fruticosa_Chro1 | 15727774 | 15731001 | LysM |
| PCE_F_Chro1G0208900.3 | PCE_Fruticosa_Chro1 | 15727774 | 15731001 | LysM |
| PCE_F_Chro1G0213700.1 | PCE_Fruticosa_Chro1 | 16151567 | 16156218 | RLK |
| PCE_F_Chro1G0214000.1 | PCE_Fruticosa_Chro1 | 16173326 | 16175972 | RLP |
| PCE_F_Chro1G0214100.1 | PCE_Fruticosa_Chro1 | 16179681 | 16188983 | RLK |
| PCE_F_Chro1G0218600.1 | PCE_Fruticosa_Chro1 | 16581287 | 16585327 | RLK |
| PCE_F_Chro1G0220300.1 | PCE_Fruticosa_Chro1 | 16760473 | 16764849 | RLK |
| PCE_F_Chro1G0220600.1 | PCE_Fruticosa_Chro1 | 16789427 | 16792015 | SPTMK |
| PCE_F_Chro1G0235300.1 | PCE_Fruticosa_Chro1 | 17744759 | 17747991 | RLK |
| PCE_F_Chro1G0236400.1 | PCE_Fruticosa_Chro1 | 17783769 | 17787485 | SPTMK |
| PCE_F_Chro1G0236400.2 | PCE_Fruticosa_Chro1 | 17783769 | 17787485 | SPTMK |
| PCE_F_Chro1G0236600.1 | PCE_Fruticosa_Chro1 | 17801008 | 17804564 | SPTMK |
| PCE_F_Chro1G0237200.1 | PCE_Fruticosa_Chro1 | 17826917 | 17829659 | SPTMK |
| PCE_F_Chro1G0237400.1 | PCE_Fruticosa_Chro1 | 17830198 | 17836584 | SPTMK |
| PCE_F_Chro1G0237400.2 | PCE_Fruticosa_Chro1 | 17830198 | 17836584 | SPTMK |
| PCE_F_Chro1G0237800.1 | PCE_Fruticosa_Chro1 | 17873216 | 17876022 | SPTMK |
| PCE_F_Chro1G0246400.1 | PCE_Fruticosa_Chro1 | 18456553 | 18461697 | RLP |
| PCE_F_Chro1G0246400.2 | PCE_Fruticosa_Chro1 | 18456553 | 18461697 | RLP |
| PCE_F_Chro1G0246400.3 | PCE_Fruticosa_Chro1 | 18456553 | 18461697 | RLP |
| PCE_F_Chro1G0248900.1 | PCE_Fruticosa_Chro1 | 18650148 | 18652573 | RLP |
| PCE_F_Chro1G0253100.1 | PCE_Fruticosa_Chro1 | 19108146 | 19111328 | RLK |
| PCE_F_Chro1G0253100.2 | PCE_Fruticosa_Chro1 | 19108146 | 19111328 | RLK |
| PCE_F_Chro1G0263200.1 | PCE_Fruticosa_Chro1 | 19847806 | 19851593 | RLK |
| PCE_F_Chro1G0280800.1 | PCE_Fruticosa_Chro1 | 21114463 | 21117740 | RLK |
| PCE_F_Chro1G0280800.2 | PCE_Fruticosa_Chro1 | 21114463 | 21117740 | RLK |
| PCE_F_Chro1G0285500.1 | PCE_Fruticosa_Chro1 | 21556549 | 21558487 | SPTMK |
| PCE_F_Chro1G0286600.5 | PCE_Fruticosa_Chro1 | 21680346 | 21686556 | TIR-NB-LRR |
| PCE_F_Chro1G0286600.1 | PCE_Fruticosa_Chro1 | 21680346 | 21686556 | TIR-NB-LRR |
| PCE_F_Chro1G0286600.2 | PCE_Fruticosa_Chro1 | 21680346 | 21686556 | TIR-NB-LRR |
| PCE_F_Chro1G0286600.4 | PCE_Fruticosa_Chro1 | 21680346 | 21686556 | TIR-NB-LRR |
| PCE_F_Chro1G0286600.8 | PCE_Fruticosa_Chro1 | 21680346 | 21686556 | TIR-NB-LRR |
| PCE_F_Chro1G0286800.1 | PCE_Fruticosa_Chro1 | 21717345 | 21722975 | TIR-NB-LRR |
| PCE_F_Chro1G0286800.2 | PCE_Fruticosa_Chro1 | 21717345 | 21722975 | TIR-NB-LRR |
| PCE_F_Chro1G0286900.1 | PCE_Fruticosa_Chro1 | 21723321 | 21734051 | TIR-NB-LRR |
| PCE_F_Chro1G0287300.1 | PCE_Fruticosa_Chro1 | 21794971 | 21797280 | RLK |
| PCE_F_Chro1G0287300.2 | PCE_Fruticosa_Chro1 | 21794971 | 21797280 | RLK |
| PCE_F_Chro1G0293500.1 | PCE_Fruticosa_Chro1 | 22263019 | 22268301 | RLK |
| PCE_F_Chro1G0293500.4 | PCE_Fruticosa_Chro1 | 22263019 | 22268301 | RLK |
| PCE_F_Chro1G0293500.5 | PCE_Fruticosa_Chro1 | 22263019 | 22268301 | RLK |
| PCE_F_Chro1G0293500.9 | PCE_Fruticosa_Chro1 | 22263019 | 22268301 | RLK |
| PCE_F_Chro1G0293500.10 | PCE_Fruticosa_Chro1 | 22263019 | 22268301 | RLK |
| PCE_F_Chro1G0293500.12 | PCE_Fruticosa_Chro1 | 22263019 | 22268301 | RLK |
| PCE_F_Chro1G0305300.1 | PCE_Fruticosa_Chro1 | 23316129 | 23321329 | RLK |
| PCE_F_Chro1G0305300.2 | PCE_Fruticosa_Chro1 | 23316129 | 23321329 | RLK |
| PCE_F_Chro1G0308000.1 | PCE_Fruticosa_Chro1 | 23609503 | 23612116 | SPTMK |
| PCE_F_Chro1G0313300.1 | PCE_Fruticosa_Chro1 | 24151653 | 24155516 | RLK |
| PCE_F_Chro1G0327700.1 | PCE_Fruticosa_Chro1 | 25721785 | 25725367 | SPTMK |
| PCE_F_Chro1G0328000.1 | PCE_Fruticosa_Chro1 | 25775530 | 25777355 | SPTMK |
| PCE_F_Chro1G0328000.2 | PCE_Fruticosa_Chro1 | 25775530 | 25777355 | SPTMK |
| PCE_F_Chro1G0338500.2 | PCE_Fruticosa_Chro1 | 27211204 | 27216303 | SPTMK |
| PCE_F_Chro1G0343200.1 | PCE_Fruticosa_Chro1 | 28525576 | 28527652 | CC-NB-LRR |
| PCE_F_Chro1G0359200.2 | PCE_Fruticosa_Chro1 | 31854021 | 31868511 | CC-NB-LRR |
| PCE_F_Chro1G0359200.1 | PCE_Fruticosa_Chro1 | 31860986 | 31868511 | CC-NB-LRR |
| PCE_F_Chro1G0368700.1 | PCE_Fruticosa_Chro1 | 32993237 | 32996504 | SPTMK |
| PCE_F_Chro1G0368700.2 | PCE_Fruticosa_Chro1 | 32993237 | 32996531 | SPTMK |
| PCE_F_Chro1G0368700.3 | PCE_Fruticosa_Chro1 | 32993237 | 32996504 | SPTMK |
| PCE_F_Chro1G0368700.5 | PCE_Fruticosa_Chro1 | 32993237 | 32996504 | SPTMK |
| PCE_F_Chro1G0369000.1 | PCE_Fruticosa_Chro1 | 33057691 | 33060245 | SPTMK |
| PCE_F_Chro1G0381900.1 | PCE_Fruticosa_Chro1 | 35524617 | 35528640 | RLK |
| PCE_F_Chro1G0381900.2 | PCE_Fruticosa_Chro1 | 35524617 | 35528640 | RLK |
| PCE_F_Chro1G0391900.1 | PCE_Fruticosa_Chro1 | 36931145 | 36934709 | RLK |
| PCE_F_Chro1G0391900.4 | PCE_Fruticosa_Chro1 | 36931145 | 36934709 | RLK |
| PCE_F_Chro1G0392100.1 | PCE_Fruticosa_Chro1 | 36942239 | 36947253 | RLK |
| PCE_F_Chro1G0403000.1 | PCE_Fruticosa_Chro1 | 38315682 | 38319821 | TIR-NB-LRR |
| PCE_F_Chro1G0403000.2 | PCE_Fruticosa_Chro1 | 38315682 | 38319821 | TIR-NB-LRR |
| PCE_F_Chro1G0422100.1 | PCE_Fruticosa_Chro1 | 40318149 | 40321502 | RLK |
| PCE_F_Chro1G0456400.1 | PCE_Fruticosa_Chro1 | 43565046 | 43567926 | SPTMK |
| PCE_F_Chro1G0473800.1 | PCE_Fruticosa_Chro1 | 45166668 | 45171655 | RLK |
| PCE_F_Chro1G0485000.1 | PCE_Fruticosa_Chro1 | 46571995 | 46575601 | RLK |
| PCE_F_Chro1G0485000.2 | PCE_Fruticosa_Chro1 | 46571995 | 46575508 | RLK |
| PCE_F_Chro1G0499600.1 | PCE_Fruticosa_Chro1 | 48078457 | 48081939 | RLK |
| PCE_F_Chro1G0500300.1 | PCE_Fruticosa_Chro1 | 48191541 | 48197063 | TIR-NB-LRR |
| PCE_F_Chro1G0506800.1 | PCE_Fruticosa_Chro1 | 48959928 | 48963548 | RLK |
| PCE_F_Chro1G0506800.2 | PCE_Fruticosa_Chro1 | 48959937 | 48963548 | RLK |
| PCE_F_Chro1G0508500.4 | PCE_Fruticosa_Chro1 | 49038791 | 49044562 | RLK |
| PCE_F_Chro1G0508500.1 | PCE_Fruticosa_Chro1 | 49038898 | 49044562 | RLK |
| PCE_F_Chro1G0508500.2 | PCE_Fruticosa_Chro1 | 49038898 | 49044562 | RLK |
| PCE_F_Chro1G0508500.3 | PCE_Fruticosa_Chro1 | 49038898 | 49044562 | RLK |
| PCE_F_Chro1G0512100.1 | PCE_Fruticosa_Chro1 | 49392183 | 49394960 | CC-NB-LRR |
| PCE_F_Chro1G0514400.1 | PCE_Fruticosa_Chro1 | 49565794 | 49569096 | CC-NB-LRR |
| PCE_F_Chro1G0514400.2 | PCE_Fruticosa_Chro1 | 49565794 | 49569096 | CC-NB-LRR |
| PCE_F_Chro1G0514700.1 | PCE_Fruticosa_Chro1 | 49583706 | 49587080 | CC-NB-LRR |
| PCE_F_Chro1G0514700.2 | PCE_Fruticosa_Chro1 | 49583706 | 49587080 | CC-NB-LRR |
| PCE_F_Chro1G0515800.2 | PCE_Fruticosa_Chro1 | 49625252 | 49629539 | CC-NB-LRR |
| PCE_F_Chro1G0515800.3 | PCE_Fruticosa_Chro1 | 49625252 | 49629539 | CC-NB-LRR |
| PCE_F_Chro1G0515800.1 | PCE_Fruticosa_Chro1 | 49626103 | 49629539 | CC-NB-LRR |
| PCE_F_Chro1G0521100.1 | PCE_Fruticosa_Chro1 | 50039341 | 50043729 | CC-NB-LRR |
| PCE_F_Chro1G0521500.1 | PCE_Fruticosa_Chro1 | 50055225 | 50060407 | CC-NB-LRR |
| PCE_F_Chro1G0522900.2 | PCE_Fruticosa_Chro1 | 50135926 | 50139645 | CC-NB-LRR |
| PCE_F_Chro1G0522900.1 | PCE_Fruticosa_Chro1 | 50136300 | 50139645 | CC-NB-LRR |
| PCE_F_Chro1G0522900.3 | PCE_Fruticosa_Chro1 | 50136300 | 50139645 | CC-NB-LRR |
| PCE_F_Chro1G0523200.1 | PCE_Fruticosa_Chro1 | 50148323 | 50151873 | CC-NB-LRR |
| PCE_F_Chro1G0524100.1 | PCE_Fruticosa_Chro1 | 50219793 | 50228359 | CC-NB-LRR |
| PCE_F_Chro1G0524100.2 | PCE_Fruticosa_Chro1 | 50219793 | 50228359 | CC-NB-LRR |
| PCE_F_Chro1G0527600.1 | PCE_Fruticosa_Chro1 | 50433409 | 50438457 | CC-NB-LRR |
| PCE_F_Chro1G0527600.2 | PCE_Fruticosa_Chro1 | 50433409 | 50438457 | CC-NB-LRR |
| PCE_F_Chro1G0528500.1 | PCE_Fruticosa_Chro1 | 50580554 | 50592130 | CC-NB-LRR |
| PCE_F_Chro1G0538500.2 | PCE_Fruticosa_Chro1 | 51532653 | 51536880 | SPTMK |
| PCE_F_Chro1G0538500.1 | PCE_Fruticosa_Chro1 | 51533457 | 51536880 | SPTMK |
| PCE_F_Chro1G0546700.1 | PCE_Fruticosa_Chro1 | 52142321 | 52149585 | TIR-NB-LRR |
| PCE_F_Chro1G0553600.1 | PCE_Fruticosa_Chro1 | 52561315 | 52568065 | CC-NB-LRR |
| PCE_F_Chro2G0003800.1 | PCE_Fruticosa_Chro2 | 409758 | 412324 | SPTMK |
| PCE_F_Chro2G0003900.1 | PCE_Fruticosa_Chro2 | 421508 | 424122 | SPTMK |
| PCE_F_Chro2G0003900.3 | PCE_Fruticosa_Chro2 | 421508 | 423831 | SPTMK |
| PCE_F_Chro2G0007200.1 | PCE_Fruticosa_Chro2 | 687053 | 689476 | SPTMK |
| PCE_F_Chro2G0015100.1 | PCE_Fruticosa_Chro2 | 1597297 | 1600593 | SPTMK |
| PCE_F_Chro2G0015100.2 | PCE_Fruticosa_Chro2 | 1597297 | 1600593 | SPTMK |
| PCE_F_Chro2G0018400.1 | PCE_Fruticosa_Chro2 | 2108633 | 2111323 | CC-NB-LRR |
| PCE_F_Chro2G0026400.1 | PCE_Fruticosa_Chro2 | 3384833 | 3393466 | RLP |
| PCE_F_Chro2G0026400.2 | PCE_Fruticosa_Chro2 | 3384833 | 3393430 | RLP |
| PCE_F_Chro2G0026600.1 | PCE_Fruticosa_Chro2 | 3407871 | 3411014 | RLP |
| PCE_F_Chro2G0026700.1 | PCE_Fruticosa_Chro2 | 3415221 | 3418439 | RLP |
| PCE_F_Chro2G0026800.1 | PCE_Fruticosa_Chro2 | 3427870 | 3431010 | RLP |
| PCE_F_Chro2G0027600.1 | PCE_Fruticosa_Chro2 | 3534320 | 3545947 | CC-NB-LRR |
| PCE_F_Chro2G0028000.1 | PCE_Fruticosa_Chro2 | 3571841 | 3578598 | CC-NB-LRR |
| PCE_F_Chro2G0028100.1 | PCE_Fruticosa_Chro2 | 3610075 | 3616440 | CC-NB-LRR |
| PCE_F_Chro2G0028600.1 | PCE_Fruticosa_Chro2 | 3653007 | 3657878 | CC-NB-LRR |
| PCE_F_Chro2G0028700.1 | PCE_Fruticosa_Chro2 | 3699216 | 3705440 | CC-NB-LRR |
| PCE_F_Chro2G0029100.1 | PCE_Fruticosa_Chro2 | 3726857 | 3732794 | CC-NB-LRR |
| PCE_F_Chro2G0029400.1 | PCE_Fruticosa_Chro2 | 3778497 | 3783893 | CC-NB-LRR |
| PCE_F_Chro2G0031000.1 | PCE_Fruticosa_Chro2 | 4114548 | 4118027 | RLK |
| PCE_F_Chro2G0031000.4 | PCE_Fruticosa_Chro2 | 4114548 | 4118027 | RLK |
| PCE_F_Chro2G0032300.1 | PCE_Fruticosa_Chro2 | 4364884 | 4369212 | CC-NB-LRR |
| PCE_F_Chro2G0033600.1 | PCE_Fruticosa_Chro2 | 4498877 | 4505959 | CC-NB-LRR |
| PCE_F_Chro2G0033800.1 | PCE_Fruticosa_Chro2 | 4525870 | 4528593 | CC-NB-LRR |
| PCE_F_Chro2G0034000.1 | PCE_Fruticosa_Chro2 | 4577650 | 4584054 | CC-NB-LRR |
| PCE_F_Chro2G0034600.1 | PCE_Fruticosa_Chro2 | 4655077 | 4659150 | CC-NB-LRR |
| PCE_F_Chro2G0034800.1 | PCE_Fruticosa_Chro2 | 4687056 | 4691807 | CC-NB-LRR |
| PCE_F_Chro2G0037500.1 | PCE_Fruticosa_Chro2 | 5234413 | 5239211 | CC-NB-LRR |
| PCE_F_Chro2G0043600.1 | PCE_Fruticosa_Chro2 | 6019362 | 6034608 | CC-NB-LRR |
| PCE_F_Chro2G0046800.1 | PCE_Fruticosa_Chro2 | 6571388 | 6576927 | CC-NB-LRR |
| PCE_F_Chro2G0048000.1 | PCE_Fruticosa_Chro2 | 6631083 | 6635997 | SPTMK |
| PCE_F_Chro2G0048000.3 | PCE_Fruticosa_Chro2 | 6631083 | 6635997 | SPTMK |
| PCE_F_Chro2G0048000.4 | PCE_Fruticosa_Chro2 | 6631083 | 6635997 | SPTMK |
| PCE_F_Chro2G0048900.1 | PCE_Fruticosa_Chro2 | 6750010 | 6755443 | CC-NB-LRR |
| PCE_F_Chro2G0054900.1 | PCE_Fruticosa_Chro2 | 7538979 | 7548880 | CC-NB-LRR |
| PCE_F_Chro2G0054900.2 | PCE_Fruticosa_Chro2 | 7538979 | 7548880 | CC-NB-LRR |
| PCE_F_Chro2G0060200.1 | PCE_Fruticosa_Chro2 | 8448656 | 8452627 | CC-NB-LRR |
| PCE_F_Chro2G0060300.1 | PCE_Fruticosa_Chro2 | 8463468 | 8467331 | CC-NB-LRR |
| PCE_F_Chro2G0060800.1 | PCE_Fruticosa_Chro2 | 8517527 | 8522180 | RLK |
| PCE_F_Chro2G0064200.1 | PCE_Fruticosa_Chro2 | 8963776 | 8970132 | TIR-NB-LRR |
| PCE_F_Chro2G0065600.1 | PCE_Fruticosa_Chro2 | 9243463 | 9252468 | CC-NB-LRR |
| PCE_F_Chro2G0065800.1 | PCE_Fruticosa_Chro2 | 9272748 | 9276011 | CC-NB-LRR |
| PCE_F_Chro2G0066200.1 | PCE_Fruticosa_Chro2 | 9344004 | 9353097 | CC-NB-LRR |
| PCE_F_Chro2G0066800.1 | PCE_Fruticosa_Chro2 | 9382528 | 9385304 | SPTMK |
| PCE_F_Chro2G0067100.1 | PCE_Fruticosa_Chro2 | 9435338 | 9437982 | SPTMK |
| PCE_F_Chro2G0067200.1 | PCE_Fruticosa_Chro2 | 9455581 | 9458004 | SPTMK |
| PCE_F_Chro2G0067300.1 | PCE_Fruticosa_Chro2 | 9488239 | 9490451 | SPTMK |
| PCE_F_Chro2G0067600.1 | PCE_Fruticosa_Chro2 | 9584946 | 9587467 | SPTMK |
| PCE_F_Chro2G0067600.2 | PCE_Fruticosa_Chro2 | 9584946 | 9587467 | SPTMK |
| PCE_F_Chro2G0068700.1 | PCE_Fruticosa_Chro2 | 9785011 | 9790902 | TIR-NB-LRR |
| PCE_F_Chro2G0069000.1 | PCE_Fruticosa_Chro2 | 9970358 | 9973706 | RLP |
| PCE_F_Chro2G0073300.1 | PCE_Fruticosa_Chro2 | 10695331 | 10699226 | CC-NB-LRR |
| PCE_F_Chro2G0073500.1 | PCE_Fruticosa_Chro2 | 10714189 | 10718134 | CC-NB-LRR |
| PCE_F_Chro2G0074900.1 | PCE_Fruticosa_Chro2 | 10984484 | 10989168 | TIR-NB-LRR |
| PCE_F_Chro2G0074900.2 | PCE_Fruticosa_Chro2 | 10984484 | 10989800 | TIR-NB-LRR |
| PCE_F_Chro2G0074900.3 | PCE_Fruticosa_Chro2 | 10984484 | 10989800 | TIR-NB-LRR |
| PCE_F_Chro2G0074900.4 | PCE_Fruticosa_Chro2 | 10984484 | 10989800 | TIR-NB-LRR |
| PCE_F_Chro2G0074900.5 | PCE_Fruticosa_Chro2 | 10984484 | 10989800 | TIR-NB-LRR |
| PCE_F_Chro2G0074900.6 | PCE_Fruticosa_Chro2 | 10984484 | 10989800 | TIR-NB-LRR |
| PCE_F_Chro2G0075100.1 | PCE_Fruticosa_Chro2 | 11035633 | 11040215 | TIR-NB-LRR |
| PCE_F_Chro2G0075100.2 | PCE_Fruticosa_Chro2 | 11035633 | 11042215 | TIR-NB-LRR |
| PCE_F_Chro2G0075100.3 | PCE_Fruticosa_Chro2 | 11035633 | 11042215 | TIR-NB-LRR |
| PCE_F_Chro2G0075100.4 | PCE_Fruticosa_Chro2 | 11035633 | 11042215 | TIR-NB-LRR |
| PCE_F_Chro2G0075100.5 | PCE_Fruticosa_Chro2 | 11035633 | 11040215 | TIR-NB-LRR |
| PCE_F_Chro2G0075100.6 | PCE_Fruticosa_Chro2 | 11035633 | 11042215 | TIR-NB-LRR |
| PCE_F_Chro2G0075100.7 | PCE_Fruticosa_Chro2 | 11035633 | 11042215 | TIR-NB-LRR |
| PCE_F_Chro2G0077100.2 | PCE_Fruticosa_Chro2 | 11227307 | 11233976 | TIR-NB-LRR |
| PCE_F_Chro2G0077100.1 | PCE_Fruticosa_Chro2 | 11228551 | 11233976 | TIR-NB-LRR |
| PCE_F_Chro2G0077300.1 | PCE_Fruticosa_Chro2 | 11271740 | 11276050 | TIR-NB-LRR |
| PCE_F_Chro2G0078500.1 | PCE_Fruticosa_Chro2 | 11451812 | 11456363 | CC-NB-LRR |
| PCE_F_Chro2G0079000.1 | PCE_Fruticosa_Chro2 | 11518719 | 11523556 | TIR-NB-LRR |
| PCE_F_Chro2G0082800.1 | PCE_Fruticosa_Chro2 | 12282312 | 12286661 | CC-NB-LRR |
| PCE_F_Chro2G0083000.1 | PCE_Fruticosa_Chro2 | 12296872 | 12301737 | CC-NB-LRR |
| PCE_F_Chro2G0083700.1 | PCE_Fruticosa_Chro2 | 12442610 | 12454548 | CC-NB-LRR |
| PCE_F_Chro2G0083700.2 | PCE_Fruticosa_Chro2 | 12450894 | 12454548 | CC-NB-LRR |
| PCE_F_Chro2G0088100.1 | PCE_Fruticosa_Chro2 | 13186543 | 13194921 | RLK |
| PCE_F_Chro2G0088100.2 | PCE_Fruticosa_Chro2 | 13186543 | 13194921 | RLK |
| PCE_F_Chro2G0088100.3 | PCE_Fruticosa_Chro2 | 13186543 | 13194921 | RLK |
| PCE_F_Chro2G0088100.4 | PCE_Fruticosa_Chro2 | 13188720 | 13194921 | RLK |
| PCE_F_Chro2G0093800.1 | PCE_Fruticosa_Chro2 | 14556624 | 14559263 | RLP |
| PCE_F_Chro2G0097800.1 | PCE_Fruticosa_Chro2 | 15938740 | 15945238 | RLK |
| PCE_F_Chro2G0097800.2 | PCE_Fruticosa_Chro2 | 15938740 | 15945238 | RLK |
| PCE_F_Chro2G0109300.1 | PCE_Fruticosa_Chro2 | 19054750 | 19061272 | RLP |
| PCE_F_Chro2G0109900.1 | PCE_Fruticosa_Chro2 | 19194869 | 19198690 | RLP |
| PCE_F_Chro2G0109900.2 | PCE_Fruticosa_Chro2 | 19194869 | 19198690 | RLP |
| PCE_F_Chro2G0109900.3 | PCE_Fruticosa_Chro2 | 19194869 | 19198690 | RLP |
| PCE_F_Chro2G0110200.1 | PCE_Fruticosa_Chro2 | 19278972 | 19282623 | RLP |
| PCE_F_Chro2G0119300.1 | PCE_Fruticosa_Chro2 | 20860408 | 20862524 | SPTMK |
| PCE_F_Chro2G0120800.1 | PCE_Fruticosa_Chro2 | 21093958 | 21096042 | SPTMK |
| PCE_F_Chro2G0120800.2 | PCE_Fruticosa_Chro2 | 21093958 | 21096042 | SPTMK |
| PCE_F_Chro2G0120900.1 | PCE_Fruticosa_Chro2 | 21103680 | 21105585 | SPTMK |
| PCE_F_Chro2G0121700.1 | PCE_Fruticosa_Chro2 | 21245396 | 21247503 | SPTMK |
| PCE_F_Chro2G0129800.1 | PCE_Fruticosa_Chro2 | 23918677 | 23923858 | SPTMK |
| PCE_F_Chro2G0129900.1 | PCE_Fruticosa_Chro2 | 23956558 | 23963035 | SPTMK |
| PCE_F_Chro2G0130700.1 | PCE_Fruticosa_Chro2 | 24116266 | 24118342 | SPTMK |
| PCE_F_Chro2G0130700.2 | PCE_Fruticosa_Chro2 | 24116266 | 24118342 | SPTMK |
| PCE_F_Chro2G0130700.3 | PCE_Fruticosa_Chro2 | 24116266 | 24118342 | SPTMK |
| PCE_F_Chro2G0131000.1 | PCE_Fruticosa_Chro2 | 24175929 | 24178016 | SPTMK |
| PCE_F_Chro2G0131000.2 | PCE_Fruticosa_Chro2 | 24175929 | 24178016 | SPTMK |
| PCE_F_Chro2G0131700.1 | PCE_Fruticosa_Chro2 | 24255622 | 24258307 | SPTMK |
| PCE_F_Chro2G0131700.2 | PCE_Fruticosa_Chro2 | 24255622 | 24258307 | SPTMK |
| PCE_F_Chro2G0131700.3 | PCE_Fruticosa_Chro2 | 24255622 | 24258307 | SPTMK |
| PCE_F_Chro2G0131700.4 | PCE_Fruticosa_Chro2 | 24255622 | 24258307 | SPTMK |
| PCE_F_Chro2G0131700.5 | PCE_Fruticosa_Chro2 | 24255622 | 24258307 | SPTMK |
| PCE_F_Chro2G0131900.1 | PCE_Fruticosa_Chro2 | 24314577 | 24316657 | SPTMK |
| PCE_F_Chro2G0131900.2 | PCE_Fruticosa_Chro2 | 24314577 | 24316657 | SPTMK |
| PCE_F_Chro2G0131900.3 | PCE_Fruticosa_Chro2 | 24314577 | 24316657 | SPTMK |
| PCE_F_Chro2G0132400.1 | PCE_Fruticosa_Chro2 | 24431574 | 24434080 | SPTMK |
| PCE_F_Chro2G0132400.2 | PCE_Fruticosa_Chro2 | 24431574 | 24434080 | SPTMK |
| PCE_F_Chro2G0136400.1 | PCE_Fruticosa_Chro2 | 25083479 | 25087446 | SPTMK |
| PCE_F_Chro2G0136500.1 | PCE_Fruticosa_Chro2 | 25093079 | 25104400 | SPTMK |
| PCE_F_Chro2G0136500.2 | PCE_Fruticosa_Chro2 | 25093079 | 25104400 | SPTMK |
| PCE_F_Chro2G0136900.1 | PCE_Fruticosa_Chro2 | 25113665 | 25116282 | SPTMK |
| PCE_F_Chro2G0136900.2 | PCE_Fruticosa_Chro2 | 25113665 | 25116282 | SPTMK |
| PCE_F_Chro2G0140800.1 | PCE_Fruticosa_Chro2 | 25518551 | 25520780 | RLP |
| PCE_F_Chro2G0140900.1 | PCE_Fruticosa_Chro2 | 25566386 | 25568509 | RLP |
| PCE_F_Chro2G0145000.1 | PCE_Fruticosa_Chro2 | 26130884 | 26135478 | TIR-NB-LRR |
| PCE_F_Chro2G0145400.1 | PCE_Fruticosa_Chro2 | 26159554 | 26163970 | TIR-NB-LRR |
| PCE_F_Chro2G0145400.2 | PCE_Fruticosa_Chro2 | 26159554 | 26163970 | TIR-NB-LRR |
| PCE_F_Chro2G0147400.1 | PCE_Fruticosa_Chro2 | 26341297 | 26343355 | SPTMK |
| PCE_F_Chro2G0149800.2 | PCE_Fruticosa_Chro2 | 26685797 | 26689587 | CC-NB-LRR |
| PCE_F_Chro2G0149800.1 | PCE_Fruticosa_Chro2 | 26686373 | 26689587 | CC-NB-LRR |
| PCE_F_Chro2G0158100.1 | PCE_Fruticosa_Chro2 | 27622375 | 27628747 | CC-NB-LRR |
| PCE_F_Chro2G0158100.2 | PCE_Fruticosa_Chro2 | 27622375 | 27628747 | CC-NB-LRR |
| PCE_F_Chro2G0158100.3 | PCE_Fruticosa_Chro2 | 27622375 | 27628747 | CC-NB-LRR |
| PCE_F_Chro2G0158100.4 | PCE_Fruticosa_Chro2 | 27622375 | 27628747 | CC-NB-LRR |
| PCE_F_Chro2G0162400.1 | PCE_Fruticosa_Chro2 | 28143640 | 28149491 | CC-NB-LRR |
| PCE_F_Chro2G0164900.1 | PCE_Fruticosa_Chro2 | 28339893 | 28343079 | RLK |
| PCE_F_Chro2G0164900.3 | PCE_Fruticosa_Chro2 | 28340397 | 28343079 | RLK |
| PCE_F_Chro2G0165400.1 | PCE_Fruticosa_Chro2 | 28374465 | 28377707 | RLK |
| PCE_F_Chro2G0166800.1 | PCE_Fruticosa_Chro2 | 28504441 | 28510883 | CC-NB-LRR |
| PCE_F_Chro2G0166800.2 | PCE_Fruticosa_Chro2 | 28504441 | 28510883 | CC-NB-LRR |
| PCE_F_Chro2G0177400.1 | PCE_Fruticosa_Chro2 | 29810275 | 29814769 | TIR-NB-LRR |
| PCE_F_Chro2G0177600.1 | PCE_Fruticosa_Chro2 | 29832865 | 29850560 | TIR-NB-LRR |
| PCE_F_Chro2G0177600.2 | PCE_Fruticosa_Chro2 | 29832865 | 29850734 | TIR-NB-LRR |
| PCE_F_Chro2G0177800.1 | PCE_Fruticosa_Chro2 | 29876494 | 29880959 | TIR-NB-LRR |
| PCE_F_Chro2G0179000.1 | PCE_Fruticosa_Chro2 | 29959880 | 29976914 | TIR-NB-LRR |
| PCE_F_Chro2G0180900.1 | PCE_Fruticosa_Chro2 | 30198574 | 30201788 | RLP |
| PCE_F_Chro2G0181100.1 | PCE_Fruticosa_Chro2 | 30214228 | 30217580 | RLP |
| PCE_F_Chro2G0182200.1 | PCE_Fruticosa_Chro2 | 30317474 | 30320311 | RLP |
| PCE_F_Chro2G0182300.1 | PCE_Fruticosa_Chro2 | 30344688 | 30347861 | RLP |
| PCE_F_Chro2G0182700.1 | PCE_Fruticosa_Chro2 | 30411246 | 30415163 | RLP |
| PCE_F_Chro2G0183000.1 | PCE_Fruticosa_Chro2 | 30543258 | 30546307 | RLP |
| PCE_F_Chro2G0183300.1 | PCE_Fruticosa_Chro2 | 30605920 | 30608808 | RLP |
| PCE_F_Chro2G0184100.1 | PCE_Fruticosa_Chro2 | 30737496 | 30740538 | RLP |
| PCE_F_Chro2G0184200.1 | PCE_Fruticosa_Chro2 | 30813248 | 30816064 | RLP |
| PCE_F_Chro2G0190400.2 | PCE_Fruticosa_Chro2 | 31464350 | 31467338 | RLP |
| PCE_F_Chro2G0190400.1 | PCE_Fruticosa_Chro2 | 31464350 | 31467338 | RLP |
| PCE_F_Chro2G0190500.1 | PCE_Fruticosa_Chro2 | 31494943 | 31498369 | RLP |
| PCE_F_Chro2G0190700.1 | PCE_Fruticosa_Chro2 | 31538145 | 31541021 | RLP |
| PCE_F_Chro2G0200200.1 | PCE_Fruticosa_Chro2 | 32311273 | 32316432 | RLP |
| PCE_F_Chro2G0202100.1 | PCE_Fruticosa_Chro2 | 32475679 | 32479492 | RLP |
| PCE_F_Chro2G0204000.1 | PCE_Fruticosa_Chro2 | 32628338 | 32636386 | RLP |
| PCE_F_Chro2G0208300.1 | PCE_Fruticosa_Chro2 | 32858488 | 32861309 | RLP |
| PCE_F_Chro2G0208300.2 | PCE_Fruticosa_Chro2 | 32858503 | 32861309 | RLP |
| PCE_F_Chro2G0208500.1 | PCE_Fruticosa_Chro2 | 32870317 | 32873171 | RLP |
| PCE_F_Chro2G0210800.1 | PCE_Fruticosa_Chro2 | 33070943 | 33074374 | RLP |
| PCE_F_Chro2G0210800.2 | PCE_Fruticosa_Chro2 | 33070943 | 33074374 | RLP |
| PCE_F_Chro2G0212400.1 | PCE_Fruticosa_Chro2 | 33176911 | 33180265 | SPTMK |
| PCE_F_Chro2G0232500.1 | PCE_Fruticosa_Chro2 | 34669758 | 34672527 | SPTMK |
| PCE_F_Chro2G0236000.1 | PCE_Fruticosa_Chro2 | 34899889 | 34903126 | RLK |
| PCE_F_Chro2G0240800.1 | PCE_Fruticosa_Chro2 | 35270469 | 35273237 | SPTMK |
| PCE_F_Chro2G0266800.1 | PCE_Fruticosa_Chro2 | 37204609 | 37208188 | SPTMK |
| PCE_F_Chro2G0279200.1 | PCE_Fruticosa_Chro2 | 38031481 | 38034094 | SPTMK |
| PCE_F_Chro2G0280700.1 | PCE_Fruticosa_Chro2 | 38125421 | 38128015 | SPTMK |
| PCE_F_Chro2G0280700.2 | PCE_Fruticosa_Chro2 | 38125421 | 38128015 | SPTMK |
| PCE_F_Chro2G0288400.1 | PCE_Fruticosa_Chro2 | 38613380 | 38616607 | RLK |
| PCE_F_Chro2G0288400.2 | PCE_Fruticosa_Chro2 | 38613380 | 38616607 | RLK |
| PCE_F_Chro2G0288500.1 | PCE_Fruticosa_Chro2 | 38618479 | 38621794 | RLK |
| PCE_F_Chro2G0288500.2 | PCE_Fruticosa_Chro2 | 38618479 | 38621764 | RLK |
| PCE_F_Chro2G0290600.1 | PCE_Fruticosa_Chro2 | 38730671 | 38733106 | SPTMK |
| PCE_F_Chro2G0294200.1 | PCE_Fruticosa_Chro2 | 38974797 | 38978989 | RLK |
| PCE_F_Chro2G0300300.1 | PCE_Fruticosa_Chro2 | 39340899 | 39344049 | RLK |
| PCE_F_Chro2G0300300.2 | PCE_Fruticosa_Chro2 | 39340899 | 39344049 | RLK |
| PCE_F_Chro2G0307200.1 | PCE_Fruticosa_Chro2 | 39718525 | 39721538 | RLK |
| PCE_F_Chro2G0308100.1 | PCE_Fruticosa_Chro2 | 39772759 | 39775597 | RLP |
| PCE_F_Chro2G0308200.2 | PCE_Fruticosa_Chro2 | 39776553 | 39790757 | RLP |
| PCE_F_Chro2G0308200.1 | PCE_Fruticosa_Chro2 | 39776565 | 39790757 | RLP |
| PCE_F_Chro2G0312600.1 | PCE_Fruticosa_Chro2 | 40028637 | 40032014 | CC-NB-LRR |
| PCE_F_Chro2G0313500.1 | PCE_Fruticosa_Chro2 | 40087897 | 40092168 | RLK |
| PCE_F_Chro2G0317200.1 | PCE_Fruticosa_Chro2 | 40329667 | 40332910 | RLK |
| PCE_F_Chro2G0319100.1 | PCE_Fruticosa_Chro2 | 40427495 | 40430979 | RLK |
| PCE_F_Chro2G0319100.2 | PCE_Fruticosa_Chro2 | 40427495 | 40430007 | RLK |
| PCE_F_Chro2G0320800.1 | PCE_Fruticosa_Chro2 | 40516900 | 40521073 | CC-NB-LRR |
| PCE_F_Chro2G0320900.1 | PCE_Fruticosa_Chro2 | 40522372 | 40525191 | CC-NB-LRR |
| PCE_F_Chro2G0328500.1 | PCE_Fruticosa_Chro2 | 40969886 | 40973903 | RLK |
| PCE_F_Chro2G0339200.1 | PCE_Fruticosa_Chro2 | 41534199 | 41537082 | SPTMK |
| PCE_F_Chro2G0344100.1 | PCE_Fruticosa_Chro2 | 41802676 | 41806110 | SPTMK |
| PCE_F_Chro2G0344300.1 | PCE_Fruticosa_Chro2 | 41814890 | 41817776 | RLP |
| PCE_F_Chro2G0345900.1 | PCE_Fruticosa_Chro2 | 41906844 | 41910308 | RLK |
| PCE_F_Chro2G0366000.1 | PCE_Fruticosa_Chro2 | 43072629 | 43074886 | RLP |
| PCE_F_Chro3G0013500.3 | PCE_Fruticosa_Chro3 | 1009687 | 1015576 | TIR-NB-LRR |
| PCE_F_Chro3G0013500.1 | PCE_Fruticosa_Chro3 | 1010618 | 1015576 | TIR-NB-LRR |
| PCE_F_Chro3G0014200.1 | PCE_Fruticosa_Chro3 | 1056893 | 1062661 | SPTMK |
| PCE_F_Chro3G0015100.1 | PCE_Fruticosa_Chro3 | 1128457 | 1133187 | SPTMK |
| PCE_F_Chro3G0015100.2 | PCE_Fruticosa_Chro3 | 1128457 | 1133187 | SPTMK |
| PCE_F_Chro3G0015600.1 | PCE_Fruticosa_Chro3 | 1211500 | 1219188 | SPTMK |
| PCE_F_Chro3G0018300.1 | PCE_Fruticosa_Chro3 | 1491921 | 1497685 | SPTMK |
| PCE_F_Chro3G0018300.2 | PCE_Fruticosa_Chro3 | 1491921 | 1497685 | SPTMK |
| PCE_F_Chro3G0018300.3 | PCE_Fruticosa_Chro3 | 1491921 | 1497685 | SPTMK |
| PCE_F_Chro3G0018300.4 | PCE_Fruticosa_Chro3 | 1491921 | 1497685 | SPTMK |
| PCE_F_Chro3G0018300.5 | PCE_Fruticosa_Chro3 | 1491921 | 1497685 | SPTMK |
| PCE_F_Chro3G0019200.1 | PCE_Fruticosa_Chro3 | 1565497 | 1569932 | SPTMK |
| PCE_F_Chro3G0019500.1 | PCE_Fruticosa_Chro3 | 1605461 | 1611919 | SPTMK |
| PCE_F_Chro3G0024400.1 | PCE_Fruticosa_Chro3 | 2104598 | 2112655 | RLK |
| PCE_F_Chro3G0024400.2 | PCE_Fruticosa_Chro3 | 2104598 | 2112655 | RLK |
| PCE_F_Chro3G0028400.1 | PCE_Fruticosa_Chro3 | 2466347 | 2470128 | SPTMK |
| PCE_F_Chro3G0028400.2 | PCE_Fruticosa_Chro3 | 2466347 | 2470128 | SPTMK |
| PCE_F_Chro3G0028400.3 | PCE_Fruticosa_Chro3 | 2466347 | 2469058 | SPTMK |
| PCE_F_Chro3G0028400.4 | PCE_Fruticosa_Chro3 | 2466347 | 2470128 | SPTMK |
| PCE_F_Chro3G0028400.5 | PCE_Fruticosa_Chro3 | 2466347 | 2470128 | SPTMK |
| PCE_F_Chro3G0030100.1 | PCE_Fruticosa_Chro3 | 2573483 | 2575711 | RLP |
| PCE_F_Chro3G0030400.1 | PCE_Fruticosa_Chro3 | 2612315 | 2614623 | RLP |
| PCE_F_Chro3G0030500.1 | PCE_Fruticosa_Chro3 | 2622135 | 2624456 | RLP |
| PCE_F_Chro3G0031500.1 | PCE_Fruticosa_Chro3 | 2709228 | 2711623 | RLK |
| PCE_F_Chro3G0031500.2 | PCE_Fruticosa_Chro3 | 2709228 | 2711617 | RLK |
| PCE_F_Chro3G0031500.3 | PCE_Fruticosa_Chro3 | 2709228 | 2711605 | RLK |
| PCE_F_Chro3G0031500.4 | PCE_Fruticosa_Chro3 | 2709228 | 2711587 | RLK |
| PCE_F_Chro3G0031500.5 | PCE_Fruticosa_Chro3 | 2709228 | 2711599 | RLK |
| PCE_F_Chro3G0032500.1 | PCE_Fruticosa_Chro3 | 2822741 | 2828169 | RLK |
| PCE_F_Chro3G0032500.2 | PCE_Fruticosa_Chro3 | 2822741 | 2828169 | RLK |
| PCE_F_Chro3G0033300.1 | PCE_Fruticosa_Chro3 | 2912665 | 2915007 | RLP |
| PCE_F_Chro3G0033400.1 | PCE_Fruticosa_Chro3 | 2924282 | 2926576 | RLP |
| PCE_F_Chro3G0034300.1 | PCE_Fruticosa_Chro3 | 3009643 | 3011992 | RLK |
| PCE_F_Chro3G0034300.2 | PCE_Fruticosa_Chro3 | 3009643 | 3011986 | RLK |
| PCE_F_Chro3G0034300.3 | PCE_Fruticosa_Chro3 | 3009643 | 3011974 | RLK |
| PCE_F_Chro3G0034300.4 | PCE_Fruticosa_Chro3 | 3009643 | 3011956 | RLK |
| PCE_F_Chro3G0034300.5 | PCE_Fruticosa_Chro3 | 3009643 | 3011968 | RLK |
| PCE_F_Chro3G0035500.1 | PCE_Fruticosa_Chro3 | 3178925 | 3184608 | RLK |
| PCE_F_Chro3G0037500.1 | PCE_Fruticosa_Chro3 | 3455961 | 3460215 | RLK |
| PCE_F_Chro3G0038100.1 | PCE_Fruticosa_Chro3 | 3538088 | 3545499 | RLK |
| PCE_F_Chro3G0046100.1 | PCE_Fruticosa_Chro3 | 4128197 | 4130408 | RLK |
| PCE_F_Chro3G0056700.1 | PCE_Fruticosa_Chro3 | 5031263 | 5036289 | SPTMK |
| PCE_F_Chro3G0056700.2 | PCE_Fruticosa_Chro3 | 5031263 | 5036289 | SPTMK |
| PCE_F_Chro3G0062200.1 | PCE_Fruticosa_Chro3 | 5546275 | 5550133 | RLK |
| PCE_F_Chro3G0062200.2 | PCE_Fruticosa_Chro3 | 5546275 | 5550127 | RLK |
| PCE_F_Chro3G0063400.1 | PCE_Fruticosa_Chro3 | 5657075 | 5691219 | RLK |
| PCE_F_Chro3G0063400.2 | PCE_Fruticosa_Chro3 | 5657075 | 5691219 | RLK |
| PCE_F_Chro3G0063600.3 | PCE_Fruticosa_Chro3 | 5732909 | 5745570 | RLK |
| PCE_F_Chro3G0063600.1 | PCE_Fruticosa_Chro3 | 5739387 | 5745570 | RLK |
| PCE_F_Chro3G0063600.2 | PCE_Fruticosa_Chro3 | 5739387 | 5745570 | RLK |
| PCE_F_Chro3G0063900.1 | PCE_Fruticosa_Chro3 | 5796127 | 5800221 | RLK |
| PCE_F_Chro3G0064000.1 | PCE_Fruticosa_Chro3 | 5811428 | 5817535 | RLK |
| PCE_F_Chro3G0064000.2 | PCE_Fruticosa_Chro3 | 5811428 | 5817535 | RLK |
| PCE_F_Chro3G0064000.3 | PCE_Fruticosa_Chro3 | 5811428 | 5817535 | RLK |
| PCE_F_Chro3G0066900.2 | PCE_Fruticosa_Chro3 | 6035939 | 6041909 | LysM |
| PCE_F_Chro3G0066900.4 | PCE_Fruticosa_Chro3 | 6035939 | 6041909 | LysM |
| PCE_F_Chro3G0066900.1 | PCE_Fruticosa_Chro3 | 6035939 | 6041909 | LysM |
| PCE_F_Chro3G0066900.3 | PCE_Fruticosa_Chro3 | 6035939 | 6041909 | LysM |
| PCE_F_Chro3G0066900.5 | PCE_Fruticosa_Chro3 | 6035939 | 6041227 | LysM |
| PCE_F_Chro3G0066900.6 | PCE_Fruticosa_Chro3 | 6035939 | 6041909 | LysM |
| PCE_F_Chro3G0069000.1 | PCE_Fruticosa_Chro3 | 6259266 | 6266473 | RLK |
| PCE_F_Chro3G0069000.2 | PCE_Fruticosa_Chro3 | 6259266 | 6266473 | RLK |
| PCE_F_Chro3G0069000.3 | PCE_Fruticosa_Chro3 | 6259266 | 6266473 | RLK |
| PCE_F_Chro3G0069000.5 | PCE_Fruticosa_Chro3 | 6259266 | 6266473 | RLK |
| PCE_F_Chro3G0069000.6 | PCE_Fruticosa_Chro3 | 6259266 | 6266473 | RLK |
| PCE_F_Chro3G0069000.7 | PCE_Fruticosa_Chro3 | 6259266 | 6266473 | RLK |
| PCE_F_Chro3G0069000.8 | PCE_Fruticosa_Chro3 | 6259266 | 6266473 | RLK |
| PCE_F_Chro3G0069000.9 | PCE_Fruticosa_Chro3 | 6259266 | 6266473 | RLK |
| PCE_F_Chro3G0079700.1 | PCE_Fruticosa_Chro3 | 7315180 | 7317546 | SPTMK |
| PCE_F_Chro3G0093300.1 | PCE_Fruticosa_Chro3 | 8556733 | 8560277 | CC-NB-LRR |
| PCE_F_Chro3G0096600.1 | PCE_Fruticosa_Chro3 | 8838579 | 8841977 | SPTMK |
| PCE_F_Chro3G0100100.1 | PCE_Fruticosa_Chro3 | 9676702 | 9682310 | CC-NB-LRR |
| PCE_F_Chro3G0100200.1 | PCE_Fruticosa_Chro3 | 9686513 | 9690935 | CC-NB-LRR |
| PCE_F_Chro3G0100400.1 | PCE_Fruticosa_Chro3 | 9706051 | 9715207 | CC-NB-LRR |
| PCE_F_Chro3G0100400.3 | PCE_Fruticosa_Chro3 | 9706948 | 9715207 | CC-NB-LRR |
| PCE_F_Chro3G0100400.2 | PCE_Fruticosa_Chro3 | 9707442 | 9715207 | CC-NB-LRR |
| PCE_F_Chro3G0104200.1 | PCE_Fruticosa_Chro3 | 10084540 | 10087524 | CC-NB-LRR |
| PCE_F_Chro3G0108500.1 | PCE_Fruticosa_Chro3 | 10575821 | 10580102 | SPTMK |
| PCE_F_Chro3G0108500.2 | PCE_Fruticosa_Chro3 | 10575821 | 10580102 | SPTMK |
| PCE_F_Chro3G0109400.1 | PCE_Fruticosa_Chro3 | 10743859 | 10748092 | SPTMK |
| PCE_F_Chro3G0109400.2 | PCE_Fruticosa_Chro3 | 10743859 | 10748092 | SPTMK |
| PCE_F_Chro3G0126000.1 | PCE_Fruticosa_Chro3 | 12652123 | 12653546 | LysM |
| PCE_F_Chro3G0131500.1 | PCE_Fruticosa_Chro3 | 13373519 | 13374919 | LysM |
| PCE_F_Chro3G0151700.1 | PCE_Fruticosa_Chro3 | 16860623 | 16864963 | SPTMK |
| PCE_F_Chro3G0155400.1 | PCE_Fruticosa_Chro3 | 18446407 | 18449100 | SPTMK |
| PCE_F_Chro3G0156200.1 | PCE_Fruticosa_Chro3 | 18638395 | 18642837 | SPTMK |
| PCE_F_Chro3G0159500.1 | PCE_Fruticosa_Chro3 | 19253210 | 19257828 | SPTMK |
| PCE_F_Chro3G0162100.1 | PCE_Fruticosa_Chro3 | 19721209 | 19724082 | SPTMK |
| PCE_F_Chro3G0166900.1 | PCE_Fruticosa_Chro3 | 20705103 | 20711007 | RLK |
| PCE_F_Chro3G0168700.2 | PCE_Fruticosa_Chro3 | 20846940 | 20852913 | SPTMK |
| PCE_F_Chro3G0168700.1 | PCE_Fruticosa_Chro3 | 20849333 | 20852913 | SPTMK |
| PCE_F_Chro3G0168700.3 | PCE_Fruticosa_Chro3 | 20849333 | 20852913 | SPTMK |
| PCE_F_Chro3G0168700.4 | PCE_Fruticosa_Chro3 | 20849333 | 20853351 | SPTMK |
| PCE_F_Chro3G0169000.1 | PCE_Fruticosa_Chro3 | 20914077 | 20916946 | SPTMK |
| PCE_F_Chro3G0169000.3 | PCE_Fruticosa_Chro3 | 20914077 | 20916946 | SPTMK |
| PCE_F_Chro3G0183300.1 | PCE_Fruticosa_Chro3 | 23015880 | 23019772 | SPTMK |
| PCE_F_Chro3G0183300.2 | PCE_Fruticosa_Chro3 | 23015880 | 23019772 | SPTMK |
| PCE_F_Chro3G0183600.4 | PCE_Fruticosa_Chro3 | 23056054 | 23059970 | SPTMK |
| PCE_F_Chro3G0183600.1 | PCE_Fruticosa_Chro3 | 23056500 | 23059970 | SPTMK |
| PCE_F_Chro3G0183600.2 | PCE_Fruticosa_Chro3 | 23056500 | 23062371 | SPTMK |
| PCE_F_Chro3G0183600.3 | PCE_Fruticosa_Chro3 | 23056500 | 23059970 | SPTMK |
| PCE_F_Chro3G0185300.1 | PCE_Fruticosa_Chro3 | 23218554 | 23221594 | RLK |
| PCE_F_Chro3G0196700.1 | PCE_Fruticosa_Chro3 | 24570917 | 24575213 | RLK |
| PCE_F_Chro3G0201600.1 | PCE_Fruticosa_Chro3 | 25064065 | 25068436 | RLK |
| PCE_F_Chro3G0201600.2 | PCE_Fruticosa_Chro3 | 25064065 | 25068436 | RLK |
| PCE_F_Chro3G0204700.1 | PCE_Fruticosa_Chro3 | 25381422 | 25384968 | RLK |
| PCE_F_Chro3G0205300.1 | PCE_Fruticosa_Chro3 | 25436232 | 25444054 | RLK |
| PCE_F_Chro3G0205300.2 | PCE_Fruticosa_Chro3 | 25436232 | 25444054 | RLK |
| PCE_F_Chro3G0205300.3 | PCE_Fruticosa_Chro3 | 25436232 | 25444054 | RLK |
| PCE_F_Chro3G0205300.5 | PCE_Fruticosa_Chro3 | 25436232 | 25444054 | RLK |
| PCE_F_Chro3G0205600.1 | PCE_Fruticosa_Chro3 | 25471946 | 25473849 | RLP |
| PCE_F_Chro3G0207700.2 | PCE_Fruticosa_Chro3 | 25698022 | 25701583 | RLK |
| PCE_F_Chro3G0210200.1 | PCE_Fruticosa_Chro3 | 26064332 | 26067515 | RLK |
| PCE_F_Chro3G0228800.1 | PCE_Fruticosa_Chro3 | 27560501 | 27567818 | RLP |
| PCE_F_Chro3G0229300.1 | PCE_Fruticosa_Chro3 | 27599566 | 27602883 | RLP |
| PCE_F_Chro3G0232100.1 | PCE_Fruticosa_Chro3 | 27762740 | 27765921 | RLP |
| PCE_F_Chro3G0236400.1 | PCE_Fruticosa_Chro3 | 28075771 | 28078549 | SPTMK |
| PCE_F_Chro3G0240000.1 | PCE_Fruticosa_Chro3 | 28284696 | 28287236 | SPTMK |
| PCE_F_Chro3G0240200.1 | PCE_Fruticosa_Chro3 | 28329976 | 28334004 | SPTMK |
| PCE_F_Chro3G0240200.3 | PCE_Fruticosa_Chro3 | 28329976 | 28334004 | SPTMK |
| PCE_F_Chro3G0241200.1 | PCE_Fruticosa_Chro3 | 28520350 | 28524623 | SPTMK |
| PCE_F_Chro3G0241400.1 | PCE_Fruticosa_Chro3 | 28535521 | 28539440 | SPTMK |
| PCE_F_Chro3G0241500.1 | PCE_Fruticosa_Chro3 | 28544133 | 28548803 | SPTMK |
| PCE_F_Chro3G0241600.1 | PCE_Fruticosa_Chro3 | 28549033 | 28553692 | SPTMK |
| PCE_F_Chro3G0241600.2 | PCE_Fruticosa_Chro3 | 28549033 | 28553692 | SPTMK |
| PCE_F_Chro3G0241600.3 | PCE_Fruticosa_Chro3 | 28549033 | 28553692 | SPTMK |
| PCE_F_Chro3G0241600.4 | PCE_Fruticosa_Chro3 | 28549033 | 28553692 | SPTMK |
| PCE_F_Chro3G0241600.7 | PCE_Fruticosa_Chro3 | 28549033 | 28553692 | SPTMK |
| PCE_F_Chro3G0242400.1 | PCE_Fruticosa_Chro3 | 28675850 | 28678364 | SPTMK |
| PCE_F_Chro3G0242500.1 | PCE_Fruticosa_Chro3 | 28695845 | 28698381 | SPTMK |
| PCE_F_Chro3G0243100.1 | PCE_Fruticosa_Chro3 | 28770340 | 28780921 | SPTMK |
| PCE_F_Chro3G0243100.2 | PCE_Fruticosa_Chro3 | 28770340 | 28780921 | SPTMK |
| PCE_F_Chro3G0243200.1 | PCE_Fruticosa_Chro3 | 28787492 | 28790586 | SPTMK |
| PCE_F_Chro3G0243300.1 | PCE_Fruticosa_Chro3 | 28800265 | 28803105 | SPTMK |
| PCE_F_Chro3G0244100.1 | PCE_Fruticosa_Chro3 | 28870846 | 28873311 | SPTMK |
| PCE_F_Chro3G0244200.1 | PCE_Fruticosa_Chro3 | 28891794 | 28894643 | SPTMK |
| PCE_F_Chro3G0246500.1 | PCE_Fruticosa_Chro3 | 28996595 | 28996967 | LysM |
| PCE_F_Chro3G0246600.1 | PCE_Fruticosa_Chro3 | 28998809 | 28999162 | LysM |
| PCE_F_Chro3G0246600.2 | PCE_Fruticosa_Chro3 | 28998809 | 28999162 | LysM |
| PCE_F_Chro3G0246700.1 | PCE_Fruticosa_Chro3 | 29003758 | 29004185 | LysM |
| PCE_F_Chro3G0246700.2 | PCE_Fruticosa_Chro3 | 29003758 | 29004185 | LysM |
| PCE_F_Chro3G0246800.1 | PCE_Fruticosa_Chro3 | 29004857 | 29005237 | LysM |
| PCE_F_Chro3G0246800.2 | PCE_Fruticosa_Chro3 | 29004857 | 29005237 | LysM |
| PCE_F_Chro3G0246900.1 | PCE_Fruticosa_Chro3 | 29036209 | 29036614 | LysM |
| PCE_F_Chro3G0246900.2 | PCE_Fruticosa_Chro3 | 29036209 | 29036614 | LysM |
| PCE_F_Chro3G0249700.1 | PCE_Fruticosa_Chro3 | 29202770 | 29209958 | RLK |
| PCE_F_Chro3G0249700.2 | PCE_Fruticosa_Chro3 | 29204184 | 29209958 | RLK |
| PCE_F_Chro3G0257800.1 | PCE_Fruticosa_Chro3 | 29782802 | 29786257 | SPTMK |
| PCE_F_Chro3G0257900.1 | PCE_Fruticosa_Chro3 | 29790683 | 29793899 | SPTMK |
| PCE_F_Chro3G0258400.1 | PCE_Fruticosa_Chro3 | 29829246 | 29832132 | SPTMK |
| PCE_F_Chro3G0258700.1 | PCE_Fruticosa_Chro3 | 29838715 | 29842293 | SPTMK |
| PCE_F_Chro3G0258800.1 | PCE_Fruticosa_Chro3 | 29852624 | 29855619 | SPTMK |
| PCE_F_Chro3G0258900.1 | PCE_Fruticosa_Chro3 | 29857306 | 29860678 | SPTMK |
| PCE_F_Chro3G0260800.1 | PCE_Fruticosa_Chro3 | 30021970 | 30027583 | RLK |
| PCE_F_Chro3G0284900.1 | PCE_Fruticosa_Chro3 | 31627230 | 31629869 | RLK |
| PCE_F_Chro3G0290800.1 | PCE_Fruticosa_Chro3 | 31969253 | 31971379 | RLK |
| PCE_F_Chro3G0296800.1 | PCE_Fruticosa_Chro3 | 32318987 | 32321753 | SPTMK |
| PCE_F_Chro3G0311600.1 | PCE_Fruticosa_Chro3 | 33249037 | 33252042 | RLP |
| PCE_F_Chro3G0311600.2 | PCE_Fruticosa_Chro3 | 33249037 | 33252042 | RLP |
| PCE_F_Chro3G0312400.1 | PCE_Fruticosa_Chro3 | 33298341 | 33301228 | LysM |
| PCE_F_Chro3G0313000.1 | PCE_Fruticosa_Chro3 | 33355688 | 33360250 | RLK |
| PCE_F_Chro3G0313000.3 | PCE_Fruticosa_Chro3 | 33355688 | 33360250 | RLK |
| PCE_F_Chro3G0313000.4 | PCE_Fruticosa_Chro3 | 33355688 | 33360250 | RLK |
| PCE_F_Chro3G0325800.1 | PCE_Fruticosa_Chro3 | 34276792 | 34279727 | SPTMK |
| PCE_F_Chro3G0325800.2 | PCE_Fruticosa_Chro3 | 34276792 | 34279727 | SPTMK |
| PCE_F_Chro3G0325800.3 | PCE_Fruticosa_Chro3 | 34276792 | 34279727 | SPTMK |
| PCE_F_Chro3G0325900.1 | PCE_Fruticosa_Chro3 | 34279954 | 34282298 | SPTMK |
| PCE_F_Chro4G0006200.1 | PCE_Fruticosa_Chro4 | 947398 | 955472 | RLK |
| PCE_F_Chro4G0006200.2 | PCE_Fruticosa_Chro4 | 947398 | 955472 | RLK |
| PCE_F_Chro4G0032700.1 | PCE_Fruticosa_Chro4 | 6909801 | 6913039 | SPTMK |
| PCE_F_Chro4G0033600.1 | PCE_Fruticosa_Chro4 | 7096697 | 7099625 | SPTMK |
| PCE_F_Chro4G0041600.1 | PCE_Fruticosa_Chro4 | 8727963 | 8732804 | RLK |
| PCE_F_Chro4G0041600.2 | PCE_Fruticosa_Chro4 | 8727963 | 8732804 | RLK |
| PCE_F_Chro4G0041600.3 | PCE_Fruticosa_Chro4 | 8727963 | 8732804 | RLK |
| PCE_F_Chro4G0045400.1 | PCE_Fruticosa_Chro4 | 9518116 | 9522419 | SPTMK |
| PCE_F_Chro4G0045400.2 | PCE_Fruticosa_Chro4 | 9518116 | 9521575 | SPTMK |
| PCE_F_Chro4G0053300.1 | PCE_Fruticosa_Chro4 | 10668150 | 10681074 | RLP |
| PCE_F_Chro4G0053400.1 | PCE_Fruticosa_Chro4 | 10691070 | 10694327 | RLP |
| PCE_F_Chro4G0054400.1 | PCE_Fruticosa_Chro4 | 10811052 | 10813210 | SPTMK |
| PCE_F_Chro4G0055500.1 | PCE_Fruticosa_Chro4 | 10983875 | 10989874 | SPTMK |
| PCE_F_Chro4G0055500.2 | PCE_Fruticosa_Chro4 | 10983875 | 10989819 | SPTMK |
| PCE_F_Chro4G0055500.3 | PCE_Fruticosa_Chro4 | 10983875 | 10989269 | SPTMK |
| PCE_F_Chro4G0055500.4 | PCE_Fruticosa_Chro4 | 10983875 | 10989819 | SPTMK |
| PCE_F_Chro4G0057600.1 | PCE_Fruticosa_Chro4 | 11390148 | 11392217 | RLK |
| PCE_F_Chro4G0059200.1 | PCE_Fruticosa_Chro4 | 11496911 | 11504489 | RLK |
| PCE_F_Chro4G0059200.2 | PCE_Fruticosa_Chro4 | 11496911 | 11504489 | RLK |
| PCE_F_Chro4G0059200.3 | PCE_Fruticosa_Chro4 | 11496911 | 11504489 | RLK |
| PCE_F_Chro4G0059700.2 | PCE_Fruticosa_Chro4 | 11558921 | 11577299 | RLK |
| PCE_F_Chro4G0059700.3 | PCE_Fruticosa_Chro4 | 11558921 | 11577299 | RLK |
| PCE_F_Chro4G0093300.1 | PCE_Fruticosa_Chro4 | 15425649 | 15428833 | SPTMK |
| PCE_F_Chro4G0093600.1 | PCE_Fruticosa_Chro4 | 15446273 | 15450535 | SPTMK |
| PCE_F_Chro4G0093600.3 | PCE_Fruticosa_Chro4 | 15446273 | 15450535 | SPTMK |
| PCE_F_Chro4G0093600.5 | PCE_Fruticosa_Chro4 | 15446273 | 15449542 | SPTMK |
| PCE_F_Chro4G0093700.1 | PCE_Fruticosa_Chro4 | 15452748 | 15460365 | SPTMK |
| PCE_F_Chro4G0094000.1 | PCE_Fruticosa_Chro4 | 15461120 | 15465640 | SPTMK |
| PCE_F_Chro4G0094200.1 | PCE_Fruticosa_Chro4 | 15469141 | 15474333 | SPTMK |
| PCE_F_Chro4G0095700.1 | PCE_Fruticosa_Chro4 | 15646179 | 15649733 | SPTMK |
| PCE_F_Chro4G0097700.1 | PCE_Fruticosa_Chro4 | 15846537 | 15849731 | SPTMK |
| PCE_F_Chro4G0097800.1 | PCE_Fruticosa_Chro4 | 15880346 | 15884167 | SPTMK |
| PCE_F_Chro4G0098000.1 | PCE_Fruticosa_Chro4 | 15891685 | 15896433 | SPTMK |
| PCE_F_Chro4G0098000.2 | PCE_Fruticosa_Chro4 | 15891685 | 15896433 | SPTMK |
| PCE_F_Chro4G0098900.1 | PCE_Fruticosa_Chro4 | 15974986 | 15984401 | SPTMK |
| PCE_F_Chro4G0099500.1 | PCE_Fruticosa_Chro4 | 16078672 | 16082485 | SPTMK |
| PCE_F_Chro4G0099600.1 | PCE_Fruticosa_Chro4 | 16084632 | 16096302 | SPTMK |
| PCE_F_Chro4G0099600.2 | PCE_Fruticosa_Chro4 | 16084632 | 16096302 | SPTMK |
| PCE_F_Chro4G0099600.5 | PCE_Fruticosa_Chro4 | 16084632 | 16096302 | SPTMK |
| PCE_F_Chro4G0103600.1 | PCE_Fruticosa_Chro4 | 16531362 | 16535333 | SPTMK |
| PCE_F_Chro4G0103600.2 | PCE_Fruticosa_Chro4 | 16531362 | 16535333 | SPTMK |
| PCE_F_Chro4G0116900.1 | PCE_Fruticosa_Chro4 | 17698235 | 17703134 | RLK |
| PCE_F_Chro4G0118900.1 | PCE_Fruticosa_Chro4 | 17870771 | 17872874 | SPTMK |
| PCE_F_Chro4G0119000.1 | PCE_Fruticosa_Chro4 | 17880971 | 17882979 | SPTMK |
| PCE_F_Chro4G0119400.1 | PCE_Fruticosa_Chro4 | 17908454 | 17910559 | SPTMK |
| PCE_F_Chro4G0119500.1 | PCE_Fruticosa_Chro4 | 17929750 | 17940996 | SPTMK |
| PCE_F_Chro4G0119900.1 | PCE_Fruticosa_Chro4 | 18000736 | 18003701 | SPTMK |
| PCE_F_Chro4G0120000.1 | PCE_Fruticosa_Chro4 | 18007521 | 18009951 | SPTMK |
| PCE_F_Chro4G0120300.1 | PCE_Fruticosa_Chro4 | 18065508 | 18067445 | SPTMK |
| PCE_F_Chro4G0120500.1 | PCE_Fruticosa_Chro4 | 18083979 | 18086594 | SPTMK |
| PCE_F_Chro4G0120700.1 | PCE_Fruticosa_Chro4 | 18094334 | 18097452 | SPTMK |
| PCE_F_Chro4G0120700.2 | PCE_Fruticosa_Chro4 | 18094334 | 18097452 | SPTMK |
| PCE_F_Chro4G0120800.1 | PCE_Fruticosa_Chro4 | 18118315 | 18120384 | SPTMK |
| PCE_F_Chro4G0127500.1 | PCE_Fruticosa_Chro4 | 18634175 | 18636424 | RLP |
| PCE_F_Chro4G0127700.1 | PCE_Fruticosa_Chro4 | 18678073 | 18680205 | RLP |
| PCE_F_Chro4G0128500.2 | PCE_Fruticosa_Chro4 | 18776715 | 18779020 | RLP |
| PCE_F_Chro4G0128500.1 | PCE_Fruticosa_Chro4 | 18776715 | 18779020 | RLP |
| PCE_F_Chro4G0128700.1 | PCE_Fruticosa_Chro4 | 18819479 | 18821722 | RLP |
| PCE_F_Chro4G0132600.1 | PCE_Fruticosa_Chro4 | 19099610 | 19105802 | RLP |
| PCE_F_Chro4G0132800.1 | PCE_Fruticosa_Chro4 | 19133858 | 19136101 | RLP |
| PCE_F_Chro4G0132800.2 | PCE_Fruticosa_Chro4 | 19133858 | 19136101 | RLP |
| PCE_F_Chro4G0132900.1 | PCE_Fruticosa_Chro4 | 19167965 | 19170211 | RLP |
| PCE_F_Chro4G0133000.1 | PCE_Fruticosa_Chro4 | 19181109 | 19183313 | RLP |
| PCE_F_Chro4G0133000.2 | PCE_Fruticosa_Chro4 | 19181133 | 19183313 | RLP |
| PCE_F_Chro4G0133100.1 | PCE_Fruticosa_Chro4 | 19186446 | 19188584 | RLP |
| PCE_F_Chro4G0133600.1 | PCE_Fruticosa_Chro4 | 19286430 | 19288720 | RLP |
| PCE_F_Chro4G0133700.1 | PCE_Fruticosa_Chro4 | 19305686 | 19307987 | RLP |
| PCE_F_Chro4G0136600.1 | PCE_Fruticosa_Chro4 | 19500371 | 19502556 | RLP |
| PCE_F_Chro4G0137200.1 | PCE_Fruticosa_Chro4 | 19561111 | 19570168 | RLK |
| PCE_F_Chro4G0137300.1 | PCE_Fruticosa_Chro4 | 19575496 | 19584953 | RLK |
| PCE_F_Chro4G0137300.4 | PCE_Fruticosa_Chro4 | 19575496 | 19584953 | RLK |
| PCE_F_Chro4G0137300.5 | PCE_Fruticosa_Chro4 | 19575496 | 19584953 | RLK |
| PCE_F_Chro4G0137300.8 | PCE_Fruticosa_Chro4 | 19575496 | 19584953 | RLK |
| PCE_F_Chro4G0137300.9 | PCE_Fruticosa_Chro4 | 19575496 | 19584953 | RLK |
| PCE_F_Chro4G0152000.1 | PCE_Fruticosa_Chro4 | 20827081 | 20831404 | RLK |
| PCE_F_Chro4G0152000.2 | PCE_Fruticosa_Chro4 | 20827081 | 20831404 | RLK |
| PCE_F_Chro4G0152000.3 | PCE_Fruticosa_Chro4 | 20827081 | 20832122 | RLK |
| PCE_F_Chro4G0155900.1 | PCE_Fruticosa_Chro4 | 21143579 | 21147056 | SPTMK |
| PCE_F_Chro4G0155900.3 | PCE_Fruticosa_Chro4 | 21143579 | 21146599 | SPTMK |
| PCE_F_Chro4G0155900.4 | PCE_Fruticosa_Chro4 | 21143579 | 21147056 | SPTMK |
| PCE_F_Chro4G0156000.1 | PCE_Fruticosa_Chro4 | 21148580 | 21152129 | SPTMK |
| PCE_F_Chro4G0156000.2 | PCE_Fruticosa_Chro4 | 21148580 | 21152129 | SPTMK |
| PCE_F_Chro4G0156000.4 | PCE_Fruticosa_Chro4 | 21148580 | 21152129 | SPTMK |
| PCE_F_Chro4G0156000.6 | PCE_Fruticosa_Chro4 | 21148580 | 21151697 | SPTMK |
| PCE_F_Chro4G0156000.7 | PCE_Fruticosa_Chro4 | 21148580 | 21152129 | SPTMK |
| PCE_F_Chro4G0156300.1 | PCE_Fruticosa_Chro4 | 21169713 | 21173506 | SPTMK |
| PCE_F_Chro4G0156300.2 | PCE_Fruticosa_Chro4 | 21169713 | 21173506 | SPTMK |
| PCE_F_Chro4G0161300.2 | PCE_Fruticosa_Chro4 | 21523880 | 21527460 | SPTMK |
| PCE_F_Chro4G0161400.1 | PCE_Fruticosa_Chro4 | 21531516 | 21535101 | SPTMK |
| PCE_F_Chro4G0161400.2 | PCE_Fruticosa_Chro4 | 21531516 | 21535101 | SPTMK |
| PCE_F_Chro4G0161400.4 | PCE_Fruticosa_Chro4 | 21531516 | 21535101 | SPTMK |
| PCE_F_Chro4G0163400.1 | PCE_Fruticosa_Chro4 | 21711765 | 21721263 | RLK |
| PCE_F_Chro4G0163400.2 | PCE_Fruticosa_Chro4 | 21711765 | 21721263 | RLK |
| PCE_F_Chro4G0163400.3 | PCE_Fruticosa_Chro4 | 21711765 | 21721263 | RLK |
| PCE_F_Chro4G0165400.1 | PCE_Fruticosa_Chro4 | 21922625 | 21925665 | SPTMK |
| PCE_F_Chro4G0165500.1 | PCE_Fruticosa_Chro4 | 21927164 | 21930269 | SPTMK |
| PCE_F_Chro4G0166000.1 | PCE_Fruticosa_Chro4 | 21975815 | 21981124 | SPTMK |
| PCE_F_Chro4G0166000.2 | PCE_Fruticosa_Chro4 | 21975815 | 21981124 | SPTMK |
| PCE_F_Chro4G0166200.1 | PCE_Fruticosa_Chro4 | 22011394 | 22015000 | SPTMK |
| PCE_F_Chro4G0166300.1 | PCE_Fruticosa_Chro4 | 22023264 | 22026875 | SPTMK |
| PCE_F_Chro4G0166400.1 | PCE_Fruticosa_Chro4 | 22027992 | 22034067 | SPTMK |
| PCE_F_Chro4G0166400.2 | PCE_Fruticosa_Chro4 | 22027992 | 22034067 | SPTMK |
| PCE_F_Chro4G0166400.4 | PCE_Fruticosa_Chro4 | 22027992 | 22034067 | SPTMK |
| PCE_F_Chro4G0166400.5 | PCE_Fruticosa_Chro4 | 22027992 | 22034067 | SPTMK |
| PCE_F_Chro4G0166400.3 | PCE_Fruticosa_Chro4 | 22028807 | 22034067 | SPTMK |
| PCE_F_Chro4G0169600.1 | PCE_Fruticosa_Chro4 | 22249066 | 22252021 | RLP |
| PCE_F_Chro4G0169700.1 | PCE_Fruticosa_Chro4 | 22283543 | 22286729 | RLP |
| PCE_F_Chro4G0169900.1 | PCE_Fruticosa_Chro4 | 22321534 | 22324431 | RLP |
| PCE_F_Chro4G0172500.3 | PCE_Fruticosa_Chro4 | 22472163 | 22475802 | RLK |
| PCE_F_Chro4G0172500.1 | PCE_Fruticosa_Chro4 | 22472163 | 22475802 | RLK |
| PCE_F_Chro4G0172900.1 | PCE_Fruticosa_Chro4 | 22518349 | 22527767 | RLK |
| PCE_F_Chro4G0173200.1 | PCE_Fruticosa_Chro4 | 22545724 | 22553895 | RLK |
| PCE_F_Chro4G0173200.2 | PCE_Fruticosa_Chro4 | 22545724 | 22553483 | RLK |
| PCE_F_Chro4G0173800.1 | PCE_Fruticosa_Chro4 | 22681213 | 22684583 | RLK |
| PCE_F_Chro4G0179600.3 | PCE_Fruticosa_Chro4 | 23206001 | 23209614 | RLP |
| PCE_F_Chro4G0180900.1 | PCE_Fruticosa_Chro4 | 23333696 | 23338351 | RLK |
| PCE_F_Chro4G0180900.2 | PCE_Fruticosa_Chro4 | 23333696 | 23338351 | RLK |
| PCE_F_Chro4G0182300.1 | PCE_Fruticosa_Chro4 | 23416095 | 23419999 | RLK |
| PCE_F_Chro4G0184800.1 | PCE_Fruticosa_Chro4 | 23604485 | 23608463 | SPTMK |
| PCE_F_Chro4G0184800.2 | PCE_Fruticosa_Chro4 | 23604485 | 23608496 | SPTMK |
| PCE_F_Chro4G0184900.1 | PCE_Fruticosa_Chro4 | 23609984 | 23613476 | SPTMK |
| PCE_F_Chro4G0184900.2 | PCE_Fruticosa_Chro4 | 23609984 | 23613476 | SPTMK |
| PCE_F_Chro4G0184900.3 | PCE_Fruticosa_Chro4 | 23609984 | 23613524 | SPTMK |
| PCE_F_Chro4G0184900.4 | PCE_Fruticosa_Chro4 | 23609984 | 23613476 | SPTMK |
| PCE_F_Chro4G0184900.6 | PCE_Fruticosa_Chro4 | 23610439 | 23613476 | SPTMK |
| PCE_F_Chro4G0185100.1 | PCE_Fruticosa_Chro4 | 23641457 | 23645526 | SPTMK |
| PCE_F_Chro4G0185100.3 | PCE_Fruticosa_Chro4 | 23641457 | 23645526 | SPTMK |
| PCE_F_Chro4G0185600.1 | PCE_Fruticosa_Chro4 | 23713260 | 23717269 | RLK |
| PCE_F_Chro4G0185600.2 | PCE_Fruticosa_Chro4 | 23713260 | 23717269 | RLK |
| PCE_F_Chro4G0186800.1 | PCE_Fruticosa_Chro4 | 23850256 | 23853722 | RLP |
| PCE_F_Chro4G0187000.1 | PCE_Fruticosa_Chro4 | 23882753 | 23885990 | RLP |
| PCE_F_Chro4G0199100.1 | PCE_Fruticosa_Chro4 | 24934753 | 24938129 | RLK |
| PCE_F_Chro4G0200200.1 | PCE_Fruticosa_Chro4 | 25003162 | 25011549 | RLK |
| PCE_F_Chro4G0200200.2 | PCE_Fruticosa_Chro4 | 25003162 | 25011549 | RLK |
| PCE_F_Chro4G0200200.3 | PCE_Fruticosa_Chro4 | 25003162 | 25011549 | RLK |
| PCE_F_Chro4G0200200.4 | PCE_Fruticosa_Chro4 | 25003162 | 25011549 | RLK |
| PCE_F_Chro4G0200200.5 | PCE_Fruticosa_Chro4 | 25003162 | 25011549 | RLK |
| PCE_F_Chro4G0200200.6 | PCE_Fruticosa_Chro4 | 25003162 | 25011549 | RLK |
| PCE_F_Chro4G0200200.7 | PCE_Fruticosa_Chro4 | 25003162 | 25011549 | RLK |
| PCE_F_Chro4G0200200.8 | PCE_Fruticosa_Chro4 | 25003162 | 25011549 | RLK |
| PCE_F_Chro4G0200400.1 | PCE_Fruticosa_Chro4 | 25027834 | 25034370 | RLK |
| PCE_F_Chro4G0200400.2 | PCE_Fruticosa_Chro4 | 25027834 | 25034352 | RLK |
| PCE_F_Chro4G0200400.3 | PCE_Fruticosa_Chro4 | 25027834 | 25034370 | RLK |
| PCE_F_Chro4G0200400.4 | PCE_Fruticosa_Chro4 | 25027834 | 25034361 | RLK |
| PCE_F_Chro4G0200400.5 | PCE_Fruticosa_Chro4 | 25027834 | 25034617 | RLK |
| PCE_F_Chro4G0200400.6 | PCE_Fruticosa_Chro4 | 25027834 | 25034361 | RLK |
| PCE_F_Chro4G0200800.1 | PCE_Fruticosa_Chro4 | 25049956 | 25063684 | RLK |
| PCE_F_Chro4G0200800.2 | PCE_Fruticosa_Chro4 | 25049956 | 25063684 | RLK |
| PCE_F_Chro4G0200800.3 | PCE_Fruticosa_Chro4 | 25049956 | 25063684 | RLK |
| PCE_F_Chro4G0200800.4 | PCE_Fruticosa_Chro4 | 25049956 | 25063684 | RLK |
| PCE_F_Chro4G0200800.5 | PCE_Fruticosa_Chro4 | 25049956 | 25063684 | RLK |
| PCE_F_Chro4G0200900.1 | PCE_Fruticosa_Chro4 | 25068015 | 25074619 | RLK |
| PCE_F_Chro4G0200900.2 | PCE_Fruticosa_Chro4 | 25068015 | 25074619 | RLK |
| PCE_F_Chro4G0200900.3 | PCE_Fruticosa_Chro4 | 25068015 | 25074619 | RLK |
| PCE_F_Chro4G0200900.4 | PCE_Fruticosa_Chro4 | 25068015 | 25074619 | RLK |
| PCE_F_Chro4G0200900.5 | PCE_Fruticosa_Chro4 | 25068015 | 25074619 | RLK |
| PCE_F_Chro4G0205200.1 | PCE_Fruticosa_Chro4 | 25338135 | 25340682 | SPTMK |
| PCE_F_Chro4G0205200.3 | PCE_Fruticosa_Chro4 | 25338135 | 25340682 | SPTMK |
| PCE_F_Chro4G0205400.1 | PCE_Fruticosa_Chro4 | 25348087 | 25350790 | SPTMK |
| PCE_F_Chro4G0205400.3 | PCE_Fruticosa_Chro4 | 25348087 | 25350790 | SPTMK |
| PCE_F_Chro4G0205600.1 | PCE_Fruticosa_Chro4 | 25358688 | 25360382 | RLK |
| PCE_F_Chro4G0205600.2 | PCE_Fruticosa_Chro4 | 25358688 | 25360382 | RLK |
| PCE_F_Chro4G0211500.1 | PCE_Fruticosa_Chro4 | 25803574 | 25808080 | SPTMK |
| PCE_F_Chro4G0211600.1 | PCE_Fruticosa_Chro4 | 25808721 | 25811706 | SPTMK |
| PCE_F_Chro4G0211600.2 | PCE_Fruticosa_Chro4 | 25808754 | 25811706 | SPTMK |
| PCE_F_Chro4G0211700.2 | PCE_Fruticosa_Chro4 | 25837580 | 25840503 | SPTMK |
| PCE_F_Chro4G0212000.1 | PCE_Fruticosa_Chro4 | 25904800 | 25913754 | SPTMK |
| PCE_F_Chro4G0212400.1 | PCE_Fruticosa_Chro4 | 25943359 | 25946329 | SPTMK |
| PCE_F_Chro4G0212400.2 | PCE_Fruticosa_Chro4 | 25943389 | 25946329 | SPTMK |
| PCE_F_Chro4G0212500.1 | PCE_Fruticosa_Chro4 | 25957653 | 25960414 | SPTMK |
| PCE_F_Chro4G0212500.2 | PCE_Fruticosa_Chro4 | 25957671 | 25960414 | SPTMK |
| PCE_F_Chro4G0212700.1 | PCE_Fruticosa_Chro4 | 26015118 | 26018421 | SPTMK |
| PCE_F_Chro4G0212800.1 | PCE_Fruticosa_Chro4 | 26054418 | 26057116 | SPTMK |
| PCE_F_Chro4G0213300.1 | PCE_Fruticosa_Chro4 | 26102961 | 26105410 | SPTMK |
| PCE_F_Chro4G0213700.1 | PCE_Fruticosa_Chro4 | 26129792 | 26136764 | SPTMK |
| PCE_F_Chro4G0213700.2 | PCE_Fruticosa_Chro4 | 26129792 | 26136764 | SPTMK |
| PCE_F_Chro4G0213700.3 | PCE_Fruticosa_Chro4 | 26129792 | 26136764 | SPTMK |
| PCE_F_Chro4G0213700.4 | PCE_Fruticosa_Chro4 | 26129792 | 26136764 | SPTMK |
| PCE_F_Chro4G0213700.5 | PCE_Fruticosa_Chro4 | 26129792 | 26136764 | SPTMK |
| PCE_F_Chro4G0213700.6 | PCE_Fruticosa_Chro4 | 26129792 | 26136764 | SPTMK |
| PCE_F_Chro4G0214100.1 | PCE_Fruticosa_Chro4 | 26181205 | 26184318 | SPTMK |
| PCE_F_Chro4G0214100.2 | PCE_Fruticosa_Chro4 | 26181205 | 26184318 | SPTMK |
| PCE_F_Chro4G0214300.1 | PCE_Fruticosa_Chro4 | 26196361 | 26198815 | SPTMK |
| PCE_F_Chro4G0215000.1 | PCE_Fruticosa_Chro4 | 26233190 | 26238407 | RLK |
| PCE_F_Chro4G0215300.1 | PCE_Fruticosa_Chro4 | 26251745 | 26255062 | SPTMK |
| PCE_F_Chro4G0225000.1 | PCE_Fruticosa_Chro4 | 27006154 | 27011397 | RLK |
| PCE_F_Chro4G0225000.2 | PCE_Fruticosa_Chro4 | 27006154 | 27012735 | RLK |
| PCE_F_Chro4G0225000.3 | PCE_Fruticosa_Chro4 | 27006154 | 27012735 | RLK |
| PCE_F_Chro4G0225000.5 | PCE_Fruticosa_Chro4 | 27006154 | 27012735 | RLK |
| PCE_F_Chro4G0225100.1 | PCE_Fruticosa_Chro4 | 27027251 | 27032210 | RLK |
| PCE_F_Chro4G0230700.1 | PCE_Fruticosa_Chro4 | 27425641 | 27429688 | RLK |
| PCE_F_Chro4G0236700.1 | PCE_Fruticosa_Chro4 | 27792581 | 27799245 | RLK |
| PCE_F_Chro4G0236700.2 | PCE_Fruticosa_Chro4 | 27792581 | 27799245 | RLK |
| PCE_F_Chro4G0236800.2 | PCE_Fruticosa_Chro4 | 27800929 | 27808640 | RLK |
| PCE_F_Chro4G0236800.1 | PCE_Fruticosa_Chro4 | 27800929 | 27808640 | RLK |
| PCE_F_Chro4G0236900.1 | PCE_Fruticosa_Chro4 | 27810804 | 27817627 | RLK |
| PCE_F_Chro4G0237600.1 | PCE_Fruticosa_Chro4 | 27843437 | 27860469 | RLK |
| PCE_F_Chro4G0237600.2 | PCE_Fruticosa_Chro4 | 27843437 | 27858341 | RLK |
| PCE_F_Chro4G0237700.1 | PCE_Fruticosa_Chro4 | 27873044 | 27882622 | RLK |
| PCE_F_Chro4G0237700.2 | PCE_Fruticosa_Chro4 | 27873044 | 27882622 | RLK |
| PCE_F_Chro4G0238000.1 | PCE_Fruticosa_Chro4 | 27939152 | 27954890 | RLK |
| PCE_F_Chro4G0238000.2 | PCE_Fruticosa_Chro4 | 27939152 | 27947079 | RLK |
| PCE_F_Chro4G0238000.3 | PCE_Fruticosa_Chro4 | 27939152 | 27947043 | RLK |
| PCE_F_Chro4G0238300.1 | PCE_Fruticosa_Chro4 | 27972717 | 27980422 | RLK |
| PCE_F_Chro4G0238300.2 | PCE_Fruticosa_Chro4 | 27972717 | 27982315 | RLK |
| PCE_F_Chro4G0238300.3 | PCE_Fruticosa_Chro4 | 27972717 | 27980422 | RLK |
| PCE_F_Chro4G0238300.4 | PCE_Fruticosa_Chro4 | 27972717 | 27982315 | RLK |
| PCE_F_Chro4G0238300.5 | PCE_Fruticosa_Chro4 | 27972717 | 27980422 | RLK |
| PCE_F_Chro4G0238300.6 | PCE_Fruticosa_Chro4 | 27972717 | 27982315 | RLK |
| PCE_F_Chro4G0238700.1 | PCE_Fruticosa_Chro4 | 28040135 | 28046867 | RLK |
| PCE_F_Chro4G0238700.2 | PCE_Fruticosa_Chro4 | 28040135 | 28046867 | RLK |
| PCE_F_Chro4G0238700.3 | PCE_Fruticosa_Chro4 | 28040135 | 28046831 | RLK |
| PCE_F_Chro4G0238700.4 | PCE_Fruticosa_Chro4 | 28040135 | 28046867 | RLK |
| PCE_F_Chro4G0238800.1 | PCE_Fruticosa_Chro4 | 28049028 | 28057276 | RLK |
| PCE_F_Chro4G0238900.1 | PCE_Fruticosa_Chro4 | 28066470 | 28074137 | RLK |
| PCE_F_Chro4G0238900.2 | PCE_Fruticosa_Chro4 | 28066470 | 28074137 | RLK |
| PCE_F_Chro4G0242500.1 | PCE_Fruticosa_Chro4 | 28309233 | 28310714 | RLP |
| PCE_F_Chro4G0243200.3 | PCE_Fruticosa_Chro4 | 28327817 | 28332497 | RLK |
| PCE_F_Chro4G0243200.1 | PCE_Fruticosa_Chro4 | 28327817 | 28332497 | RLK |
| PCE_F_Chro4G0243200.2 | PCE_Fruticosa_Chro4 | 28327817 | 28332497 | RLK |
| PCE_F_Chro4G0243500.2 | PCE_Fruticosa_Chro4 | 28343530 | 28345913 | RLK |
| PCE_F_Chro4G0243700.5 | PCE_Fruticosa_Chro4 | 28383386 | 28387968 | RLK |
| PCE_F_Chro4G0243700.1 | PCE_Fruticosa_Chro4 | 28383386 | 28387968 | RLK |
| PCE_F_Chro4G0243700.3 | PCE_Fruticosa_Chro4 | 28383386 | 28387968 | RLK |
| PCE_F_Chro4G0243700.2 | PCE_Fruticosa_Chro4 | 28383413 | 28387968 | RLK |
| PCE_F_Chro4G0244200.1 | PCE_Fruticosa_Chro4 | 28455512 | 28457942 | RLK |
| PCE_F_Chro4G0244800.1 | PCE_Fruticosa_Chro4 | 28472222 | 28477674 | RLK |
| PCE_F_Chro4G0244800.2 | PCE_Fruticosa_Chro4 | 28472222 | 28477674 | RLK |
| PCE_F_Chro4G0247100.1 | PCE_Fruticosa_Chro4 | 28593861 | 28598633 | RLK |
| PCE_F_Chro4G0253600.1 | PCE_Fruticosa_Chro4 | 28998261 | 29002226 | RLK |
| PCE_F_Chro4G0255300.1 | PCE_Fruticosa_Chro4 | 29099306 | 29104926 | RLP |
| PCE_F_Chro4G0255300.2 | PCE_Fruticosa_Chro4 | 29099306 | 29104926 | RLP |
| PCE_F_Chro4G0255300.4 | PCE_Fruticosa_Chro4 | 29099945 | 29104926 | RLP |
| PCE_F_Chro4G0266000.1 | PCE_Fruticosa_Chro4 | 29779344 | 29784489 | RLK |
| PCE_F_Chro4G0266000.2 | PCE_Fruticosa_Chro4 | 29779371 | 29784489 | RLK |
| PCE_F_Chro4G0267900.1 | PCE_Fruticosa_Chro4 | 29921843 | 29922243 | LysM |
| PCE_F_Chro4G0273000.2 | PCE_Fruticosa_Chro4 | 30274945 | 30278381 | SPTMK |
| PCE_F_Chro4G0273000.3 | PCE_Fruticosa_Chro4 | 30274972 | 30278381 | SPTMK |
| PCE_F_Chro4G0273000.1 | PCE_Fruticosa_Chro4 | 30274975 | 30278381 | SPTMK |
| PCE_F_Chro4G0273100.1 | PCE_Fruticosa_Chro4 | 30279329 | 30283224 | SPTMK |
| PCE_F_Chro4G0273100.2 | PCE_Fruticosa_Chro4 | 30279377 | 30283224 | SPTMK |
| PCE_F_Chro4G0273200.1 | PCE_Fruticosa_Chro4 | 30283836 | 30287436 | SPTMK |
| PCE_F_Chro4G0273200.2 | PCE_Fruticosa_Chro4 | 30283836 | 30287436 | SPTMK |
| PCE_F_Chro4G0273200.3 | PCE_Fruticosa_Chro4 | 30283836 | 30287436 | SPTMK |
| PCE_F_Chro4G0273300.1 | PCE_Fruticosa_Chro4 | 30298648 | 30302073 | SPTMK |
| PCE_F_Chro4G0273300.3 | PCE_Fruticosa_Chro4 | 30298648 | 30302073 | SPTMK |
| PCE_F_Chro4G0273400.1 | PCE_Fruticosa_Chro4 | 30303026 | 30309061 | SPTMK |
| PCE_F_Chro4G0273400.2 | PCE_Fruticosa_Chro4 | 30303026 | 30309061 | SPTMK |
| PCE_F_Chro4G0273500.1 | PCE_Fruticosa_Chro4 | 30310439 | 30321798 | SPTMK |
| PCE_F_Chro4G0273500.2 | PCE_Fruticosa_Chro4 | 30310439 | 30321798 | SPTMK |
| PCE_F_Chro4G0273500.3 | PCE_Fruticosa_Chro4 | 30310439 | 30321798 | SPTMK |
| PCE_F_Chro4G0273600.1 | PCE_Fruticosa_Chro4 | 30326751 | 30329976 | SPTMK |
| PCE_F_Chro4G0273600.2 | PCE_Fruticosa_Chro4 | 30326751 | 30329295 | SPTMK |
| PCE_F_Chro4G0273700.1 | PCE_Fruticosa_Chro4 | 30331430 | 30334928 | SPTMK |
| PCE_F_Chro4G0273700.3 | PCE_Fruticosa_Chro4 | 30332246 | 30334928 | SPTMK |
| PCE_F_Chro4G0273800.1 | PCE_Fruticosa_Chro4 | 30337815 | 30341139 | SPTMK |
| PCE_F_Chro4G0273800.5 | PCE_Fruticosa_Chro4 | 30337815 | 30341139 | SPTMK |
| PCE_F_Chro4G0273800.3 | PCE_Fruticosa_Chro4 | 30338556 | 30341139 | SPTMK |
| PCE_F_Chro4G0273900.1 | PCE_Fruticosa_Chro4 | 30341673 | 30345124 | SPTMK |
| PCE_F_Chro4G0273900.3 | PCE_Fruticosa_Chro4 | 30341673 | 30345124 | SPTMK |
| PCE_F_Chro4G0274100.1 | PCE_Fruticosa_Chro4 | 30349292 | 30352591 | SPTMK |
| PCE_F_Chro4G0274100.3 | PCE_Fruticosa_Chro4 | 30350040 | 30352591 | SPTMK |
| PCE_F_Chro4G0274200.1 | PCE_Fruticosa_Chro4 | 30353907 | 30361105 | SPTMK |
| PCE_F_Chro4G0274200.3 | PCE_Fruticosa_Chro4 | 30354508 | 30361105 | SPTMK |
| PCE_F_Chro4G0274300.1 | PCE_Fruticosa_Chro4 | 30363422 | 30366871 | SPTMK |
| PCE_F_Chro4G0274400.1 | PCE_Fruticosa_Chro4 | 30367988 | 30371317 | SPTMK |
| PCE_F_Chro4G0274500.1 | PCE_Fruticosa_Chro4 | 30372602 | 30376544 | SPTMK |
| PCE_F_Chro4G0274500.3 | PCE_Fruticosa_Chro4 | 30372602 | 30376544 | SPTMK |
| PCE_F_Chro4G0274600.1 | PCE_Fruticosa_Chro4 | 30378372 | 30381740 | SPTMK |
| PCE_F_Chro4G0274800.1 | PCE_Fruticosa_Chro4 | 30388427 | 30394885 | SPTMK |
| PCE_F_Chro4G0274900.1 | PCE_Fruticosa_Chro4 | 30395617 | 30399267 | SPTMK |
| PCE_F_Chro4G0274900.2 | PCE_Fruticosa_Chro4 | 30395617 | 30399267 | SPTMK |
| PCE_F_Chro4G0274900.3 | PCE_Fruticosa_Chro4 | 30395617 | 30398397 | SPTMK |
| PCE_F_Chro4G0274900.6 | PCE_Fruticosa_Chro4 | 30395617 | 30399267 | SPTMK |
| PCE_F_Chro4G0277800.1 | PCE_Fruticosa_Chro4 | 30538899 | 30541579 | SPTMK |
| PCE_F_Chro4G0277800.2 | PCE_Fruticosa_Chro4 | 30538899 | 30541579 | SPTMK |
| PCE_F_Chro4G0277800.3 | PCE_Fruticosa_Chro4 | 30538899 | 30541579 | SPTMK |
| PCE_F_Chro4G0277800.5 | PCE_Fruticosa_Chro4 | 30538899 | 30541579 | SPTMK |
| PCE_F_Chro4G0277800.7 | PCE_Fruticosa_Chro4 | 30538899 | 30541579 | SPTMK |
| PCE_F_Chro4G0277800.8 | PCE_Fruticosa_Chro4 | 30539642 | 30541579 | SPTMK |
| PCE_F_Chro4G0277900.1 | PCE_Fruticosa_Chro4 | 30542236 | 30555240 | SPTMK |
| PCE_F_Chro4G0278400.1 | PCE_Fruticosa_Chro4 | 30569333 | 30572246 | SPTMK |
| PCE_F_Chro4G0278600.1 | PCE_Fruticosa_Chro4 | 30585547 | 30588094 | SPTMK |
| PCE_F_Chro4G0279100.1 | PCE_Fruticosa_Chro4 | 30631653 | 30637395 | SPTMK |
| PCE_F_Chro4G0279100.3 | PCE_Fruticosa_Chro4 | 30631653 | 30637395 | SPTMK |
| PCE_F_Chro4G0279400.1 | PCE_Fruticosa_Chro4 | 30660481 | 30663382 | SPTMK |
| PCE_F_Chro4G0279400.3 | PCE_Fruticosa_Chro4 | 30661323 | 30663382 | SPTMK |
| PCE_F_Chro4G0279500.1 | PCE_Fruticosa_Chro4 | 30665834 | 30681010 | SPTMK |
| PCE_F_Chro4G0279500.3 | PCE_Fruticosa_Chro4 | 30665834 | 30668381 | SPTMK |
| PCE_F_Chro4G0279500.4 | PCE_Fruticosa_Chro4 | 30665834 | 30681010 | SPTMK |
| PCE_F_Chro4G0279500.5 | PCE_Fruticosa_Chro4 | 30665834 | 30681010 | SPTMK |
| PCE_F_Chro4G0279800.1 | PCE_Fruticosa_Chro4 | 30685517 | 30688598 | SPTMK |
| PCE_F_Chro4G0279800.3 | PCE_Fruticosa_Chro4 | 30685517 | 30688598 | SPTMK |
| PCE_F_Chro4G0279800.2 | PCE_Fruticosa_Chro4 | 30686401 | 30688598 | SPTMK |
| PCE_F_Chro4G0279900.1 | PCE_Fruticosa_Chro4 | 30690469 | 30693559 | SPTMK |
| PCE_F_Chro4G0279900.3 | PCE_Fruticosa_Chro4 | 30690469 | 30693559 | SPTMK |
| PCE_F_Chro4G0279900.2 | PCE_Fruticosa_Chro4 | 30691364 | 30693559 | SPTMK |
| PCE_F_Chro4G0280000.2 | PCE_Fruticosa_Chro4 | 30695343 | 30698674 | SPTMK |
| PCE_F_Chro4G0280000.3 | PCE_Fruticosa_Chro4 | 30695343 | 30698674 | SPTMK |
| PCE_F_Chro4G0280000.4 | PCE_Fruticosa_Chro4 | 30695343 | 30698674 | SPTMK |
| PCE_F_Chro4G0280200.1 | PCE_Fruticosa_Chro4 | 30732903 | 30735865 | SPTMK |
| PCE_F_Chro4G0280200.2 | PCE_Fruticosa_Chro4 | 30732903 | 30735865 | SPTMK |
| PCE_F_Chro4G0280200.3 | PCE_Fruticosa_Chro4 | 30732903 | 30735872 | SPTMK |
| PCE_F_Chro4G0280200.4 | PCE_Fruticosa_Chro4 | 30732903 | 30735865 | SPTMK |
| PCE_F_Chro4G0280200.5 | PCE_Fruticosa_Chro4 | 30732903 | 30735872 | SPTMK |
| PCE_F_Chro4G0280200.6 | PCE_Fruticosa_Chro4 | 30732903 | 30735865 | SPTMK |
| PCE_F_Chro4G0280500.1 | PCE_Fruticosa_Chro4 | 30751302 | 30754239 | SPTMK |
| PCE_F_Chro4G0280500.3 | PCE_Fruticosa_Chro4 | 30751302 | 30754239 | SPTMK |
| PCE_F_Chro4G0280500.4 | PCE_Fruticosa_Chro4 | 30751302 | 30754227 | SPTMK |
| PCE_F_Chro4G0280700.1 | PCE_Fruticosa_Chro4 | 30785696 | 30788508 | SPTMK |
| PCE_F_Chro4G0280700.2 | PCE_Fruticosa_Chro4 | 30786328 | 30788508 | SPTMK |
| PCE_F_Chro4G0281300.1 | PCE_Fruticosa_Chro4 | 30830986 | 30833725 | SPTMK |
| PCE_F_Chro4G0281300.2 | PCE_Fruticosa_Chro4 | 30830986 | 30833719 | SPTMK |
| PCE_F_Chro4G0281300.3 | PCE_Fruticosa_Chro4 | 30830986 | 30833719 | SPTMK |
| PCE_F_Chro4G0281400.1 | PCE_Fruticosa_Chro4 | 30834820 | 30837389 | SPTMK |
| PCE_F_Chro4G0281400.2 | PCE_Fruticosa_Chro4 | 30834820 | 30837389 | SPTMK |
| PCE_F_Chro4G0292900.1 | PCE_Fruticosa_Chro4 | 31408817 | 31419343 | SPTMK |
| PCE_F_Chro4G0292900.2 | PCE_Fruticosa_Chro4 | 31408817 | 31419343 | SPTMK |
| PCE_F_Chro4G0292900.3 | PCE_Fruticosa_Chro4 | 31408817 | 31419367 | SPTMK |
| PCE_F_Chro4G0292900.4 | PCE_Fruticosa_Chro4 | 31408817 | 31419343 | SPTMK |
| PCE_F_Chro4G0293200.1 | PCE_Fruticosa_Chro4 | 31433034 | 31438806 | RLK |
| PCE_F_Chro4G0293200.2 | PCE_Fruticosa_Chro4 | 31433034 | 31438806 | RLK |
| PCE_F_Chro4G0296400.2 | PCE_Fruticosa_Chro4 | 31617184 | 31618762 | SPTMK |
| PCE_F_Chro4G0299900.1 | PCE_Fruticosa_Chro4 | 31739186 | 31743064 | CC-NB-LRR |
| PCE_F_Chro4G0299900.2 | PCE_Fruticosa_Chro4 | 31739186 | 31743064 | CC-NB-LRR |
| PCE_F_Chro5G0002000.1 | PCE_Fruticosa_Chro5 | 553620 | 557254 | CC-NB-LRR |
| PCE_F_Chro5G0002000.2 | PCE_Fruticosa_Chro5 | 553620 | 557254 | CC-NB-LRR |
| PCE_F_Chro5G0002000.3 | PCE_Fruticosa_Chro5 | 553620 | 557254 | CC-NB-LRR |
| PCE_F_Chro5G0002100.1 | PCE_Fruticosa_Chro5 | 600488 | 603301 | CC-NB-LRR |
| PCE_F_Chro5G0002100.2 | PCE_Fruticosa_Chro5 | 600488 | 603301 | CC-NB-LRR |
| PCE_F_Chro5G0002100.3 | PCE_Fruticosa_Chro5 | 600488 | 603301 | CC-NB-LRR |
| PCE_F_Chro5G0002200.1 | PCE_Fruticosa_Chro5 | 679645 | 683914 | CC-NB-LRR |
| PCE_F_Chro5G0002200.2 | PCE_Fruticosa_Chro5 | 679645 | 683914 | CC-NB-LRR |
| PCE_F_Chro5G0002200.3 | PCE_Fruticosa_Chro5 | 679645 | 683914 | CC-NB-LRR |
| PCE_F_Chro5G0002300.1 | PCE_Fruticosa_Chro5 | 688411 | 691260 | CC-NB-LRR |
| PCE_F_Chro5G0002300.2 | PCE_Fruticosa_Chro5 | 688411 | 691260 | CC-NB-LRR |
| PCE_F_Chro5G0002300.3 | PCE_Fruticosa_Chro5 | 688411 | 691260 | CC-NB-LRR |
| PCE_F_Chro5G0002600.1 | PCE_Fruticosa_Chro5 | 718789 | 722016 | CC-NB-LRR |
| PCE_F_Chro5G0002700.1 | PCE_Fruticosa_Chro5 | 742767 | 746005 | CC-NB-LRR |
| PCE_F_Chro5G0003000.1 | PCE_Fruticosa_Chro5 | 765992 | 769196 | CC-NB-LRR |
| PCE_F_Chro5G0005500.1 | PCE_Fruticosa_Chro5 | 1203425 | 1220549 | RLK |
| PCE_F_Chro5G0005500.2 | PCE_Fruticosa_Chro5 | 1203425 | 1232328 | RLK |
| PCE_F_Chro5G0005500.3 | PCE_Fruticosa_Chro5 | 1203425 | 1220549 | RLK |
| PCE_F_Chro5G0005500.4 | PCE_Fruticosa_Chro5 | 1203425 | 1232133 | RLK |
| PCE_F_Chro5G0005500.5 | PCE_Fruticosa_Chro5 | 1203425 | 1232133 | RLK |
| PCE_F_Chro5G0005500.6 | PCE_Fruticosa_Chro5 | 1203425 | 1220549 | RLK |
| PCE_F_Chro5G0008700.1 | PCE_Fruticosa_Chro5 | 1852587 | 1863382 | RPW8-NB-LRR |
| PCE_F_Chro5G0011100.1 | PCE_Fruticosa_Chro5 | 2309083 | 2309757 | RLP |
| PCE_F_Chro5G0017600.4 | PCE_Fruticosa_Chro5 | 3498035 | 3507684 | RLK |
| PCE_F_Chro5G0028000.1 | PCE_Fruticosa_Chro5 | 5530264 | 5533521 | SPTMK |
| PCE_F_Chro5G0032900.1 | PCE_Fruticosa_Chro5 | 6330665 | 6334555 | TIR-NB-LRR |
| PCE_F_Chro5G0032900.2 | PCE_Fruticosa_Chro5 | 6330665 | 6334555 | TIR-NB-LRR |
| PCE_F_Chro5G0044600.1 | PCE_Fruticosa_Chro5 | 8395603 | 8399743 | TIR-NB-LRR |
| PCE_F_Chro5G0047900.1 | PCE_Fruticosa_Chro5 | 8706344 | 8708605 | RLP |
| PCE_F_Chro5G0048600.1 | PCE_Fruticosa_Chro5 | 8797203 | 8801281 | TIR-NB-LRR |
| PCE_F_Chro5G0054600.1 | PCE_Fruticosa_Chro5 | 9613998 | 9617598 | SPTMK |
| PCE_F_Chro5G0054600.2 | PCE_Fruticosa_Chro5 | 9613998 | 9617598 | SPTMK |
| PCE_F_Chro5G0054600.3 | PCE_Fruticosa_Chro5 | 9613998 | 9617598 | SPTMK |
| PCE_F_Chro5G0054600.4 | PCE_Fruticosa_Chro5 | 9613998 | 9617598 | SPTMK |
| PCE_F_Chro5G0054600.5 | PCE_Fruticosa_Chro5 | 9613998 | 9617598 | SPTMK |
| PCE_F_Chro5G0054700.1 | PCE_Fruticosa_Chro5 | 9619900 | 9623348 | SPTMK |
| PCE_F_Chro5G0054700.2 | PCE_Fruticosa_Chro5 | 9619900 | 9623348 | SPTMK |
| PCE_F_Chro5G0054700.3 | PCE_Fruticosa_Chro5 | 9619900 | 9623348 | SPTMK |
| PCE_F_Chro5G0054900.1 | PCE_Fruticosa_Chro5 | 9634439 | 9637966 | SPTMK |
| PCE_F_Chro5G0054900.2 | PCE_Fruticosa_Chro5 | 9634439 | 9637966 | SPTMK |
| PCE_F_Chro5G0054900.3 | PCE_Fruticosa_Chro5 | 9634439 | 9637966 | SPTMK |
| PCE_F_Chro5G0054900.4 | PCE_Fruticosa_Chro5 | 9634439 | 9637966 | SPTMK |
| PCE_F_Chro5G0058300.1 | PCE_Fruticosa_Chro5 | 10084912 | 10089263 | SPTMK |
| PCE_F_Chro5G0058300.3 | PCE_Fruticosa_Chro5 | 10084912 | 10089263 | SPTMK |
| PCE_F_Chro5G0058700.1 | PCE_Fruticosa_Chro5 | 10144572 | 10147538 | SPTMK |
| PCE_F_Chro5G0058700.2 | PCE_Fruticosa_Chro5 | 10144572 | 10147538 | SPTMK |
| PCE_F_Chro5G0058700.3 | PCE_Fruticosa_Chro5 | 10144572 | 10147538 | SPTMK |
| PCE_F_Chro5G0058700.4 | PCE_Fruticosa_Chro5 | 10144572 | 10147538 | SPTMK |
| PCE_F_Chro5G0059200.1 | PCE_Fruticosa_Chro5 | 10303721 | 10307129 | SPTMK |
| PCE_F_Chro5G0059200.2 | PCE_Fruticosa_Chro5 | 10303721 | 10307129 | SPTMK |
| PCE_F_Chro5G0059200.3 | PCE_Fruticosa_Chro5 | 10303721 | 10307129 | SPTMK |
| PCE_F_Chro5G0059200.4 | PCE_Fruticosa_Chro5 | 10303721 | 10307129 | SPTMK |
| PCE_F_Chro5G0059200.5 | PCE_Fruticosa_Chro5 | 10303721 | 10307129 | SPTMK |
| PCE_F_Chro5G0060400.2 | PCE_Fruticosa_Chro5 | 10507667 | 10510825 | SPTMK |
| PCE_F_Chro5G0061300.1 | PCE_Fruticosa_Chro5 | 10590083 | 10593103 | SPTMK |
| PCE_F_Chro5G0061300.2 | PCE_Fruticosa_Chro5 | 10590083 | 10593103 | SPTMK |
| PCE_F_Chro5G0061300.3 | PCE_Fruticosa_Chro5 | 10590083 | 10593103 | SPTMK |
| PCE_F_Chro5G0061300.4 | PCE_Fruticosa_Chro5 | 10590083 | 10593103 | SPTMK |
| PCE_F_Chro5G0061300.5 | PCE_Fruticosa_Chro5 | 10590083 | 10593103 | SPTMK |
| PCE_F_Chro5G0067600.1 | PCE_Fruticosa_Chro5 | 11175345 | 11177676 | RLP |
| PCE_F_Chro5G0085700.1 | PCE_Fruticosa_Chro5 | 12848606 | 12868239 | TIR-NB-LRR |
| PCE_F_Chro5G0085700.2 | PCE_Fruticosa_Chro5 | 12848606 | 12868239 | TIR-NB-LRR |
| PCE_F_Chro5G0085800.3 | PCE_Fruticosa_Chro5 | 12874227 | 12880115 | TIR-NB-LRR |
| PCE_F_Chro5G0085800.1 | PCE_Fruticosa_Chro5 | 12874227 | 12880115 | TIR-NB-LRR |
| PCE_F_Chro5G0085800.2 | PCE_Fruticosa_Chro5 | 12874227 | 12880115 | TIR-NB-LRR |
| PCE_F_Chro5G0090200.1 | PCE_Fruticosa_Chro5 | 13210340 | 13214786 | RLK |
| PCE_F_Chro5G0094900.1 | PCE_Fruticosa_Chro5 | 13588585 | 13593477 | RLK |
| PCE_F_Chro5G0094900.4 | PCE_Fruticosa_Chro5 | 13588585 | 13593477 | RLK |
| PCE_F_Chro5G0100700.1 | PCE_Fruticosa_Chro5 | 14079657 | 14081561 | SPTMK |
| PCE_F_Chro5G0102400.1 | PCE_Fruticosa_Chro5 | 14305125 | 14308135 | SPTMK |
| PCE_F_Chro5G0102400.2 | PCE_Fruticosa_Chro5 | 14305125 | 14308135 | SPTMK |
| PCE_F_Chro5G0112400.1 | PCE_Fruticosa_Chro5 | 15104861 | 15109081 | RLK |
| PCE_F_Chro5G0159800.1 | PCE_Fruticosa_Chro5 | 18116763 | 18121042 | RLK |
| PCE_F_Chro5G0159800.2 | PCE_Fruticosa_Chro5 | 18116763 | 18121042 | RLK |
| PCE_F_Chro5G0159800.3 | PCE_Fruticosa_Chro5 | 18116763 | 18121042 | RLK |
| PCE_F_Chro5G0159800.4 | PCE_Fruticosa_Chro5 | 18116763 | 18121042 | RLK |
| PCE_F_Chro5G0166800.1 | PCE_Fruticosa_Chro5 | 18693337 | 18696113 | LysM |
| PCE_F_Chro5G0166800.2 | PCE_Fruticosa_Chro5 | 18693337 | 18696113 | LysM |
| PCE_F_Chro5G0168000.1 | PCE_Fruticosa_Chro5 | 18780768 | 18785238 | RLK |
| PCE_F_Chro5G0169200.1 | PCE_Fruticosa_Chro5 | 18836638 | 18840351 | RLP |
| PCE_F_Chro5G0169700.1 | PCE_Fruticosa_Chro5 | 18864007 | 18866557 | SPTMK |
| PCE_F_Chro5G0169700.2 | PCE_Fruticosa_Chro5 | 18864007 | 18866557 | SPTMK |
| PCE_F_Chro5G0169700.3 | PCE_Fruticosa_Chro5 | 18864007 | 18866557 | SPTMK |
| PCE_F_Chro5G0174300.1 | PCE_Fruticosa_Chro5 | 19225572 | 19227914 | RLK |
| PCE_F_Chro5G0177700.1 | PCE_Fruticosa_Chro5 | 19520253 | 19522443 | RLK |
| PCE_F_Chro5G0180100.1 | PCE_Fruticosa_Chro5 | 19639493 | 19642397 | LysM |
| PCE_F_Chro5G0180100.2 | PCE_Fruticosa_Chro5 | 19639493 | 19642397 | LysM |
| PCE_F_Chro5G0187100.1 | PCE_Fruticosa_Chro5 | 20128872 | 20132205 | RLK |
| PCE_F_Chro5G0197900.3 | PCE_Fruticosa_Chro5 | 20832968 | 20838207 | SPTMK |
| PCE_F_Chro5G0198000.1 | PCE_Fruticosa_Chro5 | 20839120 | 20845067 | SPTMK |
| PCE_F_Chro5G0201300.1 | PCE_Fruticosa_Chro5 | 21035174 | 21036917 | LysM |
| PCE_F_Chro5G0201300.2 | PCE_Fruticosa_Chro5 | 21035174 | 21036917 | LysM |
| PCE_F_Chro5G0201300.3 | PCE_Fruticosa_Chro5 | 21035174 | 21036914 | LysM |
| PCE_F_Chro5G0201300.4 | PCE_Fruticosa_Chro5 | 21035654 | 21036917 | LysM |
| PCE_F_Chro5G0207200.2 | PCE_Fruticosa_Chro5 | 21370256 | 21371753 | RLP |
| PCE_F_Chro6G0013900.1 | PCE_Fruticosa_Chro6 | 1055304 | 1062840 | SPTMK |
| PCE_F_Chro6G0014300.1 | PCE_Fruticosa_Chro6 | 1096300 | 1107564 | SPTMK |
| PCE_F_Chro6G0014300.2 | PCE_Fruticosa_Chro6 | 1096300 | 1107564 | SPTMK |
| PCE_F_Chro6G0014400.1 | PCE_Fruticosa_Chro6 | 1109070 | 1112642 | SPTMK |
| PCE_F_Chro6G0014500.1 | PCE_Fruticosa_Chro6 | 1115152 | 1117640 | SPTMK |
| PCE_F_Chro6G0017700.1 | PCE_Fruticosa_Chro6 | 1511431 | 1513824 | SPTMK |
| PCE_F_Chro6G0018000.1 | PCE_Fruticosa_Chro6 | 1534094 | 1536496 | SPTMK |
| PCE_F_Chro6G0018500.1 | PCE_Fruticosa_Chro6 | 1576750 | 1583787 | SPTMK |
| PCE_F_Chro6G0018700.1 | PCE_Fruticosa_Chro6 | 1591806 | 1594208 | SPTMK |
| PCE_F_Chro6G0018800.1 | PCE_Fruticosa_Chro6 | 1596523 | 1598933 | SPTMK |
| PCE_F_Chro6G0018800.2 | PCE_Fruticosa_Chro6 | 1596523 | 1598933 | SPTMK |
| PCE_F_Chro6G0018900.1 | PCE_Fruticosa_Chro6 | 1638461 | 1640889 | SPTMK |
| PCE_F_Chro6G0019500.2 | PCE_Fruticosa_Chro6 | 1679124 | 1687565 | SPTMK |
| PCE_F_Chro6G0019500.1 | PCE_Fruticosa_Chro6 | 1679160 | 1687565 | SPTMK |
| PCE_F_Chro6G0019500.3 | PCE_Fruticosa_Chro6 | 1679160 | 1681407 | SPTMK |
| PCE_F_Chro6G0019600.1 | PCE_Fruticosa_Chro6 | 1698016 | 1700406 | SPTMK |
| PCE_F_Chro6G0023100.1 | PCE_Fruticosa_Chro6 | 1944172 | 1946638 | SPTMK |
| PCE_F_Chro6G0035200.1 | PCE_Fruticosa_Chro6 | 2946122 | 2955226 | RLK |
| PCE_F_Chro6G0035200.2 | PCE_Fruticosa_Chro6 | 2946122 | 2955226 | RLK |
| PCE_F_Chro6G0048500.1 | PCE_Fruticosa_Chro6 | 3925402 | 3929943 | RLK |
| PCE_F_Chro6G0063400.1 | PCE_Fruticosa_Chro6 | 5064465 | 5068530 | RLK |
| PCE_F_Chro6G0063400.2 | PCE_Fruticosa_Chro6 | 5064465 | 5068515 | RLK |
| PCE_F_Chro6G0063400.3 | PCE_Fruticosa_Chro6 | 5064465 | 5068530 | RLK |
| PCE_F_Chro6G0063500.1 | PCE_Fruticosa_Chro6 | 5072607 | 5075744 | RLK |
| PCE_F_Chro6G0063500.3 | PCE_Fruticosa_Chro6 | 5072607 | 5075729 | RLK |
| PCE_F_Chro6G0063900.1 | PCE_Fruticosa_Chro6 | 5115340 | 5118965 | RLK |
| PCE_F_Chro6G0063900.3 | PCE_Fruticosa_Chro6 | 5115340 | 5118950 | RLK |
| PCE_F_Chro6G0088800.1 | PCE_Fruticosa_Chro6 | 7101021 | 7110496 | RLP |
| PCE_F_Chro6G0088800.2 | PCE_Fruticosa_Chro6 | 7101021 | 7110514 | RLP |
| PCE_F_Chro6G0089100.1 | PCE_Fruticosa_Chro6 | 7146560 | 7149956 | SPTMK |
| PCE_F_Chro6G0089300.1 | PCE_Fruticosa_Chro6 | 7161088 | 7164776 | SPTMK |
| PCE_F_Chro6G0089300.2 | PCE_Fruticosa_Chro6 | 7161088 | 7164776 | SPTMK |
| PCE_F_Chro6G0107000.1 | PCE_Fruticosa_Chro6 | 8598194 | 8603796 | SPTMK |
| PCE_F_Chro6G0109800.1 | PCE_Fruticosa_Chro6 | 8925551 | 8933403 | SPTMK |
| PCE_F_Chro6G0110500.1 | PCE_Fruticosa_Chro6 | 9048471 | 9050306 | LysM |
| PCE_F_Chro6G0124100.1 | PCE_Fruticosa_Chro6 | 10419996 | 10422263 | RLP |
| PCE_F_Chro6G0129800.1 | PCE_Fruticosa_Chro6 | 11097098 | 11106540 | RLK |
| PCE_F_Chro6G0130100.1 | PCE_Fruticosa_Chro6 | 11163774 | 11172970 | RLK |
| PCE_F_Chro6G0133200.1 | PCE_Fruticosa_Chro6 | 11556959 | 11571926 | RLK |
| PCE_F_Chro6G0134900.1 | PCE_Fruticosa_Chro6 | 12006207 | 12020478 | RLK |
| PCE_F_Chro6G0136100.1 | PCE_Fruticosa_Chro6 | 12163265 | 12174780 | RLP |
| PCE_F_Chro6G0140300.1 | PCE_Fruticosa_Chro6 | 12787417 | 12793203 | CC-NB-LRR |
| PCE_F_Chro6G0140300.2 | PCE_Fruticosa_Chro6 | 12787417 | 12793203 | CC-NB-LRR |
| PCE_F_Chro6G0147400.1 | PCE_Fruticosa_Chro6 | 13537535 | 13539884 | SPTMK |
| PCE_F_Chro6G0147400.3 | PCE_Fruticosa_Chro6 | 13537535 | 13539884 | SPTMK |
| PCE_F_Chro6G0147900.1 | PCE_Fruticosa_Chro6 | 13574814 | 13576804 | SPTMK |
| PCE_F_Chro6G0150200.2 | PCE_Fruticosa_Chro6 | 13785088 | 13787694 | SPTMK |
| PCE_F_Chro6G0150300.1 | PCE_Fruticosa_Chro6 | 13789659 | 13792187 | SPTMK |
| PCE_F_Chro6G0150700.1 | PCE_Fruticosa_Chro6 | 13869344 | 13871294 | SPTMK |
| PCE_F_Chro6G0151300.4 | PCE_Fruticosa_Chro6 | 13958086 | 13960587 | SPTMK |
| PCE_F_Chro6G0152600.1 | PCE_Fruticosa_Chro6 | 14175032 | 14177081 | SPTMK |
| PCE_F_Chro6G0152600.2 | PCE_Fruticosa_Chro6 | 14175032 | 14177081 | SPTMK |
| PCE_F_Chro6G0152600.4 | PCE_Fruticosa_Chro6 | 14175032 | 14177081 | SPTMK |
| PCE_F_Chro6G0153400.1 | PCE_Fruticosa_Chro6 | 14263451 | 14265886 | SPTMK |
| PCE_F_Chro6G0153400.4 | PCE_Fruticosa_Chro6 | 14263451 | 14265886 | SPTMK |
| PCE_F_Chro6G0154600.1 | PCE_Fruticosa_Chro6 | 14458829 | 14460875 | SPTMK |
| PCE_F_Chro6G0154600.2 | PCE_Fruticosa_Chro6 | 14458829 | 14460875 | SPTMK |
| PCE_F_Chro6G0154700.1 | PCE_Fruticosa_Chro6 | 14461147 | 14463806 | SPTMK |
| PCE_F_Chro6G0155400.1 | PCE_Fruticosa_Chro6 | 14574577 | 14576573 | SPTMK |
| PCE_F_Chro6G0156200.1 | PCE_Fruticosa_Chro6 | 14729081 | 14731983 | SPTMK |
| PCE_F_Chro6G0156200.2 | PCE_Fruticosa_Chro6 | 14729081 | 14731983 | SPTMK |
| PCE_F_Chro6G0157300.1 | PCE_Fruticosa_Chro6 | 14891694 | 14894488 | SPTMK |
| PCE_F_Chro6G0157300.2 | PCE_Fruticosa_Chro6 | 14891694 | 14894488 | SPTMK |
| PCE_F_Chro6G0157300.3 | PCE_Fruticosa_Chro6 | 14891694 | 14894488 | SPTMK |
| PCE_F_Chro6G0159500.1 | PCE_Fruticosa_Chro6 | 15101461 | 15106412 | RLK |
| PCE_F_Chro6G0159500.2 | PCE_Fruticosa_Chro6 | 15101461 | 15106412 | RLK |
| PCE_F_Chro6G0170300.1 | PCE_Fruticosa_Chro6 | 16960139 | 16963577 | TIR-NB-LRR |
| PCE_F_Chro6G0171900.1 | PCE_Fruticosa_Chro6 | 17179716 | 17187177 | RLK |
| PCE_F_Chro6G0177200.1 | PCE_Fruticosa_Chro6 | 18056709 | 18067086 | SPTMK |
| PCE_F_Chro6G0180200.1 | PCE_Fruticosa_Chro6 | 18503915 | 18507911 | RLK |
| PCE_F_Chro6G0180200.3 | PCE_Fruticosa_Chro6 | 18503915 | 18507911 | RLK |
| PCE_F_Chro6G0183900.1 | PCE_Fruticosa_Chro6 | 19280494 | 19283142 | SPTMK |
| PCE_F_Chro6G0184000.1 | PCE_Fruticosa_Chro6 | 19285101 | 19288283 | SPTMK |
| PCE_F_Chro6G0184100.1 | PCE_Fruticosa_Chro6 | 19289656 | 19292041 | SPTMK |
| PCE_F_Chro6G0184300.1 | PCE_Fruticosa_Chro6 | 19329832 | 19332159 | SPTMK |
| PCE_F_Chro6G0184800.1 | PCE_Fruticosa_Chro6 | 19399497 | 19401825 | SPTMK |
| PCE_F_Chro6G0194000.1 | PCE_Fruticosa_Chro6 | 21911384 | 21915836 | RLK |
| PCE_F_Chro6G0196700.1 | PCE_Fruticosa_Chro6 | 22386708 | 22388954 | RLP |
| PCE_F_Chro6G0196700.2 | PCE_Fruticosa_Chro6 | 22386708 | 22388954 | RLP |
| PCE_F_Chro6G0206100.1 | PCE_Fruticosa_Chro6 | 23914605 | 23917451 | SPTMK |
| PCE_F_Chro6G0210100.1 | PCE_Fruticosa_Chro6 | 25322496 | 25325347 | SPTMK |
| PCE_F_Chro6G0210100.2 | PCE_Fruticosa_Chro6 | 25322496 | 25325347 | SPTMK |
| PCE_F_Chro6G0210100.3 | PCE_Fruticosa_Chro6 | 25322508 | 25325347 | SPTMK |
| PCE_F_Chro6G0210100.4 | PCE_Fruticosa_Chro6 | 25322508 | 25325347 | SPTMK |
| PCE_F_Chro6G0210300.1 | PCE_Fruticosa_Chro6 | 25412047 | 25414905 | SPTMK |
| PCE_F_Chro6G0210300.3 | PCE_Fruticosa_Chro6 | 25412047 | 25414905 | SPTMK |
| PCE_F_Chro6G0210300.5 | PCE_Fruticosa_Chro6 | 25412047 | 25414905 | SPTMK |
| PCE_F_Chro6G0210300.6 | PCE_Fruticosa_Chro6 | 25412047 | 25414905 | SPTMK |
| PCE_F_Chro6G0210300.4 | PCE_Fruticosa_Chro6 | 25412059 | 25414905 | SPTMK |
| PCE_F_Chro6G0210300.7 | PCE_Fruticosa_Chro6 | 25412059 | 25414905 | SPTMK |
| PCE_F_Chro6G0215300.1 | PCE_Fruticosa_Chro6 | 25856854 | 25868617 | RLK |
| PCE_F_Chro6G0215300.2 | PCE_Fruticosa_Chro6 | 25856854 | 25868617 | RLK |
| PCE_F_Chro6G0215900.1 | PCE_Fruticosa_Chro6 | 25919762 | 25923408 | RLP |
| PCE_F_Chro6G0217100.1 | PCE_Fruticosa_Chro6 | 26028277 | 26031769 | RLP |
| PCE_F_Chro6G0217800.1 | PCE_Fruticosa_Chro6 | 26083370 | 26086693 | SPTMK |
| PCE_F_Chro6G0217800.2 | PCE_Fruticosa_Chro6 | 26083370 | 26086693 | SPTMK |
| PCE_F_Chro6G0217800.5 | PCE_Fruticosa_Chro6 | 26084107 | 26086693 | SPTMK |
| PCE_F_Chro6G0223100.1 | PCE_Fruticosa_Chro6 | 26650158 | 26662790 | CC-NB-LRR |
| PCE_F_Chro6G0224100.1 | PCE_Fruticosa_Chro6 | 26809511 | 26817451 | CC-NB-LRR |
| PCE_F_Chro6G0224400.1 | PCE_Fruticosa_Chro6 | 26869003 | 26871715 | CC-NB-LRR |
| PCE_F_Chro6G0224600.1 | PCE_Fruticosa_Chro6 | 26903140 | 26905851 | CC-NB-LRR |
| PCE_F_Chro6G0225200.1 | PCE_Fruticosa_Chro6 | 26999524 | 27002223 | CC-NB-LRR |
| PCE_F_Chro6G0232500.1 | PCE_Fruticosa_Chro6 | 27733917 | 27736636 | CC-NB-LRR |
| PCE_F_Chro6G0232700.1 | PCE_Fruticosa_Chro6 | 27750832 | 27752907 | SPTMK |
| PCE_F_Chro6G0233000.1 | PCE_Fruticosa_Chro6 | 27779862 | 27781820 | SPTMK |
| PCE_F_Chro6G0233700.1 | PCE_Fruticosa_Chro6 | 27858510 | 27867197 | RLK |
| PCE_F_Chro6G0236500.1 | PCE_Fruticosa_Chro6 | 28198711 | 28202758 | RLK |
| PCE_F_Chro6G0236900.1 | PCE_Fruticosa_Chro6 | 28233554 | 28237641 | RLK |
| PCE_F_Chro6G0240300.1 | PCE_Fruticosa_Chro6 | 28532130 | 28533844 | RLP |
| PCE_F_Chro6G0252600.1 | PCE_Fruticosa_Chro6 | 29592951 | 29600370 | SPTMK |
| PCE_F_Chro6G0252700.1 | PCE_Fruticosa_Chro6 | 29601373 | 29603754 | SPTMK |
| PCE_F_Chro6G0252900.1 | PCE_Fruticosa_Chro6 | 29617170 | 29619701 | SPTMK |
| PCE_F_Chro6G0253000.1 | PCE_Fruticosa_Chro6 | 29622328 | 29643079 | SPTMK |
| PCE_F_Chro6G0253200.1 | PCE_Fruticosa_Chro6 | 29631427 | 29633844 | SPTMK |
| PCE_F_Chro6G0253400.1 | PCE_Fruticosa_Chro6 | 29670333 | 29673104 | SPTMK |
| PCE_F_Chro6G0253500.1 | PCE_Fruticosa_Chro6 | 29676043 | 29678323 | SPTMK |
| PCE_F_Chro6G0253600.1 | PCE_Fruticosa_Chro6 | 29679951 | 29682642 | SPTMK |
| PCE_F_Chro6G0254400.1 | PCE_Fruticosa_Chro6 | 29753533 | 29757125 | RLK |
| PCE_F_Chro6G0254400.2 | PCE_Fruticosa_Chro6 | 29753533 | 29757125 | RLK |
| PCE_F_Chro6G0256600.1 | PCE_Fruticosa_Chro6 | 29869475 | 29873231 | RLK |
| PCE_F_Chro6G0256600.2 | PCE_Fruticosa_Chro6 | 29869475 | 29873231 | RLK |
| PCE_F_Chro6G0263500.1 | PCE_Fruticosa_Chro6 | 30366249 | 30373093 | SPTMK |
| PCE_F_Chro6G0267500.1 | PCE_Fruticosa_Chro6 | 30673582 | 30680204 | RLK |
| PCE_F_Chro6G0267500.2 | PCE_Fruticosa_Chro6 | 30673582 | 30680599 | RLK |
| PCE_F_Chro6G0275000.1 | PCE_Fruticosa_Chro6 | 31099377 | 31103250 | RLK |
| PCE_F_Chro6G0275400.1 | PCE_Fruticosa_Chro6 | 31175792 | 31179153 | RLK |
| PCE_F_Chro6G0275800.1 | PCE_Fruticosa_Chro6 | 31201392 | 31205478 | RLK |
| PCE_F_Chro6G0275900.1 | PCE_Fruticosa_Chro6 | 31212805 | 31216353 | RLK |
| PCE_F_Chro6G0288500.1 | PCE_Fruticosa_Chro6 | 31976466 | 31978430 | SPTMK |
| PCE_F_Chro6G0288600.1 | PCE_Fruticosa_Chro6 | 31995688 | 31997694 | SPTMK |
| PCE_F_Chro6G0288600.2 | PCE_Fruticosa_Chro6 | 31995688 | 31997685 | SPTMK |
| PCE_F_Chro6G0288700.1 | PCE_Fruticosa_Chro6 | 31998997 | 32000964 | SPTMK |
| PCE_F_Chro6G0288700.3 | PCE_Fruticosa_Chro6 | 31998997 | 32000964 | SPTMK |
| PCE_F_Chro6G0288800.1 | PCE_Fruticosa_Chro6 | 32002269 | 32004467 | SPTMK |
| PCE_F_Chro6G0288900.1 | PCE_Fruticosa_Chro6 | 32008447 | 32010475 | SPTMK |
| PCE_F_Chro6G0288900.4 | PCE_Fruticosa_Chro6 | 32008447 | 32010475 | SPTMK |
| PCE_F_Chro6G0289000.1 | PCE_Fruticosa_Chro6 | 32014344 | 32017040 | SPTMK |
| PCE_F_Chro6G0289800.1 | PCE_Fruticosa_Chro6 | 32060891 | 32063459 | SPTMK |
| PCE_F_Chro6G0289800.2 | PCE_Fruticosa_Chro6 | 32060891 | 32063459 | SPTMK |
| PCE_F_Chro6G0289900.1 | PCE_Fruticosa_Chro6 | 32064608 | 32066762 | SPTMK |
| PCE_F_Chro6G0289900.2 | PCE_Fruticosa_Chro6 | 32064608 | 32066762 | SPTMK |
| PCE_F_Chro6G0290100.1 | PCE_Fruticosa_Chro6 | 32074938 | 32076935 | SPTMK |
| PCE_F_Chro6G0290100.3 | PCE_Fruticosa_Chro6 | 32074938 | 32076935 | SPTMK |
| PCE_F_Chro6G0291100.1 | PCE_Fruticosa_Chro6 | 32153520 | 32157115 | SPTMK |
| PCE_F_Chro6G0291100.4 | PCE_Fruticosa_Chro6 | 32153520 | 32157115 | SPTMK |
| PCE_F_Chro6G0291100.5 | PCE_Fruticosa_Chro6 | 32153520 | 32157115 | SPTMK |
| PCE_F_Chro6G0291200.1 | PCE_Fruticosa_Chro6 | 32168527 | 32170920 | SPTMK |
| PCE_F_Chro6G0291300.1 | PCE_Fruticosa_Chro6 | 32202826 | 32205364 | SPTMK |
| PCE_F_Chro6G0292100.1 | PCE_Fruticosa_Chro6 | 32338949 | 32341064 | SPTMK |
| PCE_F_Chro6G0292100.4 | PCE_Fruticosa_Chro6 | 32338949 | 32341064 | SPTMK |
| PCE_F_Chro6G0292100.5 | PCE_Fruticosa_Chro6 | 32338949 | 32341064 | SPTMK |
| PCE_F_Chro6G0293600.1 | PCE_Fruticosa_Chro6 | 32446124 | 32448562 | SPTMK |
| PCE_F_Chro6G0293700.1 | PCE_Fruticosa_Chro6 | 32449219 | 32451658 | SPTMK |
| PCE_F_Chro6G0293900.1 | PCE_Fruticosa_Chro6 | 32456273 | 32458654 | SPTMK |
| PCE_F_Chro6G0294000.1 | PCE_Fruticosa_Chro6 | 32462306 | 32468911 | SPTMK |
| PCE_F_Chro6G0294300.1 | PCE_Fruticosa_Chro6 | 32507046 | 32509424 | SPTMK |
| PCE_F_Chro6G0305400.1 | PCE_Fruticosa_Chro6 | 33385221 | 33388816 | RLK |
| PCE_F_Chro6G0305400.2 | PCE_Fruticosa_Chro6 | 33385221 | 33387992 | RLP |
| PCE_F_Chro6G0305400.3 | PCE_Fruticosa_Chro6 | 33385221 | 33393413 | RLK |
| PCE_F_Chro6G0305600.1 | PCE_Fruticosa_Chro6 | 33399957 | 33404016 | RLK |
| PCE_F_Chro6G0305700.1 | PCE_Fruticosa_Chro6 | 33404213 | 33407682 | RLK |
| PCE_F_Chro6G0306100.1 | PCE_Fruticosa_Chro6 | 33437828 | 33455721 | RLK |
| PCE_F_Chro6G0306600.1 | PCE_Fruticosa_Chro6 | 33456861 | 33460203 | RLK |
| PCE_F_Chro6G0314800.1 | PCE_Fruticosa_Chro6 | 33994030 | 33996720 | RLP |
| PCE_F_Chro6G0314900.1 | PCE_Fruticosa_Chro6 | 34007213 | 34010174 | RLP |
| PCE_F_Chro6G0315000.1 | PCE_Fruticosa_Chro6 | 34046093 | 34049151 | RLP |
| PCE_F_Chro6G0315200.1 | PCE_Fruticosa_Chro6 | 34083694 | 34086921 | RLP |
| PCE_F_Chro6G0315400.1 | PCE_Fruticosa_Chro6 | 34136991 | 34140829 | RLP |
| PCE_F_Chro6G0316600.1 | PCE_Fruticosa_Chro6 | 34205261 | 34207289 | SPTMK |
| PCE_F_Chro6G0318800.1 | PCE_Fruticosa_Chro6 | 34334509 | 34337332 | RLK |
| PCE_F_Chro6G0318800.2 | PCE_Fruticosa_Chro6 | 34334509 | 34337332 | RLK |
| PCE_F_Chro6G0318800.3 | PCE_Fruticosa_Chro6 | 34334509 | 34337332 | RLK |
| PCE_F_Chro6G0318800.4 | PCE_Fruticosa_Chro6 | 34334509 | 34337332 | RLK |
| PCE_F_Chro6G0327500.1 | PCE_Fruticosa_Chro6 | 34989930 | 34993261 | RLK |
| PCE_F_Chro6G0327500.2 | PCE_Fruticosa_Chro6 | 34990606 | 34993261 | RLP |
| PCE_F_Chro6G0329800.1 | PCE_Fruticosa_Chro6 | 35152123 | 35155437 | SPTMK |
| PCE_F_Chro6G0330300.1 | PCE_Fruticosa_Chro6 | 35170457 | 35174847 | RLK |
| PCE_F_Chro6G0331100.1 | PCE_Fruticosa_Chro6 | 35213628 | 35220188 | RLK |
| PCE_F_Chro6G0331100.2 | PCE_Fruticosa_Chro6 | 35213628 | 35220188 | RLK |
| PCE_F_Chro6G0331100.3 | PCE_Fruticosa_Chro6 | 35213628 | 35220188 | RLK |
| PCE_F_Chro6G0331100.5 | PCE_Fruticosa_Chro6 | 35213628 | 35220188 | RLK |
| PCE_F_Chro6G0331300.1 | PCE_Fruticosa_Chro6 | 35230988 | 35236899 | RLK |
| PCE_F_Chro6G0331300.2 | PCE_Fruticosa_Chro6 | 35230997 | 35236899 | RLK |
| PCE_F_Chro6G0335100.1 | PCE_Fruticosa_Chro6 | 35436205 | 35442250 | RLK |
| PCE_F_Chro6G0335100.2 | PCE_Fruticosa_Chro6 | 35436205 | 35442250 | RLK |
| PCE_F_Chro6G0335100.3 | PCE_Fruticosa_Chro6 | 35436205 | 35442250 | RLK |
| PCE_F_Chro6G0335600.1 | PCE_Fruticosa_Chro6 | 35483045 | 35493251 | RLK |
| PCE_F_Chro6G0335600.2 | PCE_Fruticosa_Chro6 | 35483045 | 35493251 | RLK |
| PCE_F_Chro6G0335900.1 | PCE_Fruticosa_Chro6 | 35514147 | 35535902 | RLK |
| PCE_F_Chro6G0336300.1 | PCE_Fruticosa_Chro6 | 35549926 | 35562545 | RLK |
| PCE_F_Chro6G0336300.2 | PCE_Fruticosa_Chro6 | 35549926 | 35562545 | RLK |
| PCE_F_Chro6G0336300.3 | PCE_Fruticosa_Chro6 | 35549926 | 35562545 | RLK |
| PCE_F_Chro6G0336300.4 | PCE_Fruticosa_Chro6 | 35549926 | 35562545 | RLK |
| PCE_F_Chro6G0359100.1 | PCE_Fruticosa_Chro6 | 36981948 | 36983344 | LysM |
| PCE_F_Chro6G0366400.1 | PCE_Fruticosa_Chro6 | 37364217 | 37366081 | SPTMK |
| PCE_F_Chro6G0366400.2 | PCE_Fruticosa_Chro6 | 37364217 | 37366093 | SPTMK |
| PCE_F_Chro6G0366500.1 | PCE_Fruticosa_Chro6 | 37368101 | 37370038 | SPTMK |
| PCE_F_Chro6G0379300.1 | PCE_Fruticosa_Chro6 | 38157342 | 38159975 | SPTMK |
| PCE_F_Chro6G0386600.1 | PCE_Fruticosa_Chro6 | 38574157 | 38578396 | RLK |
| PCE_F_Chro6G0387100.1 | PCE_Fruticosa_Chro6 | 38607408 | 38611051 | SPTMK |
| PCE_F_Chro6G0387100.2 | PCE_Fruticosa_Chro6 | 38607408 | 38611051 | SPTMK |
| PCE_F_Chro6G0387200.1 | PCE_Fruticosa_Chro6 | 38611386 | 38613684 | SPTMK |
| PCE_F_Chro6G0387800.1 | PCE_Fruticosa_Chro6 | 38634852 | 38636998 | LysM |
| PCE_F_Chro6G0390400.1 | PCE_Fruticosa_Chro6 | 38764986 | 38767702 | RLP |
| PCE_F_Chro6G0398100.1 | PCE_Fruticosa_Chro6 | 39410802 | 39413206 | SPTMK |
| PCE_F_Chro7G0004000.1 | PCE_Fruticosa_Chro7 | 341535 | 343676 | RLP |
| PCE_F_Chro7G0006100.1 | PCE_Fruticosa_Chro7 | 425771 | 429964 | RLK |
| PCE_F_Chro7G0009100.1 | PCE_Fruticosa_Chro7 | 630481 | 634681 | RLK |
| PCE_F_Chro7G0011600.1 | PCE_Fruticosa_Chro7 | 749472 | 751589 | RLP |
| PCE_F_Chro7G0027000.1 | PCE_Fruticosa_Chro7 | 1681902 | 1685416 | SPTMK |
| PCE_F_Chro7G0032600.1 | PCE_Fruticosa_Chro7 | 2012480 | 2015948 | RLK |
| PCE_F_Chro7G0035900.1 | PCE_Fruticosa_Chro7 | 2168525 | 2171748 | SPTMK |
| PCE_F_Chro7G0046300.1 | PCE_Fruticosa_Chro7 | 2762067 | 2766342 | SPTMK |
| PCE_F_Chro7G0046300.2 | PCE_Fruticosa_Chro7 | 2762067 | 2766342 | SPTMK |
| PCE_F_Chro7G0051000.1 | PCE_Fruticosa_Chro7 | 3051727 | 3058679 | SPTMK |
| PCE_F_Chro7G0051000.2 | PCE_Fruticosa_Chro7 | 3051727 | 3058667 | SPTMK |
| PCE_F_Chro7G0051200.1 | PCE_Fruticosa_Chro7 | 3072272 | 3077644 | SPTMK |
| PCE_F_Chro7G0051200.2 | PCE_Fruticosa_Chro7 | 3072272 | 3077644 | SPTMK |
| PCE_F_Chro7G0051200.5 | PCE_Fruticosa_Chro7 | 3072272 | 3077644 | SPTMK |
| PCE_F_Chro7G0051300.1 | PCE_Fruticosa_Chro7 | 3082445 | 3086392 | SPTMK |
| PCE_F_Chro7G0053800.1 | PCE_Fruticosa_Chro7 | 3266505 | 3269078 | RLK |
| PCE_F_Chro7G0065900.1 | PCE_Fruticosa_Chro7 | 4302494 | 4305822 | SPTMK |
| PCE_F_Chro7G0065900.2 | PCE_Fruticosa_Chro7 | 4302494 | 4305822 | SPTMK |
| PCE_F_Chro7G0070000.1 | PCE_Fruticosa_Chro7 | 4521246 | 4525489 | SPTMK |
| PCE_F_Chro7G0087800.1 | PCE_Fruticosa_Chro7 | 5578478 | 5582251 | RLK |
| PCE_F_Chro7G0094000.1 | PCE_Fruticosa_Chro7 | 5911430 | 5915648 | CC-NB-LRR |
| PCE_F_Chro7G0107500.1 | PCE_Fruticosa_Chro7 | 6671040 | 6675850 | LysM |
| PCE_F_Chro7G0107600.1 | PCE_Fruticosa_Chro7 | 6676581 | 6678491 | SPTMK |
| PCE_F_Chro7G0107800.1 | PCE_Fruticosa_Chro7 | 6683654 | 6685516 | LysM |
| PCE_F_Chro7G0110400.1 | PCE_Fruticosa_Chro7 | 6831828 | 6836867 | SPTMK |
| PCE_F_Chro7G0116100.1 | PCE_Fruticosa_Chro7 | 7237396 | 7248733 | SPTMK |
| PCE_F_Chro7G0121200.1 | PCE_Fruticosa_Chro7 | 7560644 | 7564419 | RPW8-NB-LRR |
| PCE_F_Chro7G0121400.1 | PCE_Fruticosa_Chro7 | 7569141 | 7572751 | RPW8-NB-LRR |
| PCE_F_Chro7G0121400.3 | PCE_Fruticosa_Chro7 | 7569141 | 7572751 | RPW8-NB-LRR |
| PCE_F_Chro7G0121500.1 | PCE_Fruticosa_Chro7 | 7588288 | 7592363 | RPW8-NB-LRR |
| PCE_F_Chro7G0121700.1 | PCE_Fruticosa_Chro7 | 7625682 | 7629326 | RPW8-NB-LRR |
| PCE_F_Chro7G0121700.2 | PCE_Fruticosa_Chro7 | 7625682 | 7629326 | RPW8-NB-LRR |
| PCE_F_Chro7G0121900.1 | PCE_Fruticosa_Chro7 | 7655645 | 7658939 | RPW8-NB-LRR |
| PCE_F_Chro7G0122000.1 | PCE_Fruticosa_Chro7 | 7659384 | 7666854 | RPW8-NB-LRR |
| PCE_F_Chro7G0122200.1 | PCE_Fruticosa_Chro7 | 7672312 | 7675462 | RPW8-NB-LRR |
| PCE_F_Chro7G0122300.1 | PCE_Fruticosa_Chro7 | 7677771 | 7682749 | RPW8-NB-LRR |
| PCE_F_Chro7G0122300.2 | PCE_Fruticosa_Chro7 | 7677771 | 7682749 | RPW8-NB-LRR |
| PCE_F_Chro7G0122300.3 | PCE_Fruticosa_Chro7 | 7677771 | 7682749 | RPW8-NB-LRR |
| PCE_F_Chro7G0122400.1 | PCE_Fruticosa_Chro7 | 7683799 | 7686967 | RPW8-NB-LRR |
| PCE_F_Chro7G0122500.1 | PCE_Fruticosa_Chro7 | 7700672 | 7703881 | RPW8-NB-LRR |
| PCE_F_Chro7G0122600.1 | PCE_Fruticosa_Chro7 | 7708129 | 7711680 | RPW8-NB-LRR |
| PCE_F_Chro7G0142400.1 | PCE_Fruticosa_Chro7 | 9171908 | 9177224 | SPTMK |
| PCE_F_Chro7G0143400.1 | PCE_Fruticosa_Chro7 | 9240101 | 9242819 | RLK |
| PCE_F_Chro7G0173300.1 | PCE_Fruticosa_Chro7 | 11676754 | 11680930 | RLK |
| PCE_F_Chro7G0189700.1 | PCE_Fruticosa_Chro7 | 13677234 | 13678694 | SPTMK |
| PCE_F_Chro7G0191900.1 | PCE_Fruticosa_Chro7 | 13892379 | 13897577 | RLK |
| PCE_F_Chro7G0191900.2 | PCE_Fruticosa_Chro7 | 13892379 | 13897577 | RLK |
| PCE_F_Chro7G0195300.1 | PCE_Fruticosa_Chro7 | 14237337 | 14240431 | RLK |
| PCE_F_Chro7G0198700.1 | PCE_Fruticosa_Chro7 | 14603150 | 14607303 | SPTMK |
| PCE_F_Chro7G0198800.1 | PCE_Fruticosa_Chro7 | 14623638 | 14632886 | TIR-NB-LRR |
| PCE_F_Chro7G0201800.1 | PCE_Fruticosa_Chro7 | 14954609 | 14958795 | RLK |
| PCE_F_Chro7G0202100.1 | PCE_Fruticosa_Chro7 | 14978569 | 14981484 | CC-NB-LRR |
| PCE_F_Chro7G0205500.1 | PCE_Fruticosa_Chro7 | 15289145 | 15292012 | RLP |
| PCE_F_Chro7G0216900.1 | PCE_Fruticosa_Chro7 | 17251313 | 17257505 | CC-NB-LRR |
| PCE_F_Chro7G0227700.1 | PCE_Fruticosa_Chro7 | 18588434 | 18592403 | RLK |
| PCE_F_Chro7G0231800.1 | PCE_Fruticosa_Chro7 | 19280223 | 19282729 | CC-NB-LRR |
| PCE_F_Chro7G0235300.1 | PCE_Fruticosa_Chro7 | 19950467 | 19964355 | TIR-NB-LRR |
| PCE_F_Chro7G0235300.2 | PCE_Fruticosa_Chro7 | 19950467 | 19964355 | TIR-NB-LRR |
| PCE_F_Chro7G0239000.1 | PCE_Fruticosa_Chro7 | 20661908 | 20664654 | RLP |
| PCE_F_Chro7G0239200.1 | PCE_Fruticosa_Chro7 | 20686752 | 20689877 | RLP |
| PCE_F_Chro7G0243700.1 | PCE_Fruticosa_Chro7 | 22012042 | 22029618 | CC-NB-LRR |
| PCE_F_Chro7G0252000.1 | PCE_Fruticosa_Chro7 | 24980807 | 24982975 | RLP |
| PCE_F_Chro7G0254600.1 | PCE_Fruticosa_Chro7 | 25745387 | 25749858 | SPTMK |
| PCE_F_Chro7G0257300.1 | PCE_Fruticosa_Chro7 | 26221583 | 26229541 | TIR-NB-LRR |
| PCE_F_Chro7G0272200.1 | PCE_Fruticosa_Chro7 | 28612089 | 28619229 | RLK |
| PCE_F_Chro7G0272200.2 | PCE_Fruticosa_Chro7 | 28612089 | 28619229 | RLK |
| PCE_F_Chro7G0272200.3 | PCE_Fruticosa_Chro7 | 28612089 | 28619229 | RLK |
| PCE_F_Chro7G0272900.1 | PCE_Fruticosa_Chro7 | 28712270 | 28713721 | CC-NB-LRR |
| PCE_F_Chro7G0279300.1 | PCE_Fruticosa_Chro7 | 29904899 | 29912720 | TIR-NB-LRR |
| PCE_F_Chro7G0293800.1 | PCE_Fruticosa_Chro7 | 33088883 | 33093926 | SPTMK |
| PCE_F_Chro7G0293800.3 | PCE_Fruticosa_Chro7 | 33088883 | 33093926 | SPTMK |
| PCE_F_Chro7G0300800.1 | PCE_Fruticosa_Chro7 | 39419366 | 39423549 | SPTMK |
| PCE_F_Chro7G0300800.2 | PCE_Fruticosa_Chro7 | 39419366 | 39423549 | SPTMK |
| PCE_F_Chro8G0001000.1 | PCE_Fruticosa_Chro8 | 437276 | 442321 | TIR-NB-LRR |
| PCE_F_Chro8G0001000.2 | PCE_Fruticosa_Chro8 | 437276 | 442321 | TIR-NB-LRR |
| PCE_F_Chro8G0003100.1 | PCE_Fruticosa_Chro8 | 1221672 | 1224496 | SPTMK |
| PCE_F_Chro8G0003300.1 | PCE_Fruticosa_Chro8 | 1237629 | 1239691 | RLP |
| PCE_F_Chro8G0005600.1 | PCE_Fruticosa_Chro8 | 1618198 | 1626202 | RLK |
| PCE_F_Chro8G0009300.1 | PCE_Fruticosa_Chro8 | 2253843 | 2255717 | RLP |
| PCE_F_Chro8G0009600.1 | PCE_Fruticosa_Chro8 | 2266658 | 2269512 | SPTMK |
| PCE_F_Chro8G0011200.1 | PCE_Fruticosa_Chro8 | 2551992 | 2554605 | RLP |
| PCE_F_Chro8G0011300.1 | PCE_Fruticosa_Chro8 | 2559580 | 2563187 | RLP |
| PCE_F_Chro8G0011400.1 | PCE_Fruticosa_Chro8 | 2690045 | 2693004 | RLP |
| PCE_F_Chro8G0012200.1 | PCE_Fruticosa_Chro8 | 2976627 | 2981010 | TIR-NB-LRR |
| PCE_F_Chro8G0024000.1 | PCE_Fruticosa_Chro8 | 6004183 | 6009480 | TIR-NB-LRR |
| PCE_F_Chro8G0024800.1 | PCE_Fruticosa_Chro8 | 6090542 | 6097030 | TIR-NB-LRR |
| PCE_F_Chro8G0024800.2 | PCE_Fruticosa_Chro8 | 6090542 | 6097030 | TIR-NB-LRR |
| PCE_F_Chro8G0025000.1 | PCE_Fruticosa_Chro8 | 6118906 | 6124427 | TIR-NB-LRR |
| PCE_F_Chro8G0025000.2 | PCE_Fruticosa_Chro8 | 6118906 | 6124427 | TIR-NB-LRR |
| PCE_F_Chro8G0025000.3 | PCE_Fruticosa_Chro8 | 6118906 | 6124427 | TIR-NB-LRR |
| PCE_F_Chro8G0025500.1 | PCE_Fruticosa_Chro8 | 6165757 | 6171840 | TIR-NB-LRR |
| PCE_F_Chro8G0025500.2 | PCE_Fruticosa_Chro8 | 6165757 | 6171840 | TIR-NB-LRR |
| PCE_F_Chro8G0025500.3 | PCE_Fruticosa_Chro8 | 6165757 | 6171840 | TIR-NB-LRR |
| PCE_F_Chro8G0027700.2 | PCE_Fruticosa_Chro8 | 6395362 | 6399254 | RLK |
| PCE_F_Chro8G0027700.1 | PCE_Fruticosa_Chro8 | 6395362 | 6399254 | RLK |
| PCE_F_Chro8G0029100.1 | PCE_Fruticosa_Chro8 | 6838784 | 6844505 | TIR-NB-LRR |
| PCE_F_Chro8G0030300.1 | PCE_Fruticosa_Chro8 | 7017503 | 7020850 | SPTMK |
| PCE_F_Chro8G0030300.3 | PCE_Fruticosa_Chro8 | 7017503 | 7020850 | SPTMK |
| PCE_F_Chro8G0032100.1 | PCE_Fruticosa_Chro8 | 7336304 | 7343378 | SPTMK |
| PCE_F_Chro8G0038100.1 | PCE_Fruticosa_Chro8 | 8065975 | 8071281 | TIR-NB-LRR |
| PCE_F_Chro8G0038100.2 | PCE_Fruticosa_Chro8 | 8065975 | 8071281 | TIR-NB-LRR |
| PCE_F_Chro8G0038100.4 | PCE_Fruticosa_Chro8 | 8065975 | 8071281 | TIR-NB-LRR |
| PCE_F_Chro8G0038900.1 | PCE_Fruticosa_Chro8 | 8163515 | 8169463 | TIR-NB-LRR |
| PCE_F_Chro8G0038900.2 | PCE_Fruticosa_Chro8 | 8163515 | 8169463 | TIR-NB-LRR |
| PCE_F_Chro8G0039200.4 | PCE_Fruticosa_Chro8 | 8193905 | 8199159 | TIR-NB-LRR |
| PCE_F_Chro8G0039200.1 | PCE_Fruticosa_Chro8 | 8193905 | 8199159 | TIR-NB-LRR |
| PCE_F_Chro8G0039200.2 | PCE_Fruticosa_Chro8 | 8193905 | 8199159 | TIR-NB-LRR |
| PCE_F_Chro8G0039200.3 | PCE_Fruticosa_Chro8 | 8193905 | 8199159 | TIR-NB-LRR |
| PCE_F_Chro8G0039200.7 | PCE_Fruticosa_Chro8 | 8193905 | 8199159 | TIR-NB-LRR |
| PCE_F_Chro8G0039200.8 | PCE_Fruticosa_Chro8 | 8193905 | 8199159 | TIR-NB-LRR |
| PCE_F_Chro8G0042200.1 | PCE_Fruticosa_Chro8 | 8517081 | 8524191 | TIR-NB-LRR |
| PCE_F_Chro8G0042200.2 | PCE_Fruticosa_Chro8 | 8517081 | 8524191 | TIR-NB-LRR |
| PCE_F_Chro8G0042200.3 | PCE_Fruticosa_Chro8 | 8517081 | 8524191 | TIR-NB-LRR |
| PCE_F_Chro8G0042200.7 | PCE_Fruticosa_Chro8 | 8517081 | 8524191 | TIR-NB-LRR |
| PCE_F_Chro8G0042200.4 | PCE_Fruticosa_Chro8 | 8518136 | 8524191 | TIR-NB-LRR |
| PCE_F_Chro8G0046900.1 | PCE_Fruticosa_Chro8 | 9372963 | 9375209 | RLP |
| PCE_F_Chro8G0047400.1 | PCE_Fruticosa_Chro8 | 9534984 | 9538446 | TIR-NB-LRR |
| PCE_F_Chro8G0047400.2 | PCE_Fruticosa_Chro8 | 9534996 | 9538446 | TIR-NB-LRR |
| PCE_F_Chro8G0047900.1 | PCE_Fruticosa_Chro8 | 9628465 | 9632636 | TIR-NB-LRR |
| PCE_F_Chro8G0047900.2 | PCE_Fruticosa_Chro8 | 9629314 | 9632636 | TIR-NB-LRR |
| PCE_F_Chro8G0048000.1 | PCE_Fruticosa_Chro8 | 9636930 | 9640831 | TIR-NB-LRR |
| PCE_F_Chro8G0048200.1 | PCE_Fruticosa_Chro8 | 9737948 | 9741915 | TIR-NB-LRR |
| PCE_F_Chro8G0048200.2 | PCE_Fruticosa_Chro8 | 9738555 | 9741915 | TIR-NB-LRR |
| PCE_F_Chro8G0048600.1 | PCE_Fruticosa_Chro8 | 9798314 | 9802238 | TIR-NB-LRR |
| PCE_F_Chro8G0048600.2 | PCE_Fruticosa_Chro8 | 9799036 | 9802238 | TIR-NB-LRR |
| PCE_F_Chro8G0048800.1 | PCE_Fruticosa_Chro8 | 9832764 | 9837121 | TIR-NB-LRR |
| PCE_F_Chro8G0048800.2 | PCE_Fruticosa_Chro8 | 9833656 | 9837121 | TIR-NB-LRR |
| PCE_F_Chro8G0051600.1 | PCE_Fruticosa_Chro8 | 10187204 | 10189485 | SPTMK |
| PCE_F_Chro8G0051600.2 | PCE_Fruticosa_Chro8 | 10187204 | 10189530 | SPTMK |
| PCE_F_Chro8G0053000.1 | PCE_Fruticosa_Chro8 | 10464912 | 10466936 | SPTMK |
| PCE_F_Chro8G0053200.1 | PCE_Fruticosa_Chro8 | 10495794 | 10498013 | SPTMK |
| PCE_F_Chro8G0053300.1 | PCE_Fruticosa_Chro8 | 10500821 | 10502983 | SPTMK |
| PCE_F_Chro8G0054100.1 | PCE_Fruticosa_Chro8 | 10599667 | 10606327 | TIR-NB-LRR |
| PCE_F_Chro8G0054100.2 | PCE_Fruticosa_Chro8 | 10599667 | 10606327 | TIR-NB-LRR |
| PCE_F_Chro8G0054700.1 | PCE_Fruticosa_Chro8 | 10673127 | 10690309 | TIR-NB-LRR |
| PCE_F_Chro8G0055200.1 | PCE_Fruticosa_Chro8 | 10743789 | 10751154 | TIR-NB-LRR |
| PCE_F_Chro8G0055200.2 | PCE_Fruticosa_Chro8 | 10743789 | 10751154 | TIR-NB-LRR |
| PCE_F_Chro8G0055600.1 | PCE_Fruticosa_Chro8 | 10779605 | 10782197 | SPTMK |
| PCE_F_Chro8G0056300.1 | PCE_Fruticosa_Chro8 | 10830332 | 10834244 | RLK |
| PCE_F_Chro8G0056800.1 | PCE_Fruticosa_Chro8 | 10900196 | 10904542 | TIR-NB-LRR |
| PCE_F_Chro8G0061100.1 | PCE_Fruticosa_Chro8 | 11377647 | 11381277 | SPTMK |
| PCE_F_Chro8G0061400.1 | PCE_Fruticosa_Chro8 | 11428201 | 11432408 | SPTMK |
| PCE_F_Chro8G0065500.1 | PCE_Fruticosa_Chro8 | 11899428 | 11902543 | CC-NB-LRR |
| PCE_F_Chro8G0067300.1 | PCE_Fruticosa_Chro8 | 12186100 | 12198349 | CC-NB-LRR |
| PCE_F_Chro8G0067600.1 | PCE_Fruticosa_Chro8 | 12217239 | 12220747 | CC-NB-LRR |
| PCE_F_Chro8G0067600.2 | PCE_Fruticosa_Chro8 | 12217239 | 12220747 | CC-NB-LRR |
| PCE_F_Chro8G0068100.1 | PCE_Fruticosa_Chro8 | 12289027 | 12291903 | CC-NB-LRR |
| PCE_F_Chro8G0068500.1 | PCE_Fruticosa_Chro8 | 12331823 | 12335531 | CC-NB-LRR |
| PCE_F_Chro8G0068500.2 | PCE_Fruticosa_Chro8 | 12331823 | 12335531 | CC-NB-LRR |
| PCE_F_Chro8G0068600.1 | PCE_Fruticosa_Chro8 | 12347214 | 12350370 | CC-NB-LRR |
| PCE_F_Chro8G0068600.2 | PCE_Fruticosa_Chro8 | 12347214 | 12350370 | CC-NB-LRR |
| PCE_F_Chro8G0074600.1 | PCE_Fruticosa_Chro8 | 13114031 | 13120077 | TIR-NB-LRR |
| PCE_F_Chro8G0074600.2 | PCE_Fruticosa_Chro8 | 13114031 | 13120077 | TIR-NB-LRR |
| PCE_F_Chro8G0074800.1 | PCE_Fruticosa_Chro8 | 13134182 | 13138481 | TIR-NB-LRR |
| PCE_F_Chro8G0074800.2 | PCE_Fruticosa_Chro8 | 13135443 | 13138481 | TIR-NB-LRR |
| PCE_F_Chro8G0074900.1 | PCE_Fruticosa_Chro8 | 13141539 | 13146210 | TIR-NB-LRR |
| PCE_F_Chro8G0074900.2 | PCE_Fruticosa_Chro8 | 13141539 | 13146210 | TIR-NB-LRR |
| PCE_F_Chro8G0076400.1 | PCE_Fruticosa_Chro8 | 13466906 | 13481530 | TIR-NB-LRR |
| PCE_F_Chro8G0076900.1 | PCE_Fruticosa_Chro8 | 13512496 | 13516948 | TIR-NB-LRR |
| PCE_F_Chro8G0077000.1 | PCE_Fruticosa_Chro8 | 13520478 | 13528910 | TIR-NB-LRR |
| PCE_F_Chro8G0077100.1 | PCE_Fruticosa_Chro8 | 13530804 | 13535767 | TIR-NB-LRR |
| PCE_F_Chro8G0077300.1 | PCE_Fruticosa_Chro8 | 13566413 | 13570748 | RLK |
| PCE_F_Chro8G0079700.1 | PCE_Fruticosa_Chro8 | 13801443 | 13804187 | CC-NB-LRR |
| PCE_F_Chro8G0088100.1 | PCE_Fruticosa_Chro8 | 15711090 | 15716013 | TIR-NB-LRR |
| PCE_F_Chro8G0088400.2 | PCE_Fruticosa_Chro8 | 15772708 | 15775020 | RLK |
| PCE_F_Chro8G0088400.3 | PCE_Fruticosa_Chro8 | 15772738 | 15775020 | RLK |
| PCE_F_Chro8G0092700.1 | PCE_Fruticosa_Chro8 | 16520700 | 16524094 | RLK |
| PCE_F_Chro8G0101100.1 | PCE_Fruticosa_Chro8 | 17960190 | 17963746 | CC-NB-LRR |
| PCE_F_Chro8G0103400.1 | PCE_Fruticosa_Chro8 | 18156880 | 18163680 | CC-NB-LRR |
| PCE_F_Chro8G0103800.1 | PCE_Fruticosa_Chro8 | 18214105 | 18218006 | CC-NB-LRR |
| PCE_F_Chro8G0104200.1 | PCE_Fruticosa_Chro8 | 18242688 | 18246217 | CC-NB-LRR |
| PCE_F_Chro8G0104500.1 | PCE_Fruticosa_Chro8 | 18296155 | 18299631 | CC-NB-LRR |
| PCE_F_Chro8G0104600.1 | PCE_Fruticosa_Chro8 | 18301757 | 18305286 | CC-NB-LRR |
| PCE_F_Chro8G0104700.1 | PCE_Fruticosa_Chro8 | 18308716 | 18312539 | CC-NB-LRR |
| PCE_F_Chro8G0104800.1 | PCE_Fruticosa_Chro8 | 18334423 | 18339211 | CC-NB-LRR |
| PCE_F_Chro8G0105400.1 | PCE_Fruticosa_Chro8 | 18360444 | 18364101 | CC-NB-LRR |
| PCE_F_Chro8G0105400.2 | PCE_Fruticosa_Chro8 | 18360444 | 18364107 | CC-NB-LRR |
| PCE_F_Chro8G0106400.1 | PCE_Fruticosa_Chro8 | 18471852 | 18475747 | CC-NB-LRR |
| PCE_F_Chro8G0115300.1 | PCE_Fruticosa_Chro8 | 19367388 | 19371198 | RLK |
| PCE_F_Chro8G0117200.1 | PCE_Fruticosa_Chro8 | 19637656 | 19643134 | TIR-NB-LRR |
| PCE_F_Chro8G0127400.1 | PCE_Fruticosa_Chro8 | 20563399 | 20571800 | TIR-NB-LRR |
| PCE_F_Chro8G0129400.1 | PCE_Fruticosa_Chro8 | 20732461 | 20740761 | TIR-NB-LRR |
| PCE_F_Chro8G0129700.1 | PCE_Fruticosa_Chro8 | 20761160 | 20766417 | TIR-NB-LRR |
| PCE_F_Chro8G0130400.1 | PCE_Fruticosa_Chro8 | 20806166 | 20811661 | TIR-NB-LRR |
| PCE_F_Chro8G0130400.2 | PCE_Fruticosa_Chro8 | 20806166 | 20811661 | TIR-NB-LRR |
| PCE_F_Chro8G0130500.1 | PCE_Fruticosa_Chro8 | 20817247 | 20822807 | TIR-NB-LRR |
| PCE_F_Chro8G0133700.1 | PCE_Fruticosa_Chro8 | 21016441 | 21019724 | RLK |
| PCE_F_Chro8G0150100.1 | PCE_Fruticosa_Chro8 | 22506269 | 22508869 | CC-NB-LRR |
| PCE_F_Chro8G0150200.1 | PCE_Fruticosa_Chro8 | 22513019 | 22515625 | CC-NB-LRR |
| PCE_F_Chro8G0150400.1 | PCE_Fruticosa_Chro8 | 22521576 | 22524160 | CC-NB-LRR |
| PCE_F_Chro8G0150900.1 | PCE_Fruticosa_Chro8 | 22559698 | 22562349 | CC-NB-LRR |
| PCE_F_Chro8G0151000.1 | PCE_Fruticosa_Chro8 | 22571521 | 22577743 | CC-NB-LRR |
| PCE_F_Chro8G0151100.3 | PCE_Fruticosa_Chro8 | 22574617 | 22590720 | SPTMK |
| PCE_F_Chro8G0151200.1 | PCE_Fruticosa_Chro8 | 22580836 | 22584596 | CC-NB-LRR |
| PCE_F_Chro8G0151100.1 | PCE_Fruticosa_Chro8 | 22585129 | 22590720 | SPTMK |
| PCE_F_Chro8G0151100.2 | PCE_Fruticosa_Chro8 | 22585129 | 22590720 | SPTMK |
| PCE_F_Chro8G0153600.1 | PCE_Fruticosa_Chro8 | 22873762 | 22876566 | CC-NB-LRR |
| PCE_F_Chro8G0153800.1 | PCE_Fruticosa_Chro8 | 22904514 | 22907315 | CC-NB-LRR |
| PCE_F_Chro8G0156000.1 | PCE_Fruticosa_Chro8 | 23080979 | 23084840 | RLK |
| PCE_F_Chro8G0160200.1 | PCE_Fruticosa_Chro8 | 23404046 | 23406953 | RLK |
| PCE_F_Chro8G0182800.1 | PCE_Fruticosa_Chro8 | 25183111 | 25190927 | RLK |
| PCE_F_Chro8G0187600.3 | PCE_Fruticosa_Chro8 | 25472052 | 25476037 | RLK |
| PCE_F_Chro8G0187600.2 | PCE_Fruticosa_Chro8 | 25472076 | 25476037 | RLK |
| PCE_F_Chro8G0187600.1 | PCE_Fruticosa_Chro8 | 25472091 | 25476037 | RLK |
| PCE_F_Chro8G0199400.1 | PCE_Fruticosa_Chro8 | 26315966 | 26320671 | TIR-NB-LRR |
| PCE_F_Chro8G0199400.2 | PCE_Fruticosa_Chro8 | 26315966 | 26320671 | TIR-NB-LRR |
| PCE_F_Chro8G0202200.1 | PCE_Fruticosa_Chro8 | 26556966 | 26559022 | LysM |
| PCE_F_Chro8G0205400.2 | PCE_Fruticosa_Chro8 | 26798436 | 26811209 | TIR-NB-LRR |
| PCE_F_Chro8G0205600.1 | PCE_Fruticosa_Chro8 | 26806667 | 26822898 | TIR-NB-LRR |
| PCE_F_Chro8G0205500.1 | PCE_Fruticosa_Chro8 | 26806667 | 26811110 | TIR-NB-LRR |
| PCE_F_Chro8G0205500.2 | PCE_Fruticosa_Chro8 | 26806667 | 26810936 | TIR-NB-LRR |
| PCE_F_Chro8G0211000.1 | PCE_Fruticosa_Chro8 | 27198154 | 27210640 | TIR-NB-LRR |
| PCE_F_Chro8G0211000.2 | PCE_Fruticosa_Chro8 | 27198154 | 27202807 | TIR-NB-LRR |
| PCE_F_Chro8G0211100.2 | PCE_Fruticosa_Chro8 | 27222984 | 27228000 | RLK |
| PCE_F_Chro8G0211100.4 | PCE_Fruticosa_Chro8 | 27222984 | 27228000 | RLK |
| PCE_F_Chro8G0211100.5 | PCE_Fruticosa_Chro8 | 27222984 | 27228000 | RLK |
| PCE_F_Chro8G0211100.1 | PCE_Fruticosa_Chro8 | 27222984 | 27228000 | RLK |
| PCE_F_Chro8G0212600.1 | PCE_Fruticosa_Chro8 | 27333607 | 27334496 | RLP |
| PCE_F_Chro8G0213100.1 | PCE_Fruticosa_Chro8 | 27347329 | 27348129 | RLP |
| PCE_F_Chro8G0213200.1 | PCE_Fruticosa_Chro8 | 27350120 | 27350875 | RLP |
| PCE_F_Chro8G0213300.1 | PCE_Fruticosa_Chro8 | 27352256 | 27355515 | RLK |
| PCE_F_Chro8G0213300.2 | PCE_Fruticosa_Chro8 | 27352256 | 27355515 | RLK |
| PCE_F_Chro8G0213300.3 | PCE_Fruticosa_Chro8 | 27352256 | 27355515 | RLK |
| PCE_F_Chro8G0228400.1 | PCE_Fruticosa_Chro8 | 28110286 | 28115640 | RLK |
| PCE_F_Chro8G0233800.1 | PCE_Fruticosa_Chro8 | 28419574 | 28422727 | RLK |
| PCE_F_Chro8G0234200.2 | PCE_Fruticosa_Chro8 | 28442630 | 28446284 | RLK |
| PCE_F_Chro8G0234200.3 | PCE_Fruticosa_Chro8 | 28442636 | 28446284 | RLK |
| PCE_F_Chro8G0244000.1 | PCE_Fruticosa_Chro8 | 29071880 | 29076030 | RLK |
| PCE_F_Chro8G0244500.1 | PCE_Fruticosa_Chro8 | 29109066 | 29112183 | RLK |
| PCE_F_Chro8G0244500.2 | PCE_Fruticosa_Chro8 | 29109066 | 29112183 | RLK |
| PCE_F_Chro8G0244600.1 | PCE_Fruticosa_Chro8 | 29123051 | 29126728 | RLK |
| PCE_F_Chro8G0244800.1 | PCE_Fruticosa_Chro8 | 29131553 | 29135681 | RLK |
| PCE_F_Chro8G0247600.1 | PCE_Fruticosa_Chro8 | 29324510 | 29326175 | RLP |
| PCE_F_Chro8G0267000.1 | PCE_Fruticosa_Chro8 | 30595835 | 30598370 | SPTMK |
| PCE_F_Chro8G0278100.1 | PCE_Fruticosa_Chro8 | 31290860 | 31295830 | SPTMK |
| PCE_F_Chro8G0278100.2 | PCE_Fruticosa_Chro8 | 31290860 | 31295830 | SPTMK |
| PCE_F_Chro8G0278100.4 | PCE_Fruticosa_Chro8 | 31290860 | 31295830 | SPTMK |
| PCE_F_Chro8G0278100.5 | PCE_Fruticosa_Chro8 | 31290860 | 31295830 | SPTMK |
| PCE_F_Chro8G0278100.6 | PCE_Fruticosa_Chro8 | 31290860 | 31295830 | SPTMK |
| PCE_F_Chro8G0278200.1 | PCE_Fruticosa_Chro8 | 31314027 | 31317654 | SPTMK |
| PCE_F_Chro8G0278500.1 | PCE_Fruticosa_Chro8 | 31354943 | 31368319 | SPTMK |
| PCE_F_Chro8G0278500.2 | PCE_Fruticosa_Chro8 | 31354943 | 31368319 | SPTMK |
| PCE_F_Chro8G0280000.1 | PCE_Fruticosa_Chro8 | 31418706 | 31423682 | TIR-NB-LRR |
